# How reputation does (and does not) drive people to punish without looking

**DOI:** 10.1073/pnas.2302475120

**Published:** 2023-07-05

**Authors:** Jillian J. Jordan, Nour S. Kteily

**Affiliations:** ^a^Negotiation, Organizations & Markets Unit, Harvard Business School, Boston, MA 02163; ^b^Management and Organizations Department, Kellogg School of Management, Northwestern University, Evanston, IL 60208

**Keywords:** opposing perspectives, outrage culture, signaling, ideology, moralistic punishment

## Abstract

Punishing wrongdoers can confer reputational benefits, and people sometimes punish without careful consideration. But are these observations related? Does reputation drive people to people to “punish without looking”? And if so, is this because *unquestioning* punishment looks particularly virtuous? To investigate, we assigned “Actors” to decide whether to sign punitive petitions about politicized issues (“punishment”), after first deciding whether to read articles opposing these petitions (“looking”). To manipulate reputation, we matched Actors with copartisan “Evaluators,” varying whether Evaluators observed i) nothing about Actors’ behavior, ii) whether Actors punished, or iii) whether Actors punished and whether they looked. Across four studies of Americans (total *n* = 10,343), Evaluators rated Actors more positively, and financially rewarded them, if they chose to (vs. not to) punish. Correspondingly, making *punishment* observable to Evaluators (i.e., moving from our first to second condition) drove Actors to punish more overall. Furthermore, because some of these individuals did not look, making punishment observable increased rates of punishment without looking. Yet punishers who eschewed opposing perspectives did not appear *particularly* virtuous. In fact, Evaluators preferred Actors who punished with (vs. without) looking. Correspondingly, making *looking* observable (i.e., moving from our second to third condition) drove Actors to look *more* overall—and to punish without looking at comparable or *diminished* rates. We thus find that reputation can encourage reflexive punishment—but simply as a byproduct of generally encouraging punishment, and not as a specific reputational strategy. Indeed, rather than fueling unquestioning decisions, spotlighting punishers’ decision-making processes may encourage reflection.

## Significance

Has an epidemic of “virtue signaling” left people too trigger-happy with respect to moralistic punishment? On the one hand, we find that placing reputational scrutiny on whether people punish does make people more punitive, and the punishment that it inspires is indeed sometimes unreflective. Yet, we also find that placing reputational scrutiny on *how* people come to punish (and particularly whether they first consider opposing perspectives) does not further increase rates of unreflective punishment. Moreover, when engaging with opposing perspectives is observable, overall rates of engagement increase, reflecting that people accurately expect considering opposing perspectives to look *good*. In these ways, our results paint a nuanced picture that both affirms and challenges critiques of virtue signaling and “outrage culture”.

Consider the following narrative: After an accusation of wrongdoing, an “outrage mob” appears. Initially, just a few condemn the accused. But before long, more and more people “pile on,” hastily meting out punishment with little regard for evidence or opposing perspectives.

According to some critics of “outrage culture,” such events have become troublingly frequent. A growing number of voices have expressed concern that society has become too quick to enact punishment without due consideration, yielding outcomes that can be disproportionate, unfair, and sometimes downright cruel (1, 2).

Even highly educated professors can, by their own admission, punish without sufficient reflection. In 2022, 38 Harvard faculty members signed an open letter condemning the University for sanctioning John Comaroff, an Anthropology Professor who was accused of sexual harassment. Their letter expressed “dismay” at the sanctions, endorsing Comaroff’s narrative (3). After more details emerged, however, 35 of the letter’s 38 signatories retracted their signatures, acknowledging that they were “lacking full information about the case.” Following the retraction, critics alleged that “the letter’s uncritical engagement with limited perspectives, voiced in such decisive and unambiguous terms, was hasty and uninformed” (4).

Here, we ask what drives people to punish without careful consideration, which we term “punishment without looking.” There are many ways that somebody might “look” (i.e., gather information to assess the merits of punishment) before deciding whether to punish: One might research basic facts, attempt to verify claims, question relevant sources, or consider additional perspectives—including those that support, or oppose, punishment. We conceptualize “punishment without looking” as the decision to punish without engaging in relevant looking behaviors. In particular, we focus on the form of punishment without looking that the 38 Harvard faculty members were accused of: punishment without consideration of opposing perspectives.

One might see a trend toward punishment with less consideration of opposing perspectives as negative or positive for society, depending on the context and one’s orientation toward the relevant moral issue. When merited, punishment is essential for discouraging wrongdoing and holding perpetrators accountable (5, 6). Consider how movements like #MeToo and #BlackLivesMatter have punished transgressions, like sexual assault and racism, that many believe have historically been committed with impunity. From this perspective, it is problematic when people are too *hesitant* to support justified punitive efforts. Regardless of whether one believes that people should punish more or less reflexively, however, punishment without looking clearly has meaningful social consequences—raising the question of what drives such behavior.

## Does Reputation Drive People to Punish without Looking?

We consider whether *reputation motives* (7–12) encourage punishment without looking. This hypothesis is partially inspired by social commentary suggesting that “virtue signaling” can inspire punishment that is hasty, underserved, or goes too far (13–15). For example, Sunstein (15) considers “what happens when a group of people, outraged by a real or imagined transgression, responds in a way that is disproportionate.” He suggests that such responding represents a “quick, automatic reaction,” fueled by reputation (“people want to appear at least as appalled as others in their social group.”) Similarly, Haidt and Rose-Stockwell (14) discuss “what happens when people use moral talk to enhance their prestige in a public forum,” arguing that “nuance and truth are casualties in this competition to gain the approval of the audience.” Such perspectives raise the question of whether reputation contributes to reflexive punishment.

Furthermore, academic research highlights the power of reputation to fuel punishment *in general.* People frequently engage in moralistic punishment and may punish by acting individually (e.g., a boss fires an employee) or collectively (e.g., an individual participates in the online shaming of a target or signs a petition calling for a target to be fired) (16–18). Such punishment can be socially rewarded (19) and signal trustworthiness (20–25) by conveying that the punisher is unlikely to themself transgress. Consequently, people punish more when their decisions are observable to others (26, 27) and when punishment has greater signaling value (20, 21).

Yet, while reputation clearly drives punishment in general, and social commentary suggests that virtue signaling may fuel reflexive punishment, the hypothesis that reputation drives people to punish specifically without looking remains untested. We investigate two distinct pathways through which reputation might have this effect.

First, reputation could drive punishment without looking as a *byproduct* of driving punishment in general. When reputation motivates people to punish, some individuals may choose not to look (e.g., to save time or effort or avoid engaging with disliked sources). Thus, by generally encouraging punishment, reputation may give rise to some reflexive punishment.

Second, reputation could drive punishment without looking as a *specific reputational strategy*, if people appear especially virtuous when they punish unquestioningly. People who punish without considering opposing perspectives might benefit from appearing particularly committed to the relevant moral cause—especially in politicized domains, where people are increasingly intolerant of other viewpoints (28). Furthermore, theoretical modeling highlights that cooperating “without looking” (i.e., without attending to the costs of cooperation) can be preferentially rewarded (29). Indeed, cooperative decisions that are faster, more intuitive, and less calculating tend to be evaluated more positively (9, 30–32), and people make less calculating cooperative decisions when others can observe their decision-making process (30). Thus, cooperating without looking can serve as a specific reputational strategy, and punishing without looking might function similarly.

Yet, there are also reasons to doubt this proposal. Punishment—unlike cooperation—is morally bad when undeserved, so punishers who eschew opposing perspectives might incur the reputational *cost* of seeming less fair-minded. Furthermore, deliberative decisions are often extolled as wiser (33–35), so punishment without looking might signal reduced competence.

Across four preregistered studies of Americans from MTurk and Prolific (total *n* = 10,343), we show that reputation can drive people to punish without looking. Critically, however, we find that reputation fuels such punishment as a byproduct of incentivizing punishment in general and not as a specific reputation strategy. While punishers were reputationally rewarded in our studies, punishers earned the best reputations by considering opposing perspectives before punishing. Correspondingly, placing reputational scrutiny on punishment in general increased punishment without looking. But spotlighting potential punishers’ decision-making processes actually encouraged engagement with other perspectives—and did not influence or even decreased punishment without looking.

We also consider how the influence of reputation may depend on the reputational audience and their ideological conviction toward the relevant moral issue. People may frequently face audiences who share their ideological viewpoints (i.e., “homophily”) (36, 37) and in particular hold extreme aligned views (i.e., “acrophily”) (38). Interestingly, in our studies, even ideologically extreme audiences did not preferentially reward punishers who declined (vs. chose) to look. Yet, we find evidence that such audiences may nonetheless preferentially fuel punishment without looking—both by doing more to encourage punishment in general and less to encourage consideration of other viewpoints.

## Paradigm Overview.

To investigate punishment without looking, we designed an incentive-compatible paradigm with meaningful stakes. We invited Actor subjects to sign real punitive petitions about politicized moral issues from the website Change.org (“punishment”), with or without first reading articles expressing perspectives opposing the petitions (“looking”). And we invited Evaluator subjects to respond to Actors’ decisions, while allocating financial resources to them.

We assigned Democrat vs. Republican subjects to engage with distinct petitions, that we expected to resonate with their respective political parties. Democrats read one of two petitions (because the first closed before we finished conducting all studies in this paper), calling for the firing of either i) University of Central Florida professor Charles Negy, in light of “abhorrent racist comments…on his personal Twitter account,” or ii) Los Angeles Police Department Chief Michel Moore, following Moore’s statement that the death of George Floyd is on protestors’ and looters’ hands. These petitions align with the #BlackLivesMatter movement, which is preferentially supported by liberal Americans. Republicans read a petition calling for the removal of “Blue Lives Murder” merchandise from Amazon, which the petition characterized as “hatred merchandise.” This petition aligns with the #BlueLivesMatter countermovement, which is preferentially supported by conservative Americans. Because these petitions had the potential to trigger outcomes that were costly to their targets, we conceptualize signing the petitions as acts of (collective) punishment.

Before Actors decided whether to sign the petitions, they could consider opposing perspectives. We presented Actors with links to two real news articles with headlines suggesting that punishment might be unmerited (e.g., “UCF professor behind tweets deemed racist says he is subject of ‘witch hunt’” for the Negy petition; “Despite criticism, LAPD Chief Michel Moore maintains support in political circles” for the Moore petition; “Spring woman says ‘Blue Lives Murder’ shirts for sale on Amazon are hate speech. Activist disagrees” for the Amazon petition). Actors could read these articles and also search the Internet for other opposing perspectives.

We used this paradigm to track whether Actors looked (i.e., considered opposing perspective articles) and punished (i.e., signed their assigned petition) and thus to identify individuals who punished without looking. To incorporate reputation, we used a “Dictator Game”: Evaluators received an endowment of 50¢ and decided how much to share with an Actor of their same political affiliation, on the basis of the Actor’s study decisions. Of note, stakes of this magnitude in online economic game studies tend to produce comparable results to in-person studies with stakes ten times larger (39–41).

In studies 1 and 2, we used this paradigm to ask how *Evaluators* create reputational incentives for Actors, when rewarding their behavior. In these studies, Evaluators decided how much money to share, if given different information (or no information) about an Actor’s behavior [the “strategy method” (42)]. They also rated Actors, using 0 to 100 scales, on overall positivity of impression, fairness, competence, and loyalty to the relevant moral cause. In studies 3 and 4, we turned to asking how reputational incentives shape the behavior of *Actors*. In these studies, we compared Actors’ punishment and looking behavior across conditions in which Evaluators could observe i) nothing about their decisions (“Nothing Observable”), ii) just whether they punished (“Punishment Observable”), or iii) whether they punished *and* whether they looked (“Both Observable”). For all studies, we separately report results among Democrats vs. Republicans, given that we presented these groups with different petitions (and opposing perspective articles); importantly, although we expected our materials to broadly trigger a similar psychology among Democrats and Republicans, any differences in the magnitude of effects among these groups may reflect that the stimuli they received were not exactly the same, rather than inherent differences in their psychological or behavioral tendencies.

## Results

### Study 1.

Study 1 investigated Evaluators. In study 1a, we assigned *n* = 629 Democrat Evaluators to Actors who had either considered the Negy (*n* = 308) or Moore (*n* = 321) petition. Both groups showed comparable results (*SI Appendix*, section 2.1), so our analyses collapse across petition. In study 1b, we assigned *n* = 600 Republican Evaluators to Actors who had considered the Amazon petition. To introduce the concept of looking to Evaluators, we explained that before deciding whether to sign their petition, Actors could read opposing perspective articles and/or search the Internet for other opposing perspectives. We also showed Evaluators an example article headline (that their Actor was shown) and explained how it constituted an opposing perspective.

#### Did Evaluators Create Reputational Incentives for Punishment in General?

**Fig. 1.**
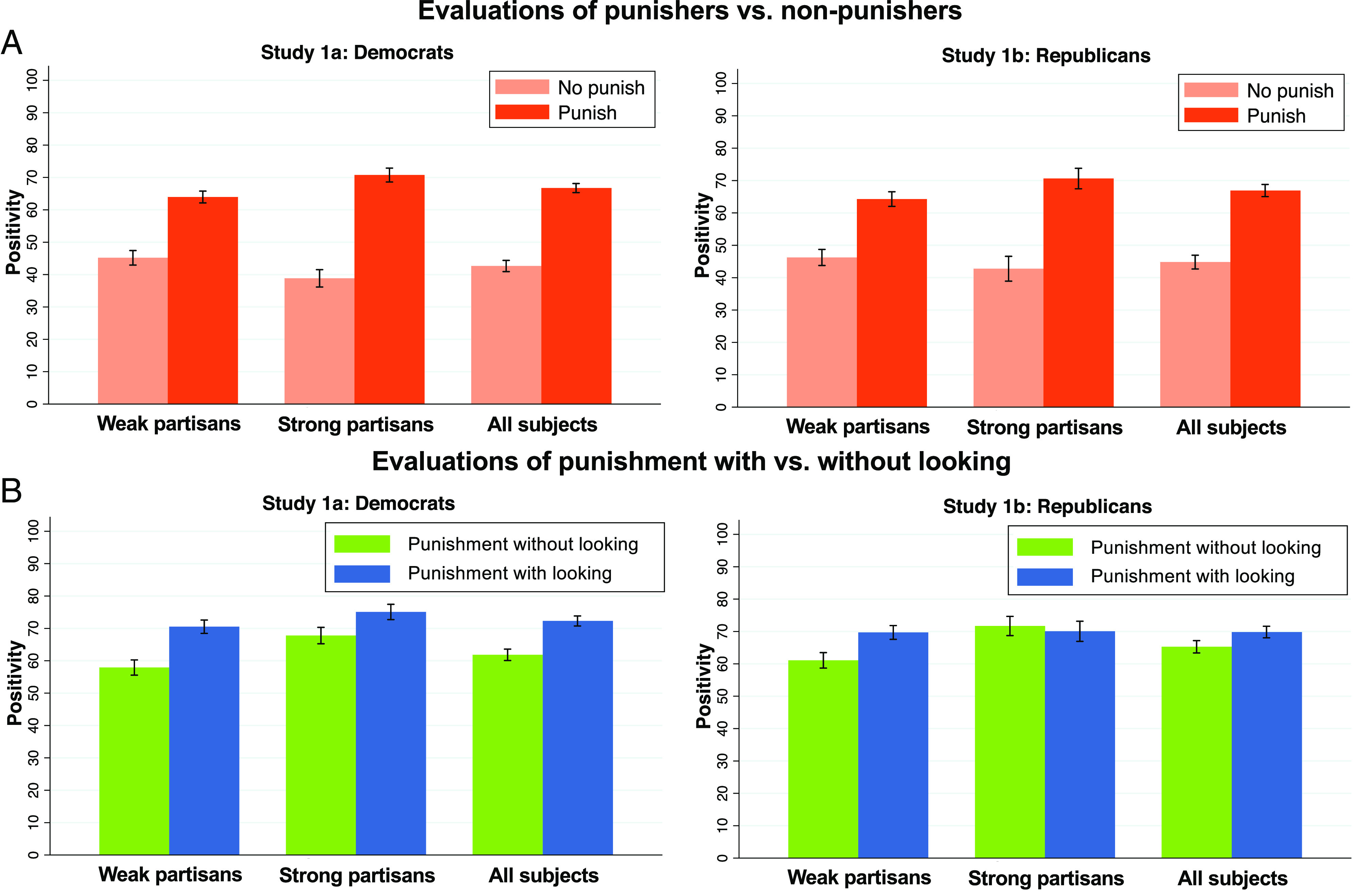
Evaluators react positively to punishment but do *not* prefer punishment without looking. Shown are evaluations of Actors who (*A*) did vs. did not punish and (*B*) punished with vs. without looking, among weak partisan Evaluators, strong partisan Evaluators, and all Evaluators, in study 1. Bars plot mean positivity ratings, and error bars are 95% CIs. For a version of Fig. 1 that plots money shared, see *SI Appendix*, section 2.2.

We first ask: Did Evaluators create reputational incentives for punishment *in general*, as must be true for even the “byproduct” hypothesis to hold? We thus compare Evaluators’ reactions to Actors who did vs. did not punish (described as signing vs. not signing their petition), when given no information about whether the Actor looked (Fig. 1*A*).

In study 1a, Democrats formed more positive overall impressions of Actors who *did* (vs. did not) punish (*b* = 24.08 [21.55, 26.61], *t* = 18.69, *P* < 0.001), shared a larger percentage of their endowment with punishers (*b* = 15.80 [13.79, 17.81], *t* = 15.44, *P* < 0.001), and rated punishers as more loyal supporters of #BlackLivesMatter (*b* = 36.82 [34.59, 39.05], *t* = 32.47, *P* < 0.001), more fair (*b* = 19.23 [17.06, 21.41], *t* = 17.38, *P* < 0.001), and more competent (*b* = 17.42 [15.37, 19.46], *t* = 16.71, *P* < 0.001), *n* = 629.

Similarly, Republicans in study 1b rated punishers more positively overall (*b* = 22.10 [18.77, 25.42], *t* = 13.05, *P* < 0.001), shared more money with punishers (*b* = 15.32 [12.81,17.82], *t* = 12.02, *P* < 0.001), and rated punishers as more loyal supporters of #BlueLivesMatter (*b* = 26.91 [23.49, 30.34], *t* = 15.45, *P* < 0.001), more fair (*b* = 19.86 [17.15, 22.58], *t* = 14.37, *P* < 0.001), and more competent (*b* = 18.02 [15.45, 20.58], *t* = 13.80, *P* < 0.001), *n* = 600.

Thus, Evaluators rewarded punishers, creating incentives for Actors to punish in general. We also find that Evaluators who identified as “strong” (vs. “weak”) partisans showed relatively stronger preferences for punishers (vs. nonpunishers) (Fig. 1*A* and Table 1), suggesting that more ideological audiences create especially strong incentives for punishment.

**Table 1.** Strong partisan Evaluators react more positively to punishment, and less negatively to punishment without looking

	Evaluations of punishers vs. nonpunishers (Positive coefficients reflect preferences for *punishment*)		Evaluations of punishment without vs. with looking (Positive coefficients reflect preferences for punishment *without* looking)
Democrats (Study 1a)			
Positivity	Weak Partisans: *b* = 18.78 [15.54, 22.02], *t* = 11.39, *P* < 0.001	Weak Partisans: *b* = −12.61 [−15.08, −10.14], *t* = −10.05, *P* < 0.001	
	Strong Partisans: *b* = 31.90 [27.95, 35.85], *t* = 15.90, *P* < 0.001	Strong Partisans: *b* = −7.30 [−10.09, −4.51], *t* = −5.16, *P* < 0.001	
	Interaction: *b* = 13.12 [8.03, 18.22], *t* = 5.06, *P* < 0.001	Interaction: *b* = 5.31 [1.60, 9.02], *t* = 2.81, *P* = 0.005	
Sharing	Weak Partisans: *b* = 11.78 [9.30, 14.25], *t* = 9.35, *P* < 0.001	Weak Partisans: *b* = −6.23 [−8.12, −4.34], *t* = −6.49, *P* < 0.001	
	Strong Partisans: *b* = 22.00 [18.68, 25.32], *t* = 13.05, *P* < 0.001	Strong Partisans: *b* = −1.53 [−3.79, 0.73], *t* = −1.33, *P* = 0.183	
	Interaction: *b* = 10.22 [6.09, 14.35], *t* = 4.86, *P* < 0.001	Interaction: *b* = 4.70 [1.77, 7.63], *t* = 3.15, *P* = 0.002	
Republicans (Study 1b)			
Positivity	Weak Partisans: *b* = 18.02 [14.07, 21.98], *t* = 8.97, *P* < 0.001	Weak Partisans: *b* = −8.62 [−10.69, −6.55], *t* = −8.18, *P* < 0.001	
	Strong Partisans: *b* = 27.86 [22.04, 33.68], *t* = 9.43, *P* < 0.001	Strong Partisans: *b* = 1.64 [−1.36, 4.63], *t* = 1.07, *P* = 0.284	
	Interaction: *b* = 9.84 [2.83, 16.85], *t* = 2.76, *P* = 0.006	Interaction: *b* = 10.26 [6.62, 13.89], *t* = 5.54, *P* < 0.001	
Sharing	Weak Partisans: *b* = 11.77 [8.78, 14.76], *t* = 7.74, *P* < 0.001	Weak Partisans: *b* = −4.02 [−5.78, −2.25], *t* = −4.47, *P* < 0.001	
	Strong Partisans: *b* = 20.72 [16.37, 25.07], *t* = 9.38, *P* < 0.001	Strong Partisans: *b* = 1.78 [−0.59, 4.15], *t* = 1.48, *P* = 0.140	
	Interaction: *b* = 8.95 [3.69, 14.21], *t* = 3.34, *P* = 0.001	Interaction: *b* = 5.80 [2.85, 8.74], *t* = 3.87, *P* < 0.001	

In the left columns, for both positivity and money shared, we predict evaluations of Actors who did vs. did not punish. We report simple effects of punishment among both weak and strong partisan Evaluators, as well as the interaction between partisanship and punishment. In the right columns, we predict evaluations of Actors who punished without vs. with looking; we report simple effects of not looking, as well as the interaction between partisanship and not looking. Of note, a small number of subjects in study 1 did not complete our measure of strong vs. weak partisanship (due to an error, answering this question was not required for some of the time that study 1 was active); Table 1 thus reports results from the *n* = 621 Democrats (study 1b) and *n* = 597 Republicans (study 1b) who did complete the measure.

#### Did Evaluators Create Reputational Incentives to Punish Specifically without Looking?

Next, we ask: Did Evaluators create reputational incentives for punishment *specifically without looking,* as the “specific reputational strategy” hypothesis predicts? We thus compare Evaluators’ reactions to Actors who punished, without vs. with looking (described as spending a below vs. above average amount of time considering opposing perspectives) (Fig. 1*B*).

In study 1a, Democrats formed *less* positive impressions of punishers who declined (vs. chose) to look (*b* = −10.46 [−12.32, −8.60], *t* = −11.07, *P* < 0.001) and shared less money with them (*b* = −4.39 [−5.83, −2.94], *t* = −5.96, *P* < 0.001). They also rated punishers who declined to look as less fair (*b* = −16.30 [−18.35, −14.25], *t* = −15.61, *P* < 0.001) and less competent (*b* = −11.10 [−12.81, −9.39], *t* = −12.73, *P* < 0.001) but as *more* loyal (*b* = 5.55 [3.95, 7.14], *t* = 6.82, *P* < 0.001). Similarly, Republicans in study 1b rated punishers who declined to look less positively (*b* = −4.55 [−6.30, −2.79], *t* = −5.08, *P* < 0.001), shared less money with them (*b* = −1.72 [−3.14, −0.29], *t* = −2.36, *P* = 0.018), and saw them as less fair (*b* = −8.62 [−10.38, −6.86], *t* = −9.62, *P* < 0.001) and less competent (*b* = −5.20 [−6.87, −3.52], *t* = −6.10, *P* < 0.001) but also as more loyal (*b* = 6.90 [4.91, 8.88], *t* = 6.83, *P* < 0.001).

Thus, Evaluators did see punishing without looking as an especially strong signal of loyalty. Yet, eschewing opposing perspectives also had reputational costs: It made punishers seem less fair and less competent. And the reputational costs outweighed the benefits, such that Evaluators rated punishers who declined to look less positively overall and sent them less money. Indeed, nonpreregistered mediation analyses reveal i) *positive* indirect effects of declining to look on overall positivity via enhanced ratings of loyalty and ii) countervailing (and significantly larger) *negative* indirect effects via diminished ratings of fairness and competence (*SI Appendix*, section 2.3).

Our design also allows for between-subjects analyses of responses to punishers who declined vs. chose to look (because Evaluators encountered these two Actor profiles first, in random order). These analyses were also preregistered and produce results that are similar to our within-subject analyses, but, in line with their reduced statistical power, are less consistently significant (*SI Appendix*, section 2.4).

We also again investigate the role of Evaluator ideology (Fig. 1*B* and Table 1). We find that strong (vs. weak) partisans reacted relatively less negatively to punishment without (vs. with) looking. Still, even strong partisans did not *prefer* punishment without looking. Strong Democrats rated punishers significantly less positively if they declined (vs. chose) to look but shared comparable amounts of money with both types of punishers; strong Republicans did not significantly differentiate between punishers who declined vs. chose to look on either global evaluation measure.

Finally, study 1 reveals that Evaluators reacted positively to looking even when the Actor ultimately declined to punish; see *SI Appendix*, section 2.5 for details.

### Study 2.

**Fig. 2.**
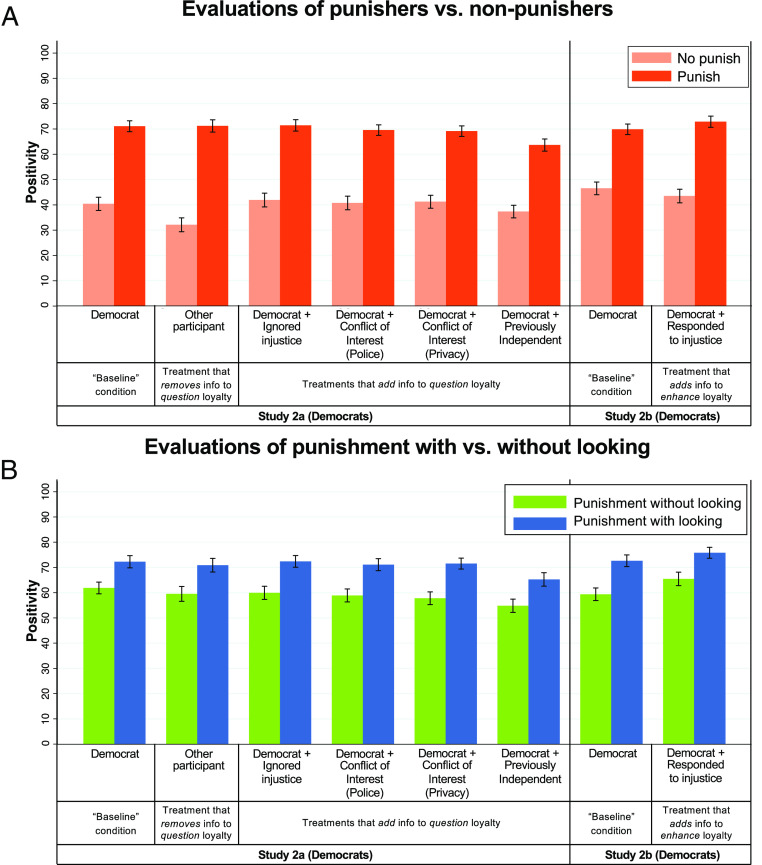
Evaluators do not prefer punishment without looking, even when given reason to doubt or trust the Actor’s loyalty. Shown are evaluations of Actors who did vs. did not punish, and who punished with vs. without looking, across conditions in study 2. (*A*) Evaluators in all conditions preferred punishers to nonpunishers, and this preference was significantly stronger in the “Other Participant” and “Responded to injustice” treatments. (*B*) Evaluators in all conditions dispreferred punishers who declined (vs. chose) to look, and no treatments significantly mitigated the reputation costs of not looking. Bars plot mean positivity ratings, and error bars are 95% CIs. For a version of Fig. 2 that plots money shared, see *SI Appendix*, section 3.1.

In study 1, Evaluators created reputational incentives for punishment in general, highlighting the potential for reputation to drive punishment without looking as a byproduct. However, Evaluators in study 1 did not preferentially reward punishment without (vs. with) looking, casting doubt on the hypothesis that reputation drives punishment without looking as a specific reputational strategy. In study 2, which specifically recruited Democrat subjects, we probed the robustness of this conclusion (Fig. 2).

Given that punishment without looking can serve as a strong signal of loyalty, we might expect it to pay greater reputational dividends when one’s loyalty is in doubt. In study 2a, we thus asked: Do Evaluators reward punishment without looking when given reason to doubt the Actor’s loyalty? To investigate, we assigned *n* = 1,796 Democrat Evaluators (across six conditions) to Actors who had considered the Moore petition. In the “baseline condition,” mirroring study 1, Evaluators learned that their Actor was a Democrat. In the “Other participant” treatment, by contrast, Evaluators merely learned that their Actor was another participant—leaving the Actor’s values (and thus loyalty toward the petition’s cause) completely ambiguous. Finally, in four different treatments, Evaluators learned that their Actor was a Democrat but also received additional information: They saw a (real) screenshot of their Actor responding to survey question(s). In each of these treatments, we anticipated that the responses in the screenshot would call the Actor’s loyalty into question.

Specifically, in the “Ignored injustice” treatment, the featured Actor described witnessing racial injustice but doing nothing to respond. In two “Conflict of Interest” treatments, the Actors described reasons that signing the petition—which advocated firing Police Chief Moore—might conflict with their personal self-interest. (In the “Police” version of this treatment, the Actor reported having a police officer uncle; in the “Privacy” version, the Actor noted that signing could make their information public, eliciting spam.) Finally, in the “Previously Independent” treatment, the Actor described a questionable history of partisan commitment (in which they voted in just one of the last four elections—and, before becoming a Democrat, identified as an Independent and “praised Trump”).

Thus, across five treatments, we attempted to cast doubt on Actor loyalty, either by removing or adding information—and four of our five treatments successfully achieved this aim (Table 2A). Yet, none of our treatments caused Evaluators to reward punishment without looking as a specific reputation strategy. In all six conditions of study 2a, Evaluators reacted significantly more negatively to Actors who punished without (vs. with) looking. And none of our five treatments significantly mitigated the reputation costs of not looking, relative to the baseline condition.

**Table 2.** Study 2 results

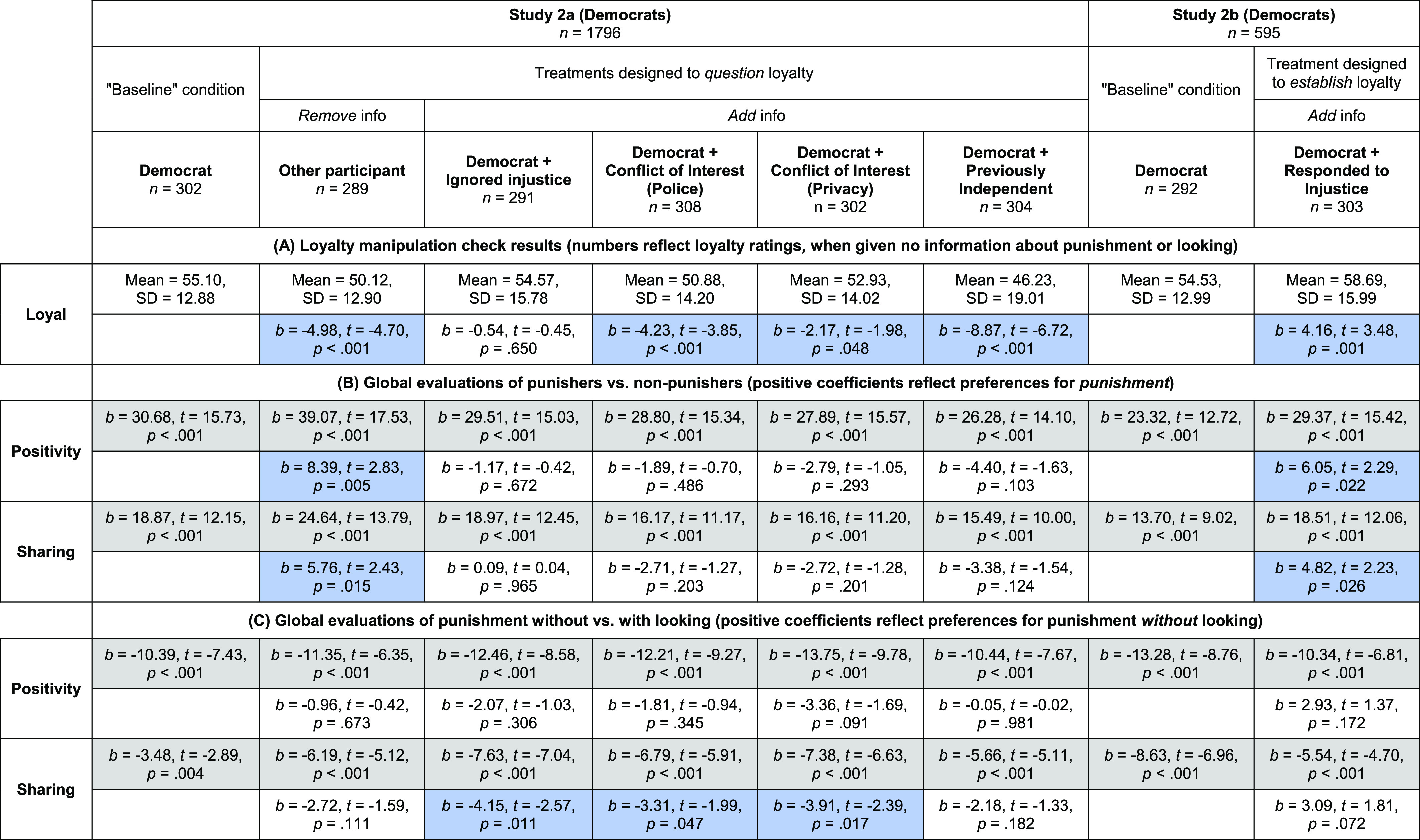	

(*A*) Treatments in Study 2 successfully cast doubt on (Study 2a) or highlighted (Study 2b) Actor loyalty. As manipulation checks, we analyze Evaluators’ ratings of Actor loyalty, when given no information about the Actor’s punishment or looking behavior. We report descriptive statistics within each condition (first rows) and, for each treatment, compare ratings in the treatment vs. baseline condition (second rows; significant differences highlighted in blue). (*B*) Some treatments increased the reputation value of punishment in general. We analyze evaluations of Actors who did vs. did not punish. For both positivity and sharing, we report simple effects of Actor punishment within each condition (first rows; significant effects highlighted in grey) and, for each treatment, compare the effect of punishment in the treatment vs. baseline condition (i.e., we test for punishment X treatment interactions) (second rows; significant interactions highlighted in blue). (*C*) No treatments caused Evaluators to prefer punishment without (vs. with) looking. We repeat the above approach, but analyze evaluations of Actors who punished without vs. with looking (and report simple effects of not looking, and not looking X treatment interactions). For a version of Table 2 with CIs on regression coefficients (not shown here for readability), see *SI Appendix*, section 3.2.

Moreover, three study 2a treatments (“Ignored injustice” and both “Conflict of Interest” treatments) actually caused Evaluators to share relatively *less* money with punishers who declined (vs. chose) to look (Table 2C and Fig. 2*B*). This result is particularly notable for the two of these treatments for which our loyalty manipulation check was significant (i.e., our two “Conflict of Interest” treatments) and especially for our “Conflict of Interest: Police” treatment, which had a particularly clear negative influence on perceived loyalty. This latter treatment thus highlights that background information about an Actor can simultaneously i) strongly call their loyalty into question and ii) *decrease* the reputation value of punishment without looking. Supplementary analyses of study 2 (*SI Appendix*, section 3.3) suggest that this finding may reflect that when given active reason to doubt an Actor’s loyalty, Evaluators cease to see punishment without looking as a positive signal of loyalty—perhaps because they become more inclined to interpret not looking as laziness, rather than moral commitment.

Taking the reverse approach—and establishing a *positive* track record of Actor loyalty—also did not cause Evaluators to preferentially reward punishment without looking. In study 2b (*n* = 595 Evaluators across two conditions), we compared a baseline condition to a treatment in which we added information to highlight the Actor’s loyalty. Specifically, we described the Actor as a Democrat and provided a screenshot where the Actor described *responding* to racial injustice. This “Responded to injustice” treatment increased perceived Actor loyalty (Table 2A). Yet, in both conditions of study 2b, Evaluators again reacted more negatively to punishment without (vs. with) looking, and our treatment (vs. baseline condition) did not significantly mitigate the reputation costs of not looking (although we do observe a marginally significant effect in this direction for our money shared DV) (Table 2C and Fig. 2*B*). Supplementary analyses suggest that highlighting Actor loyalty did make punishment without looking seem less unfair (perhaps because it seemed less hasty or uninformed) but also diminished its value as a signal of loyalty (perhaps because loyalty was already well established)—such that the overall reputation value of punishment without looking did not significantly change.

While our study 2 treatments did not cause Evaluators to reward punishment without looking as a specific reputation strategy, some of them did increase the reputation value of punishment in general (Table 2B and Fig. 2*A*). In all seven conditions of study 2, Evaluators reacted more positively to Actors who did (vs. did not) punish—but this preference for punishment was significantly stronger, relative to the relevant baseline condition, in both the “Other participant” treatment of study 2a and the “Responded to justice” treatment of study 2b.

Together, study 2 thus reveals that Actors may gain more from punishing *in general* when their loyalty is in doubt or is particularly well established—but they are still unlikely to be preferentially rewarded for punishing *without looking*. These results cast further doubt on the hypothesis that reputation drives punishment without looking as a specific reputational strategy.

Finally, we note that the baseline conditions of studies 2a–b replicated all reported study 1 effects on fairness, competence, and loyalty—including our mediation analyses, which we preregistered in study 2. Study 2 also provides further evidence, both within the baseline conditions and overall, for the moderating effects of Evaluator ideology (whereby strong partisans react more positively to punishment in general, and less negatively to punishment without looking). Across all conditions of study 2, however, even strong partisan Evaluators did not *prefer* punishment without looking. See *SI Appendix*, sections 3.3–3.5 for more information.

### Studies 3 and 4.

In studies 1 and 2, Evaluators rewarded punishment in general but not punishment specifically without looking. These studies thus highlight the reputational incentives that Evaluators create for Actors. In studies 3 and 4, we transition to investigating how such reputational incentives do (and do not) drive Actors to punish without looking. We thus shift our focus from Evaluators to Actors. We measured whether Actors chose to look (defined as clicking a link to at least one opposing perspective article) and punish (defined as signing their petition), allowing us to categorize Actors who punished without looking.

We consider two distinct reputational contexts that varied with respect to audience ideology. In studies 3a (*n* = 1,808 Democrat Actors who considered the Moore petition) and 3b (*n* = 1,796 Republican Actors who considered the Amazon petition), Actors were assigned to a copartisan Evaluator who identified as “a weak [Democrat/Republican], who only leans toward the party”. In studies 4a (*n* = 1,974 Democrat Actors who considered the Negy petition) and 4b (*n* = 1,145 Republican Actors who considered the Amazon petition), Actors learned that their copartisan Evaluator identified as “a strong [Democrat/Republican] who strongly supports [Black Lives Matter/Blue Lives Matter]”. Thus, Actors faced more ideological audiences in study 4 than study 3.

Of note, our study 4a data were collected in two batches; however, all key results i) held significantly within the first batch and ii) survive corrections for “peeking” between batches (*SI Appendix*, section 4.1).

#### Does Making Punishment Observable Drive Punishment without Looking?

**Fig. 3.**
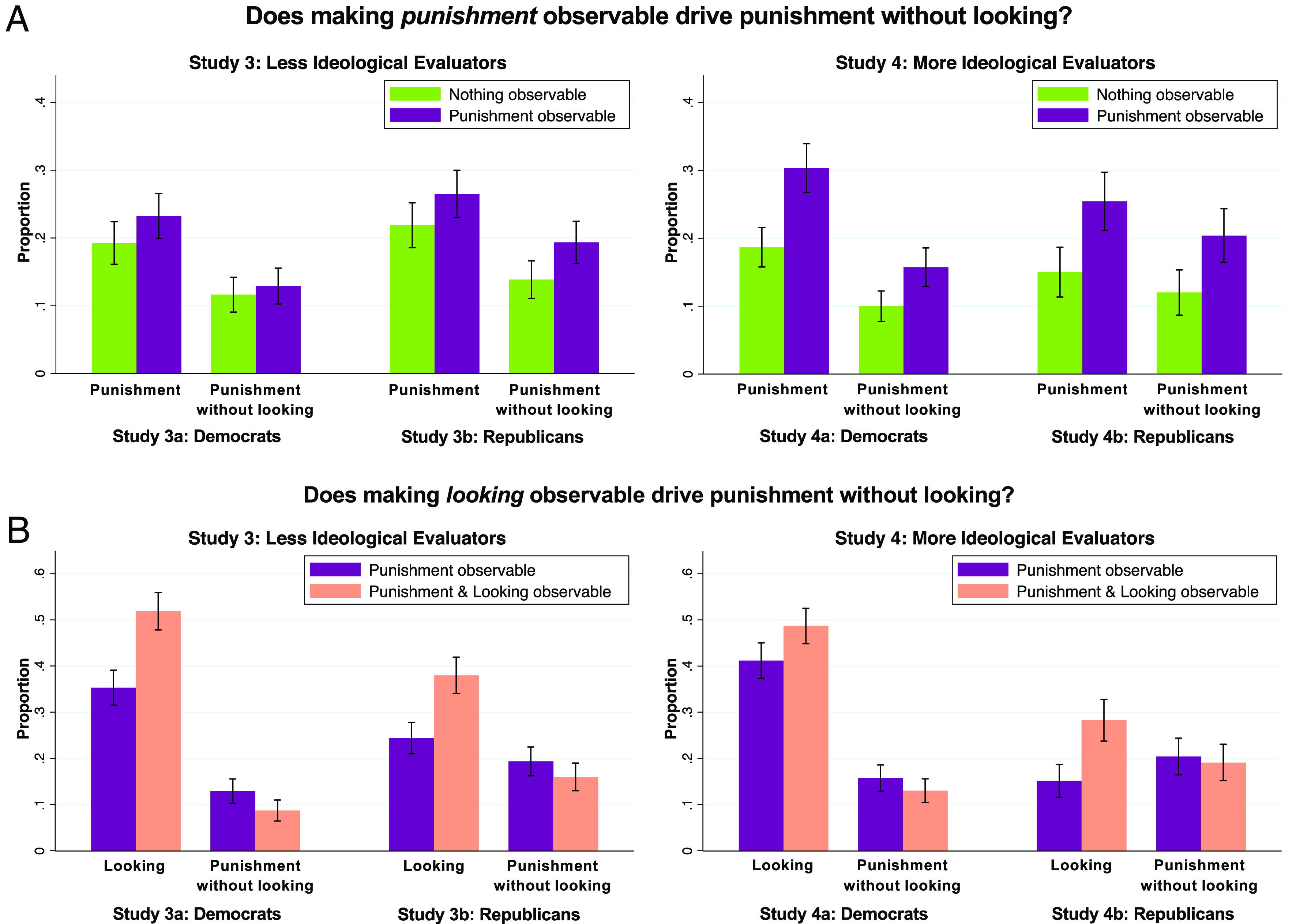
Making punishment observable drives punishment without looking, while making looking observable does not. (*A*) Placing reputational scrutiny on *punishment* (by making punishment observable) drove Actors to punish more overall and to punish specifically without looking. We plot the proportion of subjects who chose to i) punish and ii) punish without looking, across the Nothing Observable vs. Punishment Observable conditions. (*B*) Placing reputational scrutiny on *looking* (by making looking observable) increased rates of looking overall and did not drive punishment without looking. We plot the proportion of subjects who chose to i) look and ii) punish without looking, across the Punishment Observable vs. Both Observable conditions. Error bars are 95% CIs.

We begin by investigating whether reputation drives Actors to punish without looking as a byproduct of incentivizing punishment in general. We thus ask: Does making *punishment* observable to Evaluators—thereby placing reputational scrutiny on whether Actors punish in general*—*encourage punishment without looking? To answer, we compare the “Nothing Observable” and “Punishment Observable” conditions, which differed only in whether punishment was observable (Fig. 3*A*).

##### Study 3.

Before analyzing punishment without looking, we ask whether making punishment observable increased punishment *overall*. We find that, in study 3, Democrats were marginally significantly more likely to punish in Punishment Observable (23%) than Nothing Observable (19%), *b* = 0.04 [−0.01, 0.09], *t* = 1.69, *P* = 0.091, *n* = 1,222. Similarly, Republicans were marginally significantly more likely to punish in Punishment Observable (27%) than Nothing Observable (22%), *b* = 0.05 [−0.002, 0.09], *t* = 1.89, *P* = 0.060, *n* = 1,214.

Next, we directly test the byproduct hypothesis by asking: Did making punishment observable increase rates of punishment *without looking*? Among Democrats, rates of punishment without looking were comparable across the Punishment Observable (13%) and Nothing Observable (12%) conditions, *b* = 0.01 [−0.02, 0.05], *t* = 0.68, *P* = 0.497, *n* = 1,222. However, Republicans were significantly more likely to punish without looking in Punishment Observable (19%) than Nothing Observable (14%), *b* = 0.05 [0.01, 0.10, *t* = 2.57, *P* = 0.010, *n* = 1,214.

Thus, in study 3—which featured less ideological Evaluators—making punishment observable had marginally significant effects on punishment overall and increased punishment without looking among Republicans but not Democrats.

##### Study 4.

In study 4, making punishment observable significantly increased punishment overall. Democrats were more likely to punish in Punishment Observable (30%) than Nothing Observable (19%), *b* = 0.12 [0.07, 0.16], *t* = 4.98, *P* < 0.001, *n* = 1,319. Similarly, Republicans were more likely to punish in Punishment Observable (25%) than Nothing Observable (15%), *b* = 0.10 [0.05, 0.16], *t* = 3.59, *P* < 0.001, *n* = 763.

Making punishment observable also significantly increased punishment without looking. Among Democrats, rates of punishment without looking were higher in Punishment Observable (16%) than Nothing Observable (10%), *b* = 0.06 [0.02, 0.09], *t* = 3.13, *P* = 0.002, *n* = 1,319. Similarly, Republicans were more likely to punish without looking in Punishment Observable (20%) than Nothing Observable (12%), *b* = 0.08 [0.03, 0.14], *t* = 3.14, *P* = 0.002, *n* = 763.

Thus, in study 4—which featured more ideological Evaluators—making punishment observable increased punishment overall and punishment without looking specifically.

Together, studies 3–4 suggest that reputation *can* fuel punishment without looking by incentivizing punishment in general. Furthermore, consistent with our finding from studies 1–2 that more ideological Evaluators create stronger reputational incentives for punishment, we found stronger evidence for this process in study 4 than study 3.

#### Does Making Looking Observable Drive Punishment without Looking?

Next, we investigate whether reputation drives punishment without looking as a specific reputational strategy. We thus ask: Does additionally making *looking* observable—thereby placing reputational scrutiny on *how* Actors come to their punishment decisions—further encourage punishment without looking? To answer, we compare the “Punishment Observable” and “Both Observable” conditions, which differed only in whether looking was observable (Fig. 3*B*).

##### Study 3.

Before analyzing rates of punishment without looking, we ask whether making looking observable influenced looking *overall*. In study 3, Democrats were significantly *more* likely to look in Both Observable (52%) than Punishment Observable (35%), *b* = 0.17 [0.11, 0.22], *t* = 5.88, *P* < 0.001, *n* = 1,206. Similarly, Republicans were more likely to look in Both Observable (38%) than Punishment Observable (24%), *b* = 0.14 [0.08, 0.19], *t* = 5.13, *P* < 0.001, *n* = 1,197. These results suggest that Actors expected Evaluators to *reward* them for considering opposing perspectives—and thus that placing reputational scrutiny on looking is unlikely to encourage punishment without looking.

Next, we directly test how making looking observable influenced punishment without looking. Among Democrats, we find significantly *lower* rates of punishment without looking in Both Observable (9%) than Punishment Observable (13%), *b* = −0.04 [−0.08, −0.01], *t* = −2.35, *P* = 0.019, *n* = 1,206. And among Republicans, we find no significant difference between rates of punishment without looking in Both Observable (16%) and Punishment Observable (19%), *b* = −0.03 [−0.08, 0.01], *t* = −1.53, *P* = 0.127, *n* = 1,197.

Thus, study 3—which featured less ideological Evaluators—provides no evidence that making looking observable encourages punishment without looking. Rather, making looking observable *increased* looking overall, and, among Democrats, significantly *decreased* punishment without looking.

##### Study 4.

In study 4, making looking observable again increased looking. Democrats were significantly more likely to look in Both Observable (49%) than Punishment Observable (41%), *b* = 0.08 [0.02, 0.13], *t* = 2.72, *P* = 0.007, *n* = 1,284. Similarly, Republicans were more likely to look in Both Observable (28%) than Punishment Observable (15%), *b* = 0.13 [0.07, 0.19], *t* = 4.52, *P* < 0.001, *n* = 779.

Furthermore, making looking observable still did not encourage punishment without looking. Among Democrats, we find no significant difference between rates of punishment without looking in Both Observable (13%) and Punishment Observable (16%), *b* = −0.03 [−0.07, 0.01], *t* = −1.41, *P* = 0.158, *n* = 1,284. Similarly, Republicans show no significant difference between Both Observable (19%) and Punishment Observable (20%), *b* = −0.01 [−0.07, 0.04], *t* = −0.45, *P* = 0.651, *n* = 779. Thus, in study 4—which featured more ideological Evaluators—making looking observable again increased looking and did not encourage punishment without looking.

Studies 3 and 4 thus provide no evidence that reputation can drive punishment without looking as a specific reputational strategy, even when the reputational audience is strongly ideological. In both studies, showcasing looking decisions *encouraged* Actors to consider opposing perspectives, and diminished or did not influence rates of punishment without looking. Interestingly, however, making looking observable had directionally smaller positive effects on looking and negative effects on punishment without looking in study 4 than in study 3. This pattern is consistent with our finding from studies 1 and 2 that more ideological Evaluators react less negatively to punishment without (vs. with) looking.

In sum, studies 3 and 4 suggest that reputation can drive punishment without looking as a byproduct of driving punishment in general, but not as a specific reputational strategy. Moreover, they suggest that ideological audiences may facilitate punishment without looking by doing more to encourage punishment in general and less to encourage looking and discourage punishment without looking.

Finally, we note that we report how making punishment observable influenced looking in *SI Appendix*, section 4.2, and how making looking observable influenced punishment in *SI Appendix*, section 4.3. These analyses were also preregistered but are not reported in the main text because they are less relevant to our key theoretical questions.

## Discussion

Across four studies (total *n* = 10,343), we have shown that reputation fuels punishment without looking—not as a specific reputational strategy but as a byproduct of encouraging punishment in general. Highlighting the general power of reputation to encourage punishment, Evaluators rewarded punishers, and Actors punished more when punishment was observable. And because some Actors who were reputationally induced to punish chose not to look, making punishment observable increased not just punishment overall but also punishment without looking specifically. Thus, reputation drove punishment without looking as a byproduct.

Yet, reputation did *not* drive punishment without looking as a specific reputational strategy. Evaluators saw punishing without looking as an especially strong signal of loyalty, suggesting that eschewing opposing perspectives can enhance a punisher’s perceived commitment to a moral cause. However, declining to look also made punishers appear less fair and competent—perhaps because people see hastily-enacted punishment as potentially unjustified and associate deliberation with competence. On net, then, Evaluators dispreferred punishers who declined to look. And correspondingly, making looking observable did not encourage Actors to punish without looking and actually increased looking overall.

Our results also highlight the robustness of Evaluators’ preferences for deliberative punishment. Despite our finding that punishment without looking can be a strong signal of loyalty, casting doubt on an Actor’s loyalty did not make Evaluators more likely to reward such punishment. In fact, when Evaluators had active basis to question an Actor’s loyalty, they did not interpret punishment without looking as a signal of loyalty in the first place. Furthermore, giving Evaluators reason to trust an Actor’s loyalty did not cause them to preferentially reward punishment without looking, either.

Thus, we find no support for the hypothesis that reputation drives people to punish specifically without looking, in order to appear especially virtuous. Rather, our results suggest that punishers who ignore conflicting viewpoints simply prefer not to look. This preference could reflect that evaluating opposing perspectives takes time and effort and can require engaging with dissimilar (and potentially disliked) others. Moreover, some people may avoid looking because they are motivated to see punishment as merited (e.g., for ideological reasons or because punishing has reputational benefits) and do not wish to have their minds changed. Discriminating between these possibilities is an interesting direction for future research.

Our findings have implications for how society might encourage more deliberative decision-making. According to the current zeitgeist, the deep human sensitivity to reputation is a problem to be managed, rather than a tool to be leveraged, when it comes to encouraging reflective punishment. Our work, however, challenges this perspective. While we do find that reputation inspires punishment that is sometimes unreflective, we also find that people correctly believe that engaging with opposing perspectives will be perceived positively by others. Thus, by giving people opportunities to broadcast their engagement with other viewpoints, it may actually be possible to leverage reputation to motivate careful deliberation.

Still, this approach might not be a silver bullet. When reputation inspires “looking,” the result may sometimes be more performative than truly reflective. Our specific methodology suggests that reputation can genuinely increase intentions to consider opposing perspectives: When looking was observable, Actors clicked more links to opposing perspective articles, despite not knowing that their link-clicking was tracked. Yet we do not know how frequently or deeply Actors actually read the linked articles. When individuals face reputational incentives both to consider opposing perspectives *and* to ultimately punish, they may sometimes engage with countervailing perspectives superficially or in motivated ways (that bias them toward punishment)—especially if their incentives to punish are stronger than their incentives to look.

Our results also highlight the potential importance of audience ideology—both for generally driving punishment and specifically driving reflexive punishment. We found that more ideological Evaluators reacted more positively to punishment in general, and less negatively to punishment without (vs. with) looking. Moreover, comparing studies 3 and 4 suggest that, in the presence of more ideological audiences, i) making punishment observable may do more to encourage punishment overall and punishment without looking, and ii) making looking observable may do less to encourage looking and discourage punishment without looking. Still, the comparison between studies 3 and 4 remains merely suggestive: We did not randomly assign Actors to study 3 vs. 4, and the studies featured different Democrat petitions. Future work should continue exploring the influence of audience ideology on punishment behavior.

Importantly, even strongly partisan Evaluators did not significantly prefer punishment without (vs. with) looking in our studies. Yet, our ideology findings, and our finding that punishment without looking does strongly signal loyalty, suggest a framework for thinking about when punishment without looking might be evaluated most positively. For example, punishment without looking might conceivably be rewarded as a specific reputation strategy by audiences that are *extremely* ideological or that prize loyalty over fairness and competence (e.g., extremist groups)—or in situations that encourage even relatively moderate audiences to value loyalty more strongly.

Future research should evaluate the generalizability of our results across populations (e.g., representative samples of Americans, or samples from other cultures), moral domains (given that our studies focused on just a few petitions), and social environments (including environments beyond opt-in online participant pools; an investigation of punishment without looking on social media platforms would be of particular interest). It would also be valuable to consider different operationalizations of punishment. For example, punishment without looking might be perceived more negatively in the context of severe punishments (e.g., physical aggression, firing somebody) versus milder punishments (e.g., condemnatory gossip in a private, dyadic conversation). Moreover, while we investigated punitive acts that were collective and indirect (i.e., signing petitions that, with enough support, could trigger outcomes that are costly to targets), it would be interesting to consider the reputation consequences of punishment without looking when punishers act independently and directly [e.g., by personally removing resources from targets—the paradigmatic operationalization of punishment in economic game experiments involving social dilemmas (16)].

Similarly, there are many ways that somebody might look (i.e., gather relevant information) before deciding whether to punish. Future studies could investigate other looking behaviors, including gathering or verifying facts, or evaluating perspectives that are not countervailing (i.e., seeking confirmatory evidence that punishment is merited). Of note, relative to these other looking behaviors, considering opposing perspectives might appear particularly disloyal. It is perhaps especially striking, then, that looking was still rewarded in our studies. However, the amount of looking might matter too: If an individual considers *many* opposing perspectives, or demands a *huge* amount of evidence in favor of punishment, their looking might become a net reputational negative.

In conclusion, people sometimes punish alleged wrongdoers without first considering opposing perspectives. We find that reputation can fuel such “punishment without looking” by incentivizing punishment in general. Yet, we find no evidence that people use unquestioning punishment as a specific reputational strategy and show that engaging with opposing viewpoints can actually be socially rewarded.

## Materials and Methods

For all studies, full procedures and stimuli are documented in *SI Appendix*, sections 1 and 6, and data, scripts, materials, and preregistrations are online (https://osf.io/3es2k/). All studies were approved by the Northwestern University IRB, and began with subjects providing informed consent. Subjects also completed attention checks (collected prior to random assignment) and comprehension questions about study tasks. Our main text analyses restrict to subjects who passed the attention checks; see *SI Appendix*, sections 2.6 and 4.4 for analyses that additionally restrict to subjects who passed comprehension questions (and produce very similar results).

For our Evaluator studies (i.e., studies 1 and 2), we recruited a small number of Actors who were not subjects in any of our studies (although they did provide informed consent) but were recruited so that we could describe real Actors to Evaluators, and pay study bonuses. We matched each featured Actor with multiple Evaluators, although Evaluators did not learn this. Likewise, for our Actor studies (i.e., studies 3 and 4), we recruited a small number of Evaluators (who provided informed consent) to match with Actors. See *SI Appendix*, section 1.4 for more details about the matching procedures for each study.

### Studies 1a–b.

In May to June 2021, we recruited a target of *n* = 600 Evaluators from Prolific for each of studies 1a (final *n* = 629 Democrats, 58% female, 39% male, and 3% other genders; average age = 34 y) and 1b (final *n* = 600 Republicans, 55% female and 45% male; average age = 37 y). In each study, we i) introduced the Dictator Game, ii) described the Actor’s punishment and looking opportunities, iii) measured evaluations of Actors, and iv) presented demographic and other questions (including a binary strength of partisanship measure, used in our moderation analyses).

In stage (iii), subjects first evaluated Actors who did vs. did not look, before punishing. Next, in corresponding order, subjects evaluated Actors who did vs. did not look, before *not* punishing. Subjects then evaluated Actors who did vs. did not punish (without looking information). Finally, subjects evaluated an Actor about whom they had no information. For each Actor, subjects i) decided what to share if matched with the Actor (between 0 and 50¢ in 5-cent increments; we analyze the percentage of 50¢ shared), ii) rated the Actor on overall positivity, and then iii) in random order, rated the Actor on loyalty, competence, and fairness.

For all studies, we adhered closely to our preregistered analysis plans with some minor deviations. Of particular note, in study 1, we preregistered money shared as our primary DV, with analyses of overall positivity ratings as secondary. After conducting study 1, however, we concluded, based on these variables’ distributions, that positivity ratings were a more informative global evaluation measure: Whereas many Evaluators shared none or exactly half of their endowment, and almost no Evaluators shared more than half, positivity ratings were more continuously distributed. We thus preregistered positivity as our primary DV in study 2. For both studies 1 and 2, we report analyses of both global evaluation DVs but plot positivity in our main text figures; we reproduce these figures for our sharing DV in *SI Appendix*. For a complete discussion of all preregistration deviations, see *SI Appendix*, section 5.

### Studies 2a–b.

In October 2022, we recruited a target of i) *n* = 1,800 Evaluators for study 2a (final *n* = 1,796 Democrats, 58% female, 40% male, and 2% other genders; average age = 37 y), and ii) *n* = 600 Evaluators for study 2b (final *n* = 595 Democrats, 49% female, 47% male, and 4% other genders; average age = 38 y), from Prolific. Study 2, which was conducted chronologically last, did not include Republicans; we did not believe that enough Republicans on Prolific remained naive to our paradigm to afford sufficient statistical power. The method for study 2 closely mirrored that of study 1a, except that we gave subjects different background information about the Actor they were matched with (which varied across conditions, as described in the main text).

### Studies 3a–b.

In January to March 2021, we recruited a target of *n* = 1,800 Democrat Actors from MTurk for study 3a (final *n* = 1,808, 61% female and 39% male; average age = 38 y). For study 3b, we recruited a target *n* = 1,800 Republican Actors, first from MTurk and then from Prolific, once it became clear that an insufficient number of Republicans on MTurk remained naive to our paradigm (final *n* = 1,796, 52% from MTurk and 48% from Prolific, 54% female, 45% male, and <1% other genders; average age = 40 y). The flow of study 3 mirrored those of studies 1 and 2, except that subjects *themselves* had the chance to punish and look, and we manipulated what they were told that the Evaluator would learn about their behavior.

To measure looking, we presented a “looking screen” with headlines and links for two opposing perspective articles and an invitation to search the Internet for other opposing perspectives. We tracked subjects’ link-clicking behavior and define “looking” as clicking at least one link to an opposing perspective article. Subjects in Both Observable were told that the Evaluator would learn how long they spent on the looking screen, but not that we would track their link-clicking behavior. See *SI Appendix*, section 4.5 for preregistered secondary analyses that define looking as either i) time spent on the looking screen or ii) the continuous number of links clicked (and produce very similar results).

To measure punishment, we presented a link to the petition, and tracked whether subjects clicked it (again, without informing subjects of this tracking). We also asked subjects whether they signed the petition. To discourage false reporting, we asked subjects, if they signed, to report information about the screen they saw after signing. We define “punishing” as clicking the link to the petition and self-reporting signing.

### Studies 4a–b.

In October to November 2020, we conducted studies 4a–b. For each study, we preregistered initial targets of *n* = 1,200 Actors from MTurk (less than we targeted in studies 3a–b because, in study 3 but not study 4, we hypothesized that making looking observable might *decrease* punishment without looking—but expected this effect to be small, and thus to require a larger sample to detect).

For study 4a (which recruited Democrats), upon reaching our initial target of *n* = 1,200, our key results were all significant. However, while finishing data collection for study 4b (which recruited Republicans, and took longer to complete), we decided to direct Democrats to study 4a, increasing its sample size. We thus amended our study 4a preregistration, increasing our target to *n* = 2,000 subjects and registering a plan to correct, in our analyses, for “peeking” at the first batch of data before collecting the second batch. All significant results survive these corrections (*SI Appendix*, section 4.1). Our final sample sizes were *n* = 1,974 for study 4a (54% female, 45% male, and 1% other genders; average age = 40 y) and *n* = 1,145 for study 3b (56% female, 44% male, and <1% other genders; average age = 43 y).

The study 4 method was otherwise identical to the study 3 method, except that i) subjects were matched with more ideological Evaluators and ii) study 4a featured the Negy petition (whereas study 3a featured the Moore petition).

## Supplementary Material

Appendix 01 (PDF)

## Data Availability

Anonymized experimental data have been deposited in OSF (https://osf.io/3es2k/) (43).
