# Supplementary material for: How reputation does (and does not) drive people to punish without looking
Source: Proc Natl Acad Sci U S A. 2023 Jul 5;120(28):e2302475120. doi: 10.1073/pnas.2302475120 (PMC10334795; doi:10.1073/pnas.2302475120)
Supplement: Supplementary file 1 — Appendix 01 (PDF) [file pnas.2302475120.sapp.pdf]

Supplementary Information
for
How reputation does (and does not) drive people to punish without looking

1. Full study procedures ... 3
1.1 Studies 1a-b ... 3
1.2 Studies 2a-b ... 4
1.3 Studies 3-4 ... 4
1.4 Matching Actors and Evaluators ... 5
2. Supplemental analyses of Studies 1a-b ... 6
2.1 Analyses of results for the Negy vs. Moore petitions in Study 1a ... 6
2.2 Reproducing main text Figure 1 with sharing ... 7
2.3 Mediation analyses ... 7
2.4 Between-subjects analyses ... 8
2.5 Analyses of evaluations of *non-punishers* who declined vs. chose to look ... 9
2.6 Analyses of perfect comprehenders ... 9
2.6.1 Evaluations of punishers vs. non punishers ... 10
2.6.2 Evaluations of punishers who declined vs. chose to look ... 10
3. Supplemental analyses of Studies 2a-b ... 10
3.1 Reproducing main text Figure 2 with sharing ... 10
3.2 Reproducing main text Table 2 with 95% CIs ... 11
3.3 Study 2 analyses for *all* DVs ... 12
3.3.1 Ratings of Actors, when given no information about punishment or looking ... 12
3.3.2 Ratings of Actors who did vs. did not punish ... 13
3.3.3 Ratings of Actors who punished without vs. with looking ... 13
3.3.4 Ratings of Actors who did not punish without vs. with looking ... 14
3.3.5 Interpretation of analyses ... 14
3.4 Mediation analyses ... 16
3.5 Moderation analyses ... 17
4. Supplemental Analyses of Studies 3-4 ... 20
4.1 Analyses of Study 4a by batch, and corrections for "peeking" ... 20
4.2 Analyses of how making punishment observable influences looking ... 20
4.3 Analyses of how making looking observable influences punishment ... 21
4.4 Analyses of perfect comprehenders ... 21

4.4.1 Effects of making punishment observable 22
4.4.2 Effects of making looking observable 22
4.5 Analyses of alternative specifications of looking 23
4.5.1 Looking time (natural-log transformed seconds) 23
4.5.2 Number of articles 24
5. Discussion of preregistration deviations 24
5.1 Studies 1-2 24
5.3 Studies 3-4 25
6. Appendix: Experimental stimuli 26
6.1 Studies 1a-b 26
6.2 Studies 2a-b 40
6.3 Studies 3-4 46

1. Full study procedures

Below, we provide a narrative description of the procedure for each study. In Section 6 of this SI, we show the complete stimuli from each study; this section thus provides the specific wording we used to explain all study tasks and measure all dependent variables.

We also note that, per our pre-registrations, for all studies, (i) if we got duplicate responses from the same IP address or participant ID, we included only the chronologically first response in our analyses, and (ii) we excluded responses from subjects who failed at least one of two attention check questions at the beginning of the study, and/or did not complete all required measures before the demographic section at the end of the study.

1.1 Studies 1a-b

As in all studies in this paper, subjects in Studies 1a-b began by reporting their gender and political party, and then completing two attention checks. Next, we introduced subjects to the Dictator Game involving the Actor. We told subjects that they (i.e., the Evaluator) would be "Player 1" and another subject (i.e., the Actor), of their same political affiliation, would be "Player 2". Furthermore, subjects learned that they would receive 50 cents, and decide how much to share with Player 2. Subjects then answered three comprehension questions about the Dictator Game.

Next, we introduced subjects to the Actor's punishment and looking decisions. We told subjects that Player 2 had the chance to sign the relevant punitive petition, and provided subjects with a brief description, and screenshot, of the petition. Next, we told subjects that before deciding whether to sign, Player 2 could read opposing perspective articles and/or search the Internet for other opposing perspectives. In particular, we told subjects that Player 2 received links to articles providing opposing perspectives, and provided a screenshot of an example headline, alongside a brief explanation of how it constituted an opposing perspective. We also told subjects that Player 2s varied in the time they spent considering opposing perspectives.

Next, we collected subjects' evaluations of different Actors. First, we asked subjects to consider a Player 2 who *did* sign the petition, after considering opposing perspectives for an above- or below-average amount of time (manipulated between-subjects). Thus, we described a punisher who did, or did not, look. We told subjects that they might be matched with this Player 2, and then measured the amount of money they wished to share with Player 2 in this event (between 0 and 50 cents in 5-cent increments). Afterwards, subjects rated the Player 2 on overall positivity, and then, in random order, loyalty towards Black Lives Matter or Blue Lives Matter, competence, and fairness (on 0-to-100 scales).

Next, we told subjects that they would make sharing decisions about other Player 2s they might be matched with. Subjects then evaluated six other Player 2s, with six other behavioral profiles. The second Player 2 was a punisher who did or did not look (whichever the subject did *not* evaluate first). Next, subjects evaluated *non*-punishers who did, or did not, look (in an order corresponding to the order in which they evaluated punishers). Then, Subjects evaluated non-punishers and punishers, without receiving information about their looking (in random order). Finally, subjects evaluated a Player 2 without receiving any information about their decisions.

Afterwards, we presented subjects with a post-experimental survey in which they evaluated the petition, described how they made their decisions, shared their impressions of the study, rated their previous participation in related studies and beliefs about whether the petition and Player 2 were real, and answered demographic and ideology questions. In this survey, subjects answered a binary strength of partisanship measure, used in our moderation analyses (in

which they were asked to identify as either "a strong Democrat/Republican, who strongly supports the party" or "a weak Democrat/Republican, who only leans towards the party").

1.2 Studies 2a-b

The method for Study 2 closely mirrored Study 1a, with a few differences. In Study 1, subjects evaluated multiple distinct Player 2s, who they learned that they might be matched with, and who behaved in different ways. In contrast, subjects in Study 2 evaluated just one Player 2, who they learned that they were matched with, in the event that this Player 2 behaved in different ways. This allowed us to, in our Study 2 treatments that added information, provide subjects with specific background information about the Player 2 they were paired with. Furthermore, to encourage subjects to reflect on this background information, we asked subjects across all conditions of Study 2 to write a paragraph about their initial impression of Player 2, before they evaluated different potential Player 2 behaviors.

In the "baseline" conditions of Studies 2a-b, the method was otherwise identical to the Study 1a method. In the "other person" treatment of Study 2a, when Player 2 was introduced, Player 2 was characterized as "another participant" and no information was provided about Player 2's political affiliation. In all other treatments of Studies 2a-b, Player 2 was initially described as a co-partisan (like in Study 1a and the baseline conditions). Then, after subjects learned about Player 2's punishment and looking opportunities, they were shown a screenshot of some questions that Player 2 was asked, and Player 2's responses. These screenshots varied across treatments, and were shown on the page where subjects described their initial impression of Player 2. In the main text, we overview the content of these screenshots in each treatment; in Section 6.2 of this SI, we reproduce each screenshot.

1.3 Studies 3-4

After reporting their gender and political party, and completing two attention checks, subjects in Studies 3-4 learned about the Dictator Game. We told subjects that they (i.e., the Actor) would be Player 2 and another subject (i.e., the Evaluator) of their same political affiliation would be Player 1, receive 50 cents, and decide how much to share with them. We described the Player 1 as "a weak [Democrat/Republican], who only leans towards the party" in Study 3, and "a strong [Democrat/Republican] who strongly supports [Black Lives Matter/Blue Lives Matter]" in Study 4. Subjects then answered three comprehension questions about the Dictator Game (and were required to correct any incorrect answers before preceding).

Next, we introduced subjects to their punishment and looking decisions. We presented subjects with a brief description, and screenshot, of the relevant petition, explaining that they would later have the chance to sign it. Subjects in *Nothing Observable* learned that Player 1 would *not* find out whether they signed; subjects in *Punishment Observable* and *Both Observable* learned that Player 1 *would* find this out.

Afterwards, subjects learned that they could consider opposing perspectives before deciding whether to sign. We told subjects that we would provide links to opposing perspective articles (and provided a screenshot of an example headline) and that they could also search the Internet for other opposing perspectives. Subjects in *Nothing Observable* and *Punishment Observable* learned that Player 1 would *not* find out how long they spent considering opposing perspectives; subjects in *Both Observable* learned that Player 1 *would* find this out. Subjects then answered three comprehension questions about what Player 1 would learn about their behavior (and again were required to correct any incorrect answers).

We then told subjects they would next get to consider opposing perspectives, and reminded them about whether their time spent doing so would be observable. Then, subjects advanced to a screen in which we provided headlines for and links to two opposing perspective articles, and reminded subjects than they could search for other articles. We told subjects to advance the screen once they were ready to decide whether to sign the petition. On this “looking” screen, we tracked subjects’ link-clicking behavior and, per our preregistration, defined “looking” as clicking at least one link to an opposing perspective article. We did not tell subjects that their link-clicking would be tracked.

We then told subjects that they would next decide whether to sign the petition, and that if they reported signing, we would ask them to show us that they signed by reporting information about the screen that Change.org displayed after they signed. (We also assured subjects that we would *not* collect their identifying information if they signed). Next, we gave subjects a link to the petition and asked them whether they signed (and prompted them to report information about the completion screen if so). We also asked subjects about their level of commitment to supporting the petition; per our preregistrations, we do not analyze this DV.

On this “petition” screen, we tracked subjects’ link-clicking behavior and, per our preregistration, defined “signing” (i.e., punishing) as clicking the link to the petition and self-reporting signing. We did not tell subjects that their link-clicking would be tracked. We did not incorporate the reported completion screen information into our pre-registered definition of “signing”, because (i) pilot testing revealed that almost everyone who met the above criteria *did* report this information correctly, but (ii) there is some room for ambiguity in categorizing reports as “correct” (given that some responses are close to but not completely accurate).

Afterwards, we presented subjects with a post-experimental survey in which they evaluated the petition, described how they spent their time on the “looking” screen and made their decisions, rated their previous familiarity with the petition, the extent to which they were motivated by reputation, and their beliefs about whether the petition, articles, and Player 1 were real, and answered demographic and ideology questions.

1.4 Matching Actors and Evaluators

In Studies 1-2, focal subjects were Evaluators. Each Evaluator was matched with a real Actor. To pay each Evaluator’s bonus, we implemented one of their sharing decisions, corresponding to how their Actor actually behaved.

The Actors that Evaluators in Studies 1-2 were matched with were not themselves subjects in any of our studies (i.e., we do not report analyses of their behavior, although they did provide informed consent to participate). Rather, we recruited a small number of Actors, with the specific aim of matching them with Evaluators (so that we could describe real Actors to Evaluators, and pay study bonuses). We matched each featured Actor with multiple Evaluators, although Evaluators did not learn this.

When recruiting Actors to match with Evaluators from Study 2, we asked a set of survey questions that we believed might elicit responses that would cast doubt on, or highlight, Actors’ loyalty. These Actors provided informed consent for their responses to potentially be shown to other online participants (who they learned might view their responses, evaluate them, and decide how much money to share with them).

In Study 1, we simply matched Evaluators with co-partisan Actors. And in the “baseline” and “other participant” conditions of Study 2, we likewise matched Evaluators (who were all Democrats) with Democrat Actors (although Evaluators in the “other participant” condition did

not learn that their Actor was a Democrat). In the remaining conditions of Study 2, we matched Evaluators with specific Democrat Actors who we chose to feature, on the basis of their responses to the above-described questions (selecting responses that cast *doubt* on the Actor's loyalty in the relevant conditions of Study 2a, and selecting a response that *highlighted* the Actor's loyalty in the relevant condition of Study 2b). Each of these conditions featured a different Actor, and we showed Evaluators in these conditions a screenshot of their Actor's survey responses.

In Studies 3-4, focal subjects were Actors. Each Actor was matched with a real Evaluator. To pay each Actor's bonus, we implemented one of their Evaluator's sharing decisions, corresponding to the Actor's observability condition and how the Actor actually behaved (e.g., if Actor was in the "Punishment Only" condition and chose to punish, we paid the Actor the amount that their Evaluator chose to share with a punisher, given no information about looking).

Mirroring Studies 1-2, the Evaluators that Actors in Studies 3-4 were matched with were not themselves subjects in any of our studies (although they did provide informed consent to participate). Rather, we recruited a small number of Evaluators specifically to match with Actors (and matched each featured Evaluator with multiple Actors, without Actors knowing this). In Study 3, we matched Actors with co-partisan Evaluators who reported identifying as *weak* partisans, and in Study 4, we matched Actors with co-partisan Evaluators who reported identifying as *strong* partisans, and also reported strongly supporting Black/Blue Lives Matter.

2. Supplemental analyses of Studies 1a-b

2.1 Analyses of results for the Negy vs. Moore petitions in Study 1a

In Study 1a, subjects were randomly paired with Actors who either had the chance to sign the Negy (n = 308) or Moore (n = 321) petition. As reported in the main text, the Negy and Moore petitions produced identical patterns of results; thus, our main text results collapsed over petition. However, per our pre-registration, in Table S1 below, we report our key Study 1 analyses separately for each petition, and also report interaction results that compare results across the two petitions. We find that each of our key results hold significantly within each of the two petitions. However, for some results, the effect size differs significantly across petitions, as reflected by a significant interaction.

	**Evaluations of punishers vs. non-punishers**<br>(Positive coefficients reflect preferences for *punishment*)	**Evaluations of punishment without vs. with looking**<br>(Positive coefficeints reflect preferences for punishment *without* looking)
**Positivity**	Negy Petition: b = 26.81 [23.17, 30.45], t = 14.51, p < .001<br>Moore Petition: b = 21.45 [17.93, 24.98], t = 11.99, p < .001<br>Interaction: b = -5.36 [-10.41, -0.31], t = -2.08, p = .038	Negy Petition: b = -8.56 [-11.25, -5.87], t = -6.26, p < .001<br>Moore Petition: b = -12.29 [-14.85, -9.72], t = -9.44, p < .001<br>Interaction: b = -3.73 [-7.44, -0.03], t = -1.98, p = .048
**Sharing**	Negy Petition: b = 17.99 [14.96, 21.02], t = 11.68, p < .001<br>Moore Petition: b = 13.71 [11.05, 16.36], t = 10.17, p < .001<br>Interaction: b = -4.28 [-8.30, -0.26], t = -2.09, p = .037	Negy Petition: b = -3.70 [-5.98, -1.42], t = -3.19, p = .002<br>Moore Petition: b = -5.05 [-6.85, -3.24], t = -5.49, p < .001<br>Interaction: b = -1.35 [-4.25, 1.56], t = -0.91, p = .363
**Fair**	Negy Petition: b = 21.60 [18.39, 24.82], t = 13.22, p < .001<br>Moore Petition: b = 16.96 [14.02, 19.89], t = 11.38, p < .001<br>Interaction: b = -4.65 [-8.99, -0.31], t = -2.10, p = .036	Negy Petition: b = -15.73 [-18.50, -12.97], t = -11.19, p < .001<br>Moore Petition: b = -16.84 [-19.87, -13.81], t = -10.94, p < .001<br>Interaction: b = -1.11 [-5.20, 2.98], t = -0.53, p = .594
**Competent**	Negy Petition: b = 19.62 [16.64, 22.60], t = 12.95, p < .001<br>Moore Petition: b = 15.30 [12.49, 18.11], t = 10.71, p < .001<br>Interaction: b = -4.31 [-8.40, -0.22], t = -2.07, p = .039	Negy Petition: b = -10.18 [-12.74, -7.62], t = -7.82, p < .001<br>Moore Petition: b = -11.99 [-14.28, -9.69], t = -10.27, p < .001<br>Interaction: b = -1.81 [-5.24, 1.62], t = -1.04, p = .300
**Loyal**	Negy Petition: b = 36.39 [33.11, 39.66], t = 21.85, p < .001<br>Moore Petition: b = 37.24 [34.19, 40.28], t = 24.05, p < .001<br>Interaction: b = 0.85 [-3.61, 5.31], t = 0.37, p = .708	Negy Petition: b = 4.69 [2.37, 7.00], t = 3.99, p < .001<br>Moore Petition: b = 6.37 [4.16, 8.59], t = 5.66, p < .001<br>Interaction: b = 1.69 [-1.51, 4.88], t = 1.04, p = .300

Table S1. Results of Study 1a by petition.

2.2 Reproducing main text Figure 1 with sharing

Below, in Figure S1, we reproduce main text Figure 1 (which plotted positivity ratings) with our sharing DV. We find qualitatively identical patterns, with one exception. For positivity, strong partisan Democrats showed a significant preference for punishment *with* (vs. without) looking; for sharing, strong partisan Democrats show no significant difference between punishment without vs. with looking.

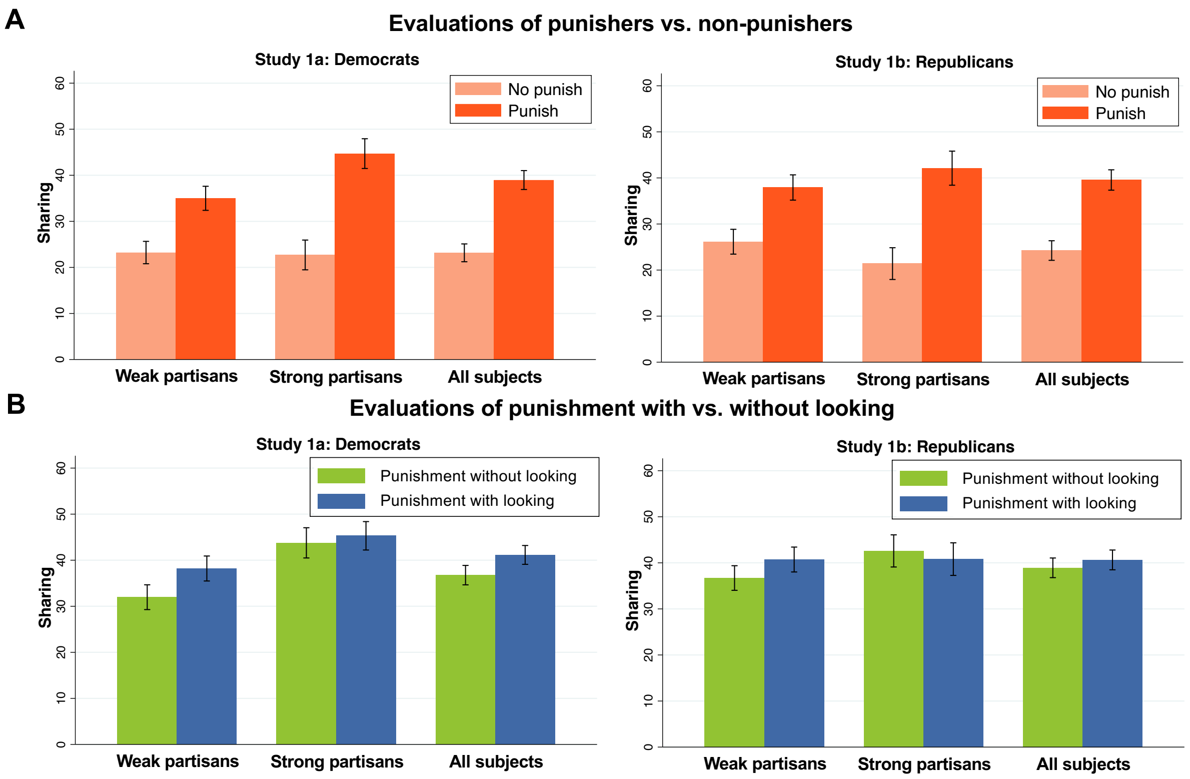

Figure S1. Reproducing main text Figure 1, but with our sharing DV.

2.3 Mediation analyses

As described in the main text, Evaluators in Studies 1a-b rated punishers who declined (vs. chose) to consider opposing perspectives less positively overall, and also rated them as less fair and less competent. Interestingly, however, they rated punishers who declined to look as *more* loyal. This pattern of results suggests that (i) declining to look has reputational costs and advantages, and (ii) the advantages are outweighed by the costs, such that declining to look is a net reputational negative. Here, we support this proposal with mediation analyses.

Before describing our mediation analyses, we note that two of our mediators, fairness and competence, were highly correlated with each other (among Democrats, $r = .86$, $p < .001$; among Republicans, $r = .87$, $p < .001$). In contrast, loyalty was less strongly correlated with the other mediators (correlations with fairness: Democrats, $r = .63$, $p < .001$; Republicans, $r = .51$, $p < .001$; correlations with competence: Democrats, $r = .59$, $p < .001$; Republicans, $r = .49$, $p < .001$).

In light of this correlation structure, we consider two distinct multiple mediation models: one that takes fairness and loyalty as mediators, and one that takes competence and loyalty as mediators. This approach avoids placing fairness and competence together in a single model,

which would be uninformative in light of their high collinearity. In each model, we take overall positivity as our dependent variable, the contrast between punishers who declined vs. chose to look as our independent variable, and the relevant pair of mediators as our mediating variables.

In Table S2, for each of Studies 1a-b, we report the total effect of not looking on overall positivity, as well as analyses from each of our two mediation models. In particular, for each mediator within each model, we report (i) the A path (i.e., the effect of not looking on the mediator), (ii) the B path (i.e., the effect of the mediator on overall positivity, controlling for not looking and the other mediator), and (iii) the indirect effect of not looking on overall positivity via the mediator. Additionally, for each model, we report the direct effect of not looking on overall positivity.

For each of Studies 1a-b, we consistently observe significant *negative* indirect effects for fairness and competence, and significant *positive* indirect effects for loyalty. Furthermore, the positive indirect effects of loyalty are consistently significantly smaller in magnitude than the negative indirect effects of fairness and competence (as revealed by the 95% CIs reported in Table S1), consistent with the negative total effect of not looking on overall positivity.

		**Democrats (Study 1a)** ***n*** **= 629**	**Republicans (Study 1b)** ***n*** **= 600**
Total Effect		*b* = -10.46 [-12.32, -8.60], *t* = -11.07, *p* < .001	*b* = -4.55 [-6.30, -2.79], *t* = -5.08, *p* < .001
Model with Fair & Loyal as mediators	Effects for Fair	Path A: *b* = -16.30 [-18.35, -14.25], *z* = -15.62, *p* < .001	Path A: *b* = -8.62 [-10.37, -6.86], *z* = -9.63, *p* < .001
		Path B: *b* = .75 [.70, .81], *z* = 27.59, *p* < .001	Path B: *b* = .78 [.74, .82], *z* = 36.77, *p* < .001
		Indirect effect: *b* = -12.27 [-14.05, -10.50]	Indirect effect: *b* = -6.74 [-8.16, -5.32]
	Effects for Loyal	Path A: *b* = 5.55 [3.95, 7.14], *z* = 6.83, *p* < .001	Path A: *b* = 6.90 [4.92, 8.88], *z* = 6.83, *p* < .001
		Path B: *b* = .12 [.06, .17], *z* = 4.36, *p* < .001	Path B: *b* = .08 [.04, .12], *z* = 4.35, *p* < .001
		Indirect effect: *b* = .65 [.30, 1.00]	Indirect effect: *b* = .56 [.26, .86]
	Direct effect	*b* = 1.16 [-.35, 2.67], *z* =1.51, *p* = .132	*b* = 1.63 [.28, 2.98], *z* = 2.37, *p* = .018
Model with Competent & Loyal as mediators	Effects for Competent	Path A: *b* = -11.10 [-12.81, -9.39], *z* = -12.74, *p* < .001	Path A: *b* = -5.20 [-6.86, -3.53], *z* = -6.10, *p* < .001
		Path B: *b* = .78 [.73, .83], *z* = 30.40, *p* < .001	Path B: *b* = .78 [.73, .84], *z* = 28.86, *p* < .001
		Indirect effect: *b* = -8.63 [-10.07, -7.19]	Indirect effect: *b* = -4.07 [-5.41, -2.73]
	Effects for Loyal	Path A: *b* = 5.55 [3.95, 7.14], *z* = 6.83, *p* < .001	Path A: *b* = 6.90 [4.92, 8.88], *z* = 6.83, *p* < .001
		Path B: *b* = .13 [.07, .18], *z* = 4.43, *p* < .001	Path B: *b* = .06 [.02, .10], *z* = 3.18, *p* = .001
		Indirect effect: *b* = .70 [.33, 1.07]	Indirect effect: *b* = .41 [.13, .70]
	Direct effect	*b* = -2.53 [-3.91, -1.15], *z* = -3.58, *p* < .001	*b* = -.89 [-2.19, .41], *z* = -1.34, *p* = .180

Table S2. Mediation analyses of Studies 1a-b.

2.4 Between-subjects analyses

As described in the main text, the designs of Studies 1a-b allow us to examine Evaluators' responses to punishers who declined vs. chose to look in a between-subjects analysis (because Evaluators encountered these two Actor profiles first, and we randomized their order).

These between-subject analyses were also pre-registered, and produce results that are similar in direction and magnitude to the within-subject analyses we report in our main text. However, in line with the reduced statistical power they afford, they are less consistently significant. Below, we report the between-subject results for each of our dependent variables.

Among Democrats (n = 629), in our between-subjects analyses, Evaluators formed more negative overall impressions of punishers who declined (vs. chose) to look (b = -9.92 [-13.35, -6.49], t = -5.68, p < .001). They also shared directionally less money with punishers who declined to look, although this difference was not significant (b = -3.44 [-7.65, .77], t = -1.60, p = .109). And they rated punishers who declined to look as significantly less fair (b = -15.83 [-19.38, -12.28], t = -8.76, p < .001) and less competent (b = -10.32 [-13.63, -7.01], t = -6.12, p < .001). In contrast, however, they rated punishers who declined to look as more loyal supporters of Black Lives Matter (b = 7.44 [4.32, 10.56], t = 4.68, p < .001).

Among Republicans (n = 600), our between-subjects analyses reveal no significant differences between evaluations of punishers who declined (vs. chose) to look with respect to overall positivity ratings (b = -2.08 [-5.84, 1.67], t = -1.09, p = .276), money shared (b = -2.49 [-6.80, 1.82], t = -1.14, p = .257) or ratings of competence (b = -1.76 [-5.33, 1.82], t = -0.96, p = .335). Punishers who declined to look were, however, rated as significantly less fair (b = -4.32 [-7.94, -.70], t = -2.34, p = .020) and significantly more loyal supporters of Blue Lives Matter (b = 10.83 [5.99, 15.66], t = 4.40, p < .001).

2.5 Analyses of evaluations of *non-punishers* who declined vs. chose to look

Our main text focuses on how Evaluators in Studies 1a-b evaluated *punishers* who declined vs. chose to look. However, as described in the main text, the designs of Studies 1a-b also allow us to investigate evaluations of *non-punishers* who declined vs. chose to look. Here, we report analyses of these evaluations.

Among Democrats (n = 629), we find that Evaluators formed less positive overall impressions of non-punishers who declined (vs. chose) to look (b = -13.31 [-14.97, -11.65], t = -15.78, p < .001), shared less money with them (b = -7.74 [-9.12, -6.36], t = -11.02, p < .001), and rated them as significantly less fair (b = -17.66 [-19.53, -15.78], t = -18.52, p < .001), less competent (b = -14.38 [-16.14, -12.63], t = -16.13, p < .001), and less loyal (b = -7.19 [-8.63, -5.75], t = -9.81, p < .001). Similarly, Republicans (n = 600) rated non-punishers who declined to look as less positive overall (b = -9.22 [-11.14, -7.30], t = -9.43, p < .001), shared less money with them (b = -4.82 [-6.29, -3.34], t = -6.40, p < .001), and rated them as well as less fair (b = -12.59 [-14.69, -10.48], t = -11.74, p < .001), less competent (b = -10.14 [-12.05, -8.23], t = -10.44, p < .001), and marginally significantly less loyal (b = -1.95 [-4.04, .14], t = -1.83, p = .068).

These analyses demonstrate that Evaluators created reputational incentives for Actors to consider opposing perspectives, even if they did not ultimately choose to punish. In this way, they serve to bolster our conclusion that looking can have positive reputational consequences.

2.6 Analyses of perfect comprehenders

As reported in the main text, our main text analyses include all subjects who passed two attention checks, regardless of performance on comprehension questions. However, per our pre-registration, here we report secondary analyses that restrict to subjects who correctly answered all comprehension questions (and produce very similar results).

2.6.1 Evaluations of punishers vs. non punishers

We begin by reporting analyses of how Evaluators who showed perfect comprehension reacted to Actors who did vs. did not punish, when given no information about whether the Actor chose to look.

Among Democrats (n = 533), Evaluators formed more positive overall impressions of punishers than non-punishers (b = 23.97 [21.25, 26.69], t = 17.31, p < .001) and also shared significantly more money with punishers (b = 15.42 [13.25, 17.59], t = 13.95, p < .001). Furthermore, subjects rated punishers as more loyal (b = 37.80 [35.40, 40.19], t = 30.96, p < .001), fair (b = 18.92 [16.55, 21.29], t = 15.69, p < .001), and competent (b = 17.32 [15.10, 19.54], t = 15.34, p < .001). Similarly, Republicans (n = 494) evaluated punishers more positively overall (b = 22.38 [18.65, 26.12], t = 11.79, p < .001), shared more money with punishers (b = 14.29 [11.61,16.98], t = 10.46, p < .001), and rated punishers as more loyal (b = 28.05 [24.24, 31.87], t = 14.44, p < .001), fair (b = 19.87 [16.86, 22.88], t = 12.97, p < .001), and competent (b = 18.09 [15.23, 20.95], t = 12.45, p < .001).

2.6.2 Evaluations of punishers who declined vs. chose to look

Next, we report analyses of how Evaluators who showed perfect comprehension reacted to Actors who chose to punish, after declining vs. choosing to consider opposing perspectives.

Among Democrats (n = 533), we find that Evaluators formed more negative overall impressions of punishers who declined (vs. chose) to look (b = -10.75 [-12.75, -8.75], t = -10.55, p < .001). They also shared less money with punishers who declined to look (b = -4.60 [-6.09, -3.11], t = -6.06, p < .001), and rated them as less fair (b = -16.71 [-18.95, -14.48], t = -14.69, p < .001) and less competent (b = -11.59 [-13.46, -9.72], t = -12.16, p < .001), but more loyal (b = 6.15 [4.42, 7.89], t = 6.98, p < .001). Similarly, Republicans (n = 494) rated punishers who declined to look less positively overall (b = -4.34 [-6.32, -2.36], t = -4.30, p < .001), shared directionally, but not significantly, less money with them (b = -1.21 [-2.82, .39], t = -1.48, p = .138) and rated them as less fair (b = -9.04 [-10.99, -7.08], t = -9.10, p < .001) and less competent (b = -4.93 [-6.75, -3.12], t = -5.33, p < .001), but more loyal (b = 7.47 [5.21, 9.72], t = 6.51, p < .001).

3. Supplemental analyses of Studies 2a-b

In this section, we report supplemental analyses of Studies 2a-b. We note that for these two studies, given the large number of conditions—and the resultingly large number of potential analyses—we chose *not* to pre-register secondary analyses that restricted to subjects who correctly answered all comprehension questions. Therefore, we do not include "perfect comprehender" analyses in this section. However, analyses that restrict to perfect comprehenders show similar patterns, reinforcing our conclusions.

3.1 Reproducing main text Figure 2 with sharing

Below, in Figure S2, we reproduce main text Figure 2 (which plotted positivity ratings) with our sharing DV; we find qualitatively identical patterns.

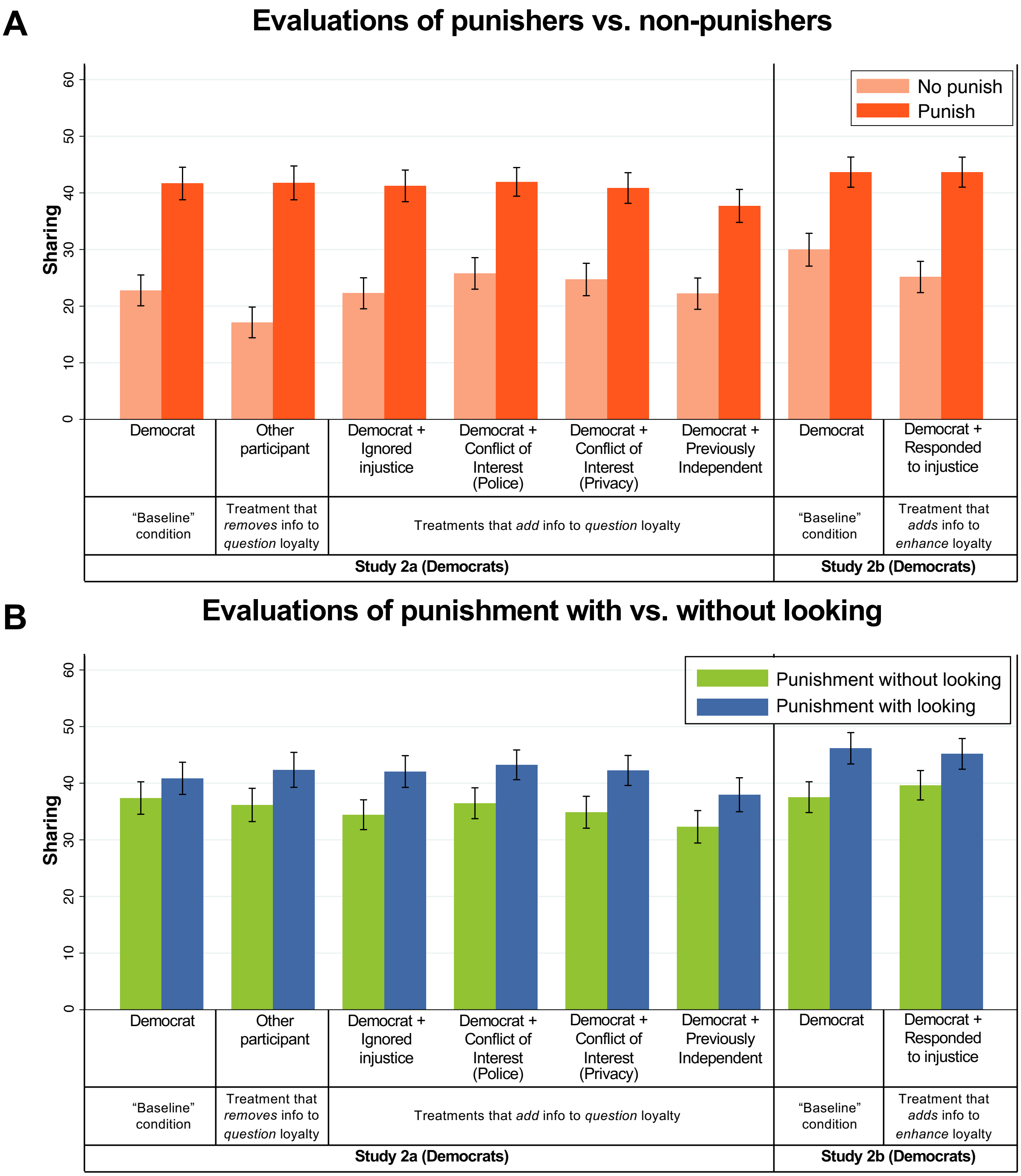

Figure S2. Reproducing main text Figure 2, but with our sharing DV.

3.2 Reproducing main text Table 2 with 95% CIs

In main text Table 2, we reported analyses, for each Study 2 condition, of Evaluators' (A) ratings of Actor loyalty, when given no information about punishment or looking, (B) global evaluations of Actors who did vs. did not punish, and (C) global evaluations of Actors who punished without vs. with looking. Below, in Table S3, we reproduce this table, but with 95% CIs on the regression coefficients.

	Study 2a (Democrats) *n* = 1796						Study 2b (Democrats) *n* = 595	
	"Baseline" condition	Treatments designed to *question* loyalty					"Baseline" condition	Treatment designed to *establish* loyalty
		Remove info	*Add* info					*Add* info
	**Democrat** *n* = 302	**Other participant** *n* = 289	**Democrat + Ignored injustice** *n* = 291	**Democrat + Conflict of Interest (Police)** *n* = 308	**Democrat + Conflict of Interest (Privacy)** n = 302	**Democrat + Previously Independent** *n* = 304	**Democrat** *n* = 292	**Democrat + Responded to Injustice** *n* = 303
	**(A) Loyalty manipulation check results (numbers reflect *baseline ratings,* given no information about punishment or looking)**							
**Loyal**	Mean = 55.10, SD = 12.88	Mean = 50.12, SD = 12.90	Mean = 54.57, SD = 15.78	Mean = 50.88, SD = 14.20	Mean = 52.93, SD = 14.02	Mean = 46.23, SD = 19.01	Mean = 54.53, SD = 12.99	Mean = 58.69, SD = 15.99
		b = -4.98 [-7.07, -2.90], *t* = -4.70, *p* < .001	*b* = -0.54 [-2.86, 1.78], *t* = -0.45, *p* = .650	*b* = -4.23 [-6.38, -2.07], *t* = -3.85, *p* < .001	*b* = -2.17 [-4.32, -0.02], *t* = -1.98, *p* = .048	*b* = -8.87 [-11.46, -6.28], *t* = -6.72, *p* < .001		*b* = 4.16 [1.81, 6.51], *t* = 3.48, *p* = .001
	**(B) Global evaluations of punishers vs. non-punishers (positive coefficients reflect preferences for *punishment*)**							
**Positivity**	*b* = 30.68 [26.84, 34.52], *t* = 15.73, *p* < .001	*b* = 39.07 [34.68, 43.46], *t* = 17.53, *p* < .001	*b* = 29.51 [25.64, 33.37], *t* = 15.03, *p* < .001	*b* = 28.80 [25.10, 32.49], *t* = 15.34, *p* < .001	*b* = 27.89 [24.37, 31.42], *t* = 15.57, *p* < .001	*b* = 26.28 [22.61, 29.95], *t* = 14.10, *p* < .001	*b* = 23.32 [19.71, 26.93], *t* = 12.72, *p* < .001	*b* = 29.37 [25.62, 33.12], *t* = 15.42, *p* < .001
		b = 8.39 [2.57, 14.20], *t* = 2.83, *p* = .005	*b* = -1.17 [-6.61, 4.26], *t* = -0.42, *p* = .672	*b* = -1.89 [-7.20, 3.43], *t* = -0.70, *p* = .486	*b* = -2.79 [-7.99, 2.41], *t* = -1.05, *p* = .293	*b* = -4.40 [-9.70, 0.90], *t* = -1.63, *p* = .103		*b* = 6.05 [0.86, 11.24], *t* = 2.29, *p* = .022
**Sharing**	*b* = 18.87 [15.82, 21.93], *t* = 12.15, *p* < .001	*b* = 24.64 [21.12, 28.15], *t* = 13.79, *p* < .001	*b* = 18.97 [15.97, 21.97], *t* = 12.45, *p* < .001	*b* = 16.17 [13.32, 19.02], *t* = 11.17, *p* < .001	*b* = 16.16 [13.32, 19.00], *t* = 11.20, *p* < .001	*b* = 15.49 [12.44, 18.54], *t* = 10.00, *p* < .001	*b* = 13.70 [10.71, 16.69], *t* = 9.02, *p* < .001	*b* = 18.51 [15.49, 21.54], *t* = 12.06, *p* < .001
		b = 5.76 [1.11, 10.41], *t* = 2.43, *p* = .015	*b* = 0.09 [-4.18, 4.37], *t* = 0.04, *p* = .965	*b* = -2.71 [-6.87, 1.46], *t* = -1.27, *p* = .203	*b* = -2.72 [-6.88, 1.45], *t* = -1.28, *p* = .201	*b* = -3.38 [-7.69, 0.93], *t* = -1.54, *p* = .124		*b* = 4.82 [0.58, 9.06], *t* = 2.23, *p* = .026
	**(C) Global evaluations of punishment without vs. with looking (positive coefficients reflect preferences for punishment *without* looking)**							
**Positivity**	*b* = -10.39 [-13.15, -7.64], *t* = -7.43, *p* < .001	*b* = -11.35 [-14.87, -7.84], *t* = -6.35, *p* < .001	*b* = -12.46 [-15.32, -9.60], *t* = -8.58, *p* < .001	*b* = -12.21 [-14.80, -9.62], *t* = -9.27, *p* < .001	*b* = -13.75 [-16.52, -10.98], *t* = -9.78, *p* < .001	*b* = -10.44 [-13.12, -7.76], *t* = -7.67, *p* < .001	*b* = -13.28 [-16.26, -10.29], *t* = -8.76, *p* < .001	*b* = -10.34 [-13.33, -7.36], *t* = -6.81, *p* < .001
		b = -0.96 [-5.41, 3.50], *t* = -0.42, *p* = .673	*b* = -2.07 [-6.02, 1.89], *t* = -1.03, *p* = .306	*b* = -1.81 [-5.59, 1.96], *t* = -0.94, *p* = .345	*b* = -3.36 [-7.25, 0.54], *t* = -1.69, *p* = .091	*b* = -0.05 [-3.88, 3.78], *t* = -0.02, *p* = .981		*b* = 2.93 [-1.28, 7.14], *t* = 1.37, *p* = .172
**Sharing**	*b* = -3.48 [-5.84, -1.11], *t* = -2.89, *p* = .004	*b* = -6.19 [-8.57, -3.81], *t* = -5.12, *p* < .001	*b* = -7.63 [-9.76, -5.49], *t* = -7.04, *p* < .001	*b* = -6.79 [-9.04, -4.53], *t* = -5.91, *p* < .001	*b* = -7.38 [-9.57, -5.19], *t* = -6.63, *p* < .001	*b* = -5.66 [-7.84, -3.48], *t* = -5.11, *p* < .001	*b* = -8.63 [-11.07, -6.19], *t* = -6.96, *p* < .001	*b* = -5.54 [-7.86, -3.23], *t* = -4.70, *p* < .001
		b = -2.72 [-6.06, 0.63], *t* = -1.59, *p* = .111	*b* = -4.15 [-7.33, -0.97], *t* = -2.57, *p* = .011	*b* = -3.31 [-6.57, -0.05], *t* = -1.99, *p* = .047	*b* = -3.91 [-7.12, -0.69], *t* = -2.39, *p* = .017	*b* = -2.18 [-5.39, 1.03], *t* = -1.33, *p* = .182		*b* = 3.09 [-0.27, 6.44], *t* = 1.81, *p* = .072

Table S3. Reproducing main text Table 2, but with 95% CIs on regression coefficients.

3.3 Study 2 analyses for *all* DVs

Next, we provide a more complete set of Study 2 analyses for *all* DVs. Across a series of tables below, we report, for each Study 2 condition, analyses of Evaluators' (i) ratings of Actors on all DVs, when given no information about punishment or looking (Table S4a), (ii) ratings of Actors who did vs. did not punish on all DVs (Table S4b), (iii) ratings of Actors who punished without vs. with looking on all DVs (Table S4c), and (iv) ratings of Actors who did not punish without vs. with looking on all DVs (Table S4d).

3.3.1 Ratings of Actors, when given no information about punishment or looking

	Study 2a						Study 2b	
	**Democrat**	**Other participant**	**Democrat + Ignored injustice**	**Democrat + Conflict of Interest (Police)**	**Democrat + Conflict of Interest (Privacy)**	**Democrat + Previously Independent**	**Democrat**	**Democrat + Responded to Injustice**
**Positivity**	Mean = 57.36, SD = 12.67	Mean = 52.62, SD = 9.87	Mean = 57.30, SD = 14.83	Mean = 55.77, SD = 13.33	Mean = 54.86, SD = 13.72	Mean = 51.93, SD = 16.08	Mean = 58.13, SD = 13.11	Mean = 58.81, SD = 13.75
		b = -4.73 [-6.58, -2.89], *t* = -5.05, *p* < .001	*b* = -0.06 [-2.28, 2.16], *t* = -0.05, *p* = .956	*b* = -1.58 [-3.65, 0.48], *t* = -1.50, *p* = .133	*b* = -2.50 [-4.61, -0.39], *t* = -2.32, *p* = .020	*b* = -5.43 [-7.74, -3.12], *t* = -4.61, *p* < .001		*b* = 0.67 [-1.49, 2.84], *t* = 0.61, *p* = .542
**Sharing**	Mean = 32.58, SD = 24.17	Mean = 28.79, SD = 24.49	Mean = 34.05, SD = 23.64	Mean = 34.74, SD = 23.89	Mean = 32.58,SD = 24.25	Mean = 30.39, SD = 23.88	Mean = 36.20, SD = 23.26	Mean = 35.84, SD = 23.10
		b = -3.79 [-7.72, 0.14], *t* = -1.90, *p* = .059	*b* = 1.47 [-2.38, 5.33], *t* = 0.75, *p* = .454	*b* = 2.16 [-1.66, 5.98], *t* = 1.11, *p* = .268	*b* = 0.00 [-3.87, 3.87], *t* = 0.00, *p* = 1.00	*b* = -2.19 [-6.02, 1.64], *t* = -1.12, *p* = .263		*b* = -0.36 [-4.09, 3.38], *t* = -0.19, *p* = .851
**Loyal**	Mean = 55.10, SD = 12.88	Mean = 50.12, SD = 12.90	Mean = 54.57, SD = 15.78	Mean = 50.88, SD = 14.20	Mean = 52.93, SD = 14.02	Mean = 46.23, SD = 19.01	Mean = 54.53, SD = 12.99	Mean = 58.69, SD = 15.99
		b = -4.98 [-7.07, -2.90], *t* = -4.70, *p* < .001	*b* = -0.54 [-2.86, 1.78], *t* = -0.45, *p* = .650	*b* = -4.23 [-6.38, -2.07], *t* = -3.85, *p* < .001	*b* = -2.17 [-4.32, -0.02], *t* = -1.98, *p* = .048	*b* = -8.87 [-11.46, -6.28], *t* = -6.72, *p* < .001		*b* = 4.16 [1.81, 6.51], *t* = 3.48, *p* = .001
**Fair**	Mean = 57.43, SD = 13.51	Mean = 53.00, SD = 10.56	Mean = 57.99, SD = 14.60	Mean = 55.28, SD = 13.14	Mean = 56.02, SD = 14.17	Mean = 52.80, SD = 16.05	Mean = 58.08, SD = 13.93	Mean = 60.12, SD = 14.55
		b = -4.43 [-6.39, -2.46], *t* = -4.42, *p* < .001	*b* = 0.56 [-1.71, 2.82], *t* = 0.48, *p* = .630	*b* = -2.15 [-4.27, -0.03], *t* = -1.99, *p* = .047	*b* = -1.41 [-3.63, 0.80], *t* = -1.26, *p* = .210	*b* = -4.63 [-7.00, -2.26], *t* = -3.84, *p* < .001		*b* = 2.04 [-0.25, 4.34], *t* = 1.75, *p* = .081
**Competent**	Mean = 60.55, SD = 15.95	Mean = 54.52, SD = 11.45	Mean = 59.22, SD = 16.42	Mean = 58.05, SD = 14.72	Mean = 58.51, SD = 16.24	Mean = 54.21, SD = 17.15	Mean = 59.89, SD = 14.88	Mean = 60.78, SD = 14.79
		b = -6.03 [-8.28, -3.78], *t* = -5.26, *p* < .001	*b* = -1.33 [-3.94, 1.28], *t* = -1.00, *p* = .319	*b* = -2.50 [-4.94, -0.06], *t* = -2.02, *p* = .044	*b* = -2.04 [-4.61, 0.54], *t* = -1.56, *p* = .120	*b* = -6.34 [-8.98, -3.69], *t* = -4.71, *p* < .001		*b* = 0.88 [-1.51, 3.27], *t* = 0.72, *p* = .469

Table S4a. "Baseline" ratings across conditions of Study 2. We analyze "baseline" ratings of Actors on each of our DVs, when Evaluators are given no information about the Actor's punishment or looking behavior. We report descriptive statistics within each condition (first rows) and, for each treatment, compare ratings within the relevant treatment to ratings in the baseline condition (second rows; significant differences are highlighted in blue).

3.3.2 Ratings of Actors who did vs. did not punish

	Study 2a						Study 2b	
	Democrat	Other participant	Democrat + Ignored injustice	Democrat + Conflict of Interest (Police)	Democrat + Conflict of Interest (Privacy)	Democrat + Previously Independent	Democrat	Democrat + Responded to Injustice
**Positivity**	b = 30.68 [26.84, 34.52], t = 15.73, p < .001	b = 39.07 [34.68, 43.46], t = 17.53, p < .001	b = 29.51 [25.64, 33.37], t = 15.03, p < .001	b = 28.80 [25.10, 32.49], t = 15.34, p < .001	b = 27.89 [24.37, 31.42], t = 15.57, p < .001	b = 26.28 [22.61, 29.95], t = 14.10, p < .001	b = 23.32 [19.71, 26.93], t = 12.72, p < .001	b = 29.37 [25.62, 33.12], t = 15.42, p < .001
		b = 8.39 [2.57, 14.20], t = 2.83, p = .005	b = -1.17 [-6.61, 4.26], t = -0.42, p = .672	b = -1.89 [-7.20, 3.43], t = -0.70, p = .486	b = -2.79 [-7.99, 2.41], t = -1.05, p = .293	b = -4.40 [-9.70, 0.90], t = -1.63, p = .103		b = 6.05 [0.86, 11.24], t = 2.29, p = .022
**Sharing**	b = 18.87 [15.82, 21.93], t = 12.15, p < .001	b = 24.64 [21.12, 28.15], t = 13.79, p < .001	b = 18.97 [15.97, 21.97], t = 12.45, p < .001	b = 16.17 [13.32, 19.02], t = 11.17, p < .001	b = 16.16 [13.32, 19.00], t = 11.20, p < .001	b = 15.49 [12.44, 18.54], t = 10.00, p < .001	b = 13.70 [10.71, 16.69], t = 9.02, p < .001	b = 18.51 [15.49, 21.54], t = 12.06, p < .001
		b = 5.76 [1.11, 10.41], t = 2.43, p = .015	b = 0.09 [-4.18, 4.37], t = 0.04, p = .965	b = -2.71 [-6.87, 1.46], t = -1.27, p = .203	b = -2.72 [-6.88, 1.45], t = -1.28, p = .201	b = -3.38 [-7.69, 0.93], t = -1.54, p = .124		b = 4.82 [0.58, 9.06], t = 2.23, p = .026
**Loyal**	b = 40.08 [36.79, 43.37], t = 23.98, p < .001	b = 47.96 [44.54, 51.38], t = 27.63, p < .001	b = 34.18 [30.85, 37.52], t = 20.16, p < .001	b = 34.49 [31.57, 37.41], t = 23.24, p < .001	b = 34.46 [31.34, 37.58], t = 21.72, p < .001	b = 29.44 [26.53, 32.34], t = 19.97, p < .001	b = 33.95 [30.81, 37.08], t = 21.31, p < .001	b = 39.25 [35.95, 42.54], t = 23.42, p < .001
		b = 7.88 [3.15, 12.61], t = 3.27, p = .001	b = -5.90 [-10.57, -1.22], t = -2.48, p = .014	b = -5.59 [-9.98, -1.21], t = -2.50, p = .013	b = -5.62 [-10.15, -1.10], t = -2.44, p = .015	b = -10.65 [-15.02, -6.27], t = -4.78, p < .001		b = 5.30 [0.76, 9.84], t = 2.29, p = .022
**Fair**	b = 22.85 [19.84, 25.86], t = 14.91, p < .001	b = 29.65 [26.08, 33.23], t = 16.33, p < .001	b = 21.95 [18.86, 25.04], t = 13.98, p < .001	b = 21.40 [18.50, 24.31], t = 14.50, p < .001	b = 19.74 [17.04, 22.45], t = 14.35, p < .001	b = 19.46 [16.73, 22.19], t = 14.01, p < .001	b = 19.50 [16.51, 22.49], t = 12.84, p < .001	b = 25.13 [21.86, 28.39], t = 15.15, p < .001
		b = 6.80 [2.14, 11.47], t = 2.87, p = .004	b = -0.90 [-5.20, 3.41], t = -0.41, p = .683	b = -1.45 [-5.62, 2.73], t = -0.68, p = .497	b = -3.11 [-7.15, 0.94], t = -1.51, p = .132	b = -3.39 [-7.45, 0.67], t = -1.64, p = .101		b = 5.63 [1.22, 10.04], t = 2.50, p = .013
**Competent**	b = 21.59 [18.65, 24.53], t = 14.44, p < .001	b = 27.95 [24.50, 31.40], t = 15.95, p < .001	b = 19.09 [16.07, 22.11], t = 12.45, p < .001	b = 18.88 [16.15, 21.61], t = 13.61, p < .001	b = 18.60 [15.97, 21.22], t = 13.95, p < .001	b = 17.38 [14.78, 19.98], t = 13.15, p < .001	b = 17.25 [14.57, 19.93], t = 12.67, p < .001	b = 22.39 [19.38, 25.39], t = 14.66, p < .001
		b = 6.36 [1.84, 10.88], t = 2.76, p = .006	b = -2.50 [-6.70, 1.71], t = -1.17, p = .244	b = -2.71 [-6.71, 1.29], t = -1.33, p = .184	b = -2.99 [-6.92, 0.94], t = -1.49, p = .136	b = -4.21 [-8.13, -0.29], t = -2.11, p = .035		b = 5.14 [1.12, 9.15], t = 2.51, p = .012

Table S4b. Evaluations of punishment across conditions of Study 2. We analyze ratings of Actors who did vs. did not punish on each of our DVs, when Evaluators are given no information about looking. We report the effect of punishment on the relevant DV within each condition (first rows; significant effects are highlighted in grey) and, for each treatment, compare the effect of punishment within the relevant treatment vs. the baseline condition (i.e., we test for punishment X treatment interactions) (second rows; significant interactions are highlighted in blue).

3.3.3 Ratings of Actors who punished without vs. with looking

	Study 2a (Democrats)						Study 2b (Democrats)	
	Democrat	Other participant	Democrat + Ignored injustice	Democrat + Conflict of Interest (Police)	Democrat + Conflict of Interest (Privacy)	Democrat + Previously Independent	Democrat	Democrat + Responded to Injustice
**Positivity**	b = -10.39 [-13.15, -7.64], t = -7.43, p < .001	b = -11.35 [-14.87, -7.84], t = -6.35, p < .001	b = -12.46 [-15.32, -9.60], t = -8.58, p < .001	b = -12.21 [-14.80, -9.62], t = -9.27, p < .001	b = -13.75 [-16.52, -10.98], t = -9.78, p < .001	b = -10.44 [-13.12, -7.76], t = -7.67, p < .001	b = -13.28 [-16.26, -10.29], t = -8.76, p < .001	b = -10.34 [-13.33, -7.36], t = -6.81, p < .001
		b = -0.96 [-5.41, 3.50], t = -0.42, p = .673	b = -2.07 [-6.02, 1.89], t = -1.03, p = .306	b = -1.81 [-5.59, 1.96], t = -0.94, p = .345	b = -3.36 [-7.25, 0.54], t = -1.69, p = .091	b = -0.05 [-3.88, 3.78], t = -0.02, p = .981		b = 2.93 [-1.28, 7.14], t = 1.37, p = .172
**Sharing**	b = -3.48 [-5.84, -1.11], t = -2.89, p = .004	b = -6.19 [-8.57, -3.81], t = -5.12, p < .001	b = -7.63 [-9.76, -5.49], t = -7.04, p < .001	b = -6.79 [-9.04, -4.53], t = -5.91, p < .001	b = -7.38 [-9.57, -5.19], t = -6.63, p < .001	b = -5.66 [-7.84, -3.48], t = -5.11, p < .001	b = -8.63 [-11.07, -6.19], t = -6.96, p < .001	b = -5.54 [-7.86, -3.23], t = -4.70, p < .001
		b = -2.72 [-6.06, 0.63], t = -1.59, p = .111	b = -4.15 [-7.33, -0.97], t = -2.57, p = .011	b = -3.31 [-6.57, -0.05], t = -1.99, p = .047	b = -3.91 [-7.12, -0.69], t = -2.39, p = .017	b = -2.18 [-5.39, 1.03], t = -1.33, p = .182		b = 3.09 [-0.27, 6.44], t = 1.81, p = .072
**Loyal**	b = 4.31 [1.60, 7.01], t = 3.13, p = .002	b = 7.47 [4.51, 10.44], t = 4.96, p < .001	b = -0.71 [-3.25, 1.83], t = -0.55, p = .584	b = 1.69 [-0.78, 4.17], t = 1.35, p = .178	b = -0.41 [-3.13, 2.32], t = -0.29, p = .769	b = 1.54 [-1.09, 4.17], t = 1.15, p = .250	b = 7.00 [4.33, 9.66], t = 5.17, p < .001	b = 3.34 [1.11, 5.56], t = 2.95, p = .003
		b = 3.16 [-0.84, 7.17], t = 1.55, p = .121	b = -5.02 [-8.72, -1.31], t = -2.66, p = .008	b = -2.61 [-6.27, 1.04], t = -1.40, p = .161	b = -4.72 [-8.55, -0.88], t = -2.42, p = .016	b = -2.77 [-6.53, 0.99], t = -1.44, p = .149		b = -3.66 [-7.12, -0.20], t = -2.08, p = .038
**Fair**	b = -17.59 [-20.43, -14.75], t = -12.19, p < .001	b = -18.81 [-22.11, -15.52], t = -11.24, p < .001	b = -17.91 [-20.85, -14.98], t = -12.02, p < .001	b = -16.42 [-19.08, -13.77], t = -12.17, p < .001	b = -17.84 [-20.67, -15.00], t = -12.39, p < .001	b = -11.62 [-14.23, -9.02], t = -8.78, p < .001	b = -18.60 [-21.53, -15.66], t = -12.47, p < .001	b = -13.08 [-15.72, -10.44], t = -9.75, p < .001
		b = -1.22 [-5.56, 3.11], t = -0.55, p = .580	b = -0.32 [-4.40, 3.75], t = -0.16, p = .876	b = 1.17 [-2.71, 5.05], t = 0.59, p = .555	b = -0.25 [-4.25, 3.75], t = -0.12, p = .903	b = 5.96 [2.12, 9.81], t = 3.05, p = .002		b = 5.52 [1.58, 9.45], t = 2.75, p = .006
**Competent**	b = -12.20 [-14.64, -9.76], t = -9.83, p < .001	b = -12.20 [-15.12, -9.28], t = -8.21, p < .001	b = -13.24 [-15.92, -10.56], t = -9.72, p < .001	b = -12.16 [-14.42, -9.89], t = -10.57, p < .001	b = -12.75 [-15.37, -10.12], t = -9.54, p < .001	b = -9.40 [-11.79, -7.02], t = -7.75, p < .001	b = -11.88 [-14.48, -9.28], t = -8.99, p < .001	b = -9.42 [-11.91, -6.92], t = -7.43, p < .001
		b = -0.00 [-3.80, 3.80], t = -0.00, p = .999	b = -1.04 [-4.66, 2.58], t = -0.56, p = .573	b = 0.04 [-3.28, 3.36], t = 0.03, p = .980	b = -0.55 [-4.13, 3.03], t = -0.30, p = .764	b = 2.79 [-0.61, 6.20], t = 1.61, p = .108		b = 2.46 [-1.14, 6.05], t = 1.34, p = .180

Table S4c. Evaluations of punishment without vs. with looking across conditions of Study 2. We repeat our approach from Table S4b, but analyze ratings of Actors who punished without vs. with looking. We thus report effects of not looking, conditional on punishing (and report both simple effects of not looking, and not looking X treatment interactions).

3.3.4 Ratings of Actors who did not punish without vs. with looking

	Study 2a						Study 2b	
	Democrat	Other participant	Democrat + Ignored injustice	Democrat + Conflict of Interest (Police)	Democrat + Conflict of Interest (Privacy)	Democrat + Previously Independent	Democrat	Democrat + Responded to Injustice
Positivity	*b* = -11.56 [-13.97, -9.15], *t* = -9.44, *p* < .001	*b* = -11.39 [-13.93, -8.86], *t* = -8.85, *p* < .001	*b* = -11.30 [-14.05, -8.56], *t* = -8.10, *p* < .001	*b* = -13.54 [-15.95, -11.13], *t* = -11.04, *p* < .001	*b* = -12.30 [-14.76, -9.85], *t* = -9.87, *p* < .001	*b* = -11.54 [-13.89, -9.18], *t* = -9.63, *p* < .001	*b* = -13.79 [-16.50, -11.09], *t* = -10.03, *p* < .001	*b* = -11.74 [-14.40, -9.08], *t* = -8.69, *p* < .001
		b = 0.17 [-3.32, 3.65], *t* = 0.09, *p* = .926	*b* = 0.26 [-3.39, 3.90], *t* = 0.14, *p* = .890	*b* = -1.98 [-5.39, 1.42], *t* = -1.14, *p* = .253	*b* = -0.75 [-4.18, 2.69], *t* = -0.43, *p* = .670	*b* = 0.02 [-3.34, 3.39], *t* = 0.01, *p* = .989		*b* = 2.05 [-1.73, 5.84], *t* = 1.06, *p* = .288
Sharing	*b* = -7.85 [-9.86, -5.83], *t* = -7.67, *p* < .001	*b* = -7.82 [-9.92, -5.72], *t* = -7.34, *p* < .001	*b* = -7.04 [-8.84, -5.24], *t* = -7.70, *p* < .001	*b* = -7.27 [-9.11, -5.44], *t* = -7.79, *p* < .001	*b* = -6.95 [-8.98, -4.92], *t* = -6.74, *p* < .001	*b* = -8.09 [-10.07, -6.11], *t* = -8.03, *p* < .001	*b* = -7.91 [-9.99, -5.83], *t* = -7.50, *p* < .001	*b* = -8.98 [-11.33, -6.63], *t* = -7.51, *p* < .001
		b = 0.03 [-2.87, 2.93], *t* = 0.02, *p* = .985	*b* = 0.80 [-1.89, 3.50], *t* = 0.59, *p* = .559	*b* = 0.57 [-2.15, 3.29], *t* = 0.42, *p* = .678	*b* = 0.89 [-1.96, 3.75], *t* = 0.62, *p* = .539	*b* = -0.24 [-3.06, 2.57], *t* = -0.17, *p* = .865		*b* = -1.07 [-4.19, 2.06], *t* = -0.67, *p* = .504
Loyal	*b* = -6.86 [-9.08, -4.64], *t* = -6.08, *p* < .001	*b* = -6.51 [-8.73, -4.29], *t* = -5.76, *p* < .001	*b* = -6.55 [-9.00, -4.10], *t* = -5.27, *p* < .001	*b* = -6.93 [-9.25, -4.61], *t* = -5.87, *p* < .001	*b* = -6.96 [-9.07, -4.85], *t* = -6.49, *p* < .001	*b* = -5.71 [-7.60, -3.82], *t* = -5.95, *p* < .001	*b* = -7.67 [-9.90, -5.44], *t* = -6.77, *p* < .001	*b* = -6.63 [-9.00, -4.26], *t* = -5.51, *p* < .001
		b = 0.35 [-2.78, 3.49], *t* = 0.22, *p* = .825	*b* = 0.31 [-2.98, 3.61], *t* = 0.19, *p* = .853	*b* = -0.07 [-3.28, 3.14], *t* = -0.04, *p* = .965	*b* = -0.10 [-3.16, 2.95], *t* = -0.07, *p* = .947	*b* = 1.15 [-1.75, 4.06], *t* = 0.78, *p* = .436		*b* = 1.04 [-2.21, 4.28], *t* = 0.63, *p* = .530
Fair	*b* = -16.96 [-19.75, -14.16], *t* = -11.93, *p* < .001	*b* = -17.11 [-20.11, -14.11], *t* = -11.23, *p* < .001	*b* = -15.50 [-18.52, -12.47], *t* = -10.09, *p* < .001	*b* = -16.36 [-19.11, -13.61], *t* = -11.71, *p* < .001	*b* = -16.55 [-19.22, -13.89], *t* = -12.23, *p* < .001	*b* = -13.17 [-15.61, -10.73], *t* = -10.63, *p* < .001	*b* = -17.50 [-20.55, -14.44], *t* = -11.26, *p* < .001	b = -15.50 [-18.32, -12.68], *t* = -10.81, *p* < .001
		b = -0.15 [-4.24, 3.94], *t* = -0.07, *p* = .942	*b* = 1.46 [-2.65, 5.57], *t* = 0.70, *p* = .486	*b* = 0.60 [-3.31, 4.51], *t* = 0.30, *p* = .763	*b* = 0.40 [-3.45, 4.26], *t* = 0.21, *p* = .837	*b* = 3.79 [0.08, 7.49], *t* = 2.01, *p* = .045		*b* = 2.00 [-2.15, 6.15], *t* = 0.95, *p* = .345
Competent	*b* = -12.19 [-14.74, -9.64], *t* = -9.40, *p* < .001	*b* = -14.79 [-17.49, -12.08], *t* = -10.77, *p* < .001	*b* = -13.56 [-16.44, -10.68], *t* = -9.26, *p* < .001	*b* = -15.17 [-17.79, -12.55], *t* = -11.38, *p* < .001	*b* = -15.15 [-17.78, -12.51], *t* = -11.30, *p* < .001	*b* = -12.05 [-14.39, -9.71], *t* = -10.13, *p* < .001	*b* = -14.58 [-17.31, -11.86], *t* = -10.53, *p* < .001	*b* = -13.03 [-15.82, -10.23], *t* = -9.17, *p* < .001
		b = -2.59 [-6.30, 1.12], *t* = -1.37, *p* = .170	*b* = -1.36 [-5.20, 2.47], *t* = -0.70, *p* = .485	*b* = -2.98 [-6.63, 0.67], *t* = -1.60, *p* = .110	*b* = -2.95 [-6.62, 0.71], *t* = -1.58, *p* = .114	*b* = 0.14 [-3.32, 3.59], *t* = 0.08, *p* = .937		*b* = 1.56 [-2.34, 5.45], *t* = 0.78, *p* = .433

Table S4d. Evaluations of non-punishment without vs. with looking across conditions of Study 2. We repeat our approach from Table S4c, but analyze ratings of Actors who did not punish without vs. with looking. We thus report effects of not looking, conditional on not punishing.

3.3.5 Interpretation of analyses

Together, the above tables reveal that both baseline conditions of Study 2 replicated all reported Study 1 effects on all DVs. As in Study 1, within these conditions, we find that (i) punishers are evaluated more positivity than non-punishers on all DVs, (ii) punishers who decline (vs. choose) to look are evaluated less positively on all DVs except loyalty; for loyalty, punishment without looking is rated *more* positively, and (iii) non-punishers who decline (vs. choose) to look are evaluated less positively on all DVs.

Furthermore, the above tables provide insight into why our treatments had their effects. To highlight these insights, in the below sections we interpret the results for our treatments that sought to (i) cast doubt on loyalty by removing information, (ii) cast doubt on loyalty by adding information, and (iii) establish loyalty by adding information.

Casting doubt on loyalty by removing information

As reported in the main text, relative to the baseline condition, casting doubt on Actor loyalty by removing information (as we did in our "Other participant" treatment) successfully decreased "baseline" loyalty ratings (when Evaluators had no information about whether the Actor punished or looked), and increased the reputation value of punishment in general on both of our global evaluation DVs. However, it did not significantly influence the reputation value of punishment without vs. with looking on either global evaluation DV.

The above tables add further insight into these patterns. They reveal that, for *all* DVs (i.e., including loyalty, fairness and competence), the "Other participant" treatment significantly (i) decreased baseline ratings, (ii) increased the reputation value of punishment in general, and (iii) did not influence the reputation value of punishment without vs. with looking, or non-punishment without vs. with looking—with just one exception (the negative effect of "Other participant" on baseline sharing, which was only marginally significant). Thus, describing the Actor simply as "another participant" made Evaluators generally less positive about the Actor at baseline, and more sensitive to the question of whether or not the Actor decided to punish. However, once Evaluators knew whether the Actor punished, a lack of information about the Actor's party affiliation did *not* influence Evaluators' sensitivity to the Actor's looking decision.

This pattern of results suggests that Evaluators may have seen the choice to punish as very informative, such that punishment information served as a substitute for information about partisanship. Under this interpretation, removing information about Actor partisanship did not change Evaluators' relative assessments of punishment without vs. with looking, because Evaluators had punishment information (to compensate for a lack of partisanship information) when making these assessments. Thus, the informativeness of punishment may help to explain why we did not find support for the hypothesis that casting doubt on Actor loyalty by removing information can enhance the reputation value of punishment without looking.

Casting doubt on loyalty by adding information

As reported in the main text, relative to the baseline condition, casting doubt on loyalty by adding information (as we did in "Democrat + Ignored injustice", both "Democrat + Conflict of Interest" treatments, and "Democrat + Previously Independent") was largely successful at reducing baseline loyalty ratings (although "Democrat + Ignored injustice" did not have this effect). However, none of these treatments significantly (i) influenced the reputation value of punishment in general, or (ii) increased the reputation value of punishment specifically without looking, on either of our global evaluation DVs. In fact, "Democrat + Ignored injustice" and both "Democrat + Conflict of Interest" treatments caused Evaluators to share relatively *less* money with punishers who declined (vs. chose) to look.

The above tables add further insight into these patterns. First, they reveal that, beyond reducing baseline *loyalty* ratings, some of the aforementioned treatments also reduced baseline ratings of other DVs (in particular, positivity, fairness, and competence). Thus, the information that we added to cast doubt on Actors' loyalty caused Evaluators to evaluate Actors less positively at baseline in several ways.

Second, we find that none of the aforementioned treatments enhanced the reputation value of punishment in general on any DVs. In fact, all four treatments *decreased* the loyalty benefit of punishment (and one treatment also decreased the competence benefit of punishment). Thus, the information that we added to cast doubt on Actors' loyalty caused Evaluators to interpret punishment as a less meaningful signal of loyalty.

Third, we find that none of the aforementioned treatments enhanced the reputation value of punishment without (vs. with) looking on *any* DVs, with the one exception that "Democrat + Previously Independent" decreased the fairness cost of declining to look. Furthermore, in all four treatments, we did not observe the significant loyalty benefit of declining to look that we found in Study 1 (and replicated in the baseline conditions of Studies 2a-b). And for two treatments, this translated to a significant interaction effect, whereby the "not looking" effect size was significantly more negative in the relevant treatment than the baseline condition. Thus, we find evidence that the information that we added to cast doubt on Actors' loyalty caused Evaluators to interpret punishment without (vs. with) looking as a less meaningful signal of loyalty.

Finally, we find that none of the aforementioned treatments influenced the reputation value of non-punishment without vs. with looking on any DVs, with the one exception that "Democrat + Previously Independent" decreased the fairness cost of declining to look.

These patterns help illuminate why casting doubt on Actor loyalty by adding information did *not* cause the loyalty-signaling property of punishment without looking to pay larger reputational dividends, as one might have expected it to. We instead found that, when given active reason to doubt an Actor's loyalty, Evaluators formed more negative baseline impressions of Actors, and saw the choice to punish in general as a less meaningful indicator of loyalty.

Furthermore, Evaluators also saw the choice to punish specifically without looking as a less meaningful indicator of loyalty, perhaps because eschewing opposing perspectives seemed less like moral commitment, and more like laziness. And consequentially, casting doubt on Actor loyalty by adding information did not increase the reputation value of punishment in general, or punishment specifically without looking.

Establishing loyalty by adding information

As reported in the main text, relative to the baseline condition, establishing loyalty by adding information (as we did in "Democrat + Responded to injustice") successfully increased baseline loyalty ratings, and increased the reputation value of punishment in general on both of our global evaluation DVs. However, it did not significantly influence the reputation value of punishment without vs. with looking on either global evaluation DV.

The above tables add further insight into these patterns. First, they reveal that "Democrat + Responded to injustice" increased baseline loyalty ratings, but did not increase baseline ratings of other DVs.

Second, we find that "Democrat + Responded to injustice" increased the reputation value of punishment in general on all DVs. This effect can be interpreted as a "moral consistency" effect, whereby having displayed loyalty previously (by responding to injustice) increases the importance of displaying loyalty in the present study (by punishing).

Third, we find that "Democrat + Responded to injustice" decreased the fairness cost, but also decreased the loyalty benefit, of punishing without (vs. with) looking—and did not influence relative evaluations of punishment without looking on any other DVs. Thus, the information that we added to establish loyalty caused Evaluators to interpret punishment without looking as relatively less unfair, perhaps because it seemed less hasty or uninformed coming from an individual with an established record of engagement with the relevant moral cause. But establishing loyalty *also* caused Evaluators to interpret punishment without looking as a relatively less meaningful signal of loyalty, perhaps because loyalty was very well-established for both types of punishers. Together, these countervailing effects seem to have netted out to create no change in the overall reputation value of punishment without vs. with looking (as measured by our global evaluation DVs).

Finally, we find that "Democrat + Responded to injustice" did not influence the reputation value of non-punishment without vs. with looking on any DVs.

Together, these patterns help illuminate why establishing Actor loyalty by adding information served to increase the reputation value of punishment in general, but not punishment specifically without looking.

3.4 Mediation analyses

As noted in the main text, the baseline conditions of Studies 2a-b replicate the results of our exploratory mediation analyses from Studies 1a-b (which were pre-registered analyses for the baseline conditions of Study 2). In Table S5, we reproduce Table S2 but with data from the baseline conditions of Study 2; we find qualitatively identical patterns.

		Study 2a (Democrats) n = 302	Study 2b (Democrats) n = 292
Total Effect		b = -10.39 [-13.15, -7.64], t = -7.43, p < .001	b = -13.28 [-16.26, -10.30], t = -8.76, p < .001
Model with Fair & Loyal as mediators	Effects for Fair	Path A: b = -17.59 [-20.41, -14.76], z = -12.20, p < .001	Path A: b = -18.60 [-21.52, -15.68], z = -12.48, p < .001
		Path B: b = .62 [.54, .70], z = 15.55, p < .001	Path B: b = .70 [.61, .79], z = 15.88, p < .001
		Indirect effect: b = -10.92 [-13.15, -8.68]	Indirect effect: b = -13.03 [-15.64, -10.43]
	Effects for Loyal	Path A: b = 4.31 [1.61, 7.00], z = 3.13, p = .002	Path A: b = 7.00 [4.35, 9.65], z = 5.17, p < .001
		Path B: b = .26 [.19, .34], z = 6.93, p < .001	Path B: b = .18 [.10, .26], z = 4.38, p < .001
		Indirect effect: b = 1.13 [.36, 1.91]	Indirect effect: b = 1.28 [.53, 2.03]
	Direct effect	b = -.61 [-2.85, 1.62], z = -.54, p = .591	b = -1.52 [-4.10, 1.06], z = -1.16, p = .248
Model with Competent & Loyal as mediators	Effects for Competent	Path A: b = -12.20 [-14.63, -9.77], z = -9.84, p < .001	Path A: b = -11.88 [-14.46, -9.29], z = -9.00, p < .001
		Path B: b = .62 [.53, .71], z = 13.42, p < .001	Path B: b = .77 [.70, .83], z = 22.47, p < .001
		Indirect effect: b = -7.55 [-9.42, -5.69]	Indirect effect: b = -9.12 [-11.26, -6.98]
	Effects for Loyal	Path A: b = 4.31 [1.61, 7.00], z = 3.13, p = .002	Path A: b = 7.00 [4.35, 9.65], z = 5.17, p < .001
		Path B: b = .27 [.19, .34], z = 6.60, p < .001	Path B: b = .15 [.07, .22], z = 3.84, p < .001
		Indirect effect: b = 1.14 [.35, 1.93]	Indirect effect: b = 1.03 [.37, 1.68]
	Direct effect	b = -3.98 [-6.13, -1.83], z = -3.63, p < .001	b = -5.19 [-7.24, -3.14], z = -4.96, p < .001

Table S5. Mediation analyses of Studies 2a-b. We reproduce Table S2, but with the "baseline" conditions of Studies 2a-b.

We also explored mediation within the treatment conditions of Studies 2a-b. In particular, within each treatment condition, we ran the same two mediation models that we report results for above. The results of these analyses, which we do not report here for brevity, were qualitatively identical to the above-reported results, with one key exception. As illustrated in Table S4c, the four Study 2a treatments that were designed to cast doubt on loyalty by adding information (i.e., "Democrat + Ignored injustice", both "Democrat + Conflict of Interest" treatments, and "Democrat + Previously Independent") did not produce significant positive effects of punishment without (vs. with) looking on loyalty. Therefore, within each of these conditions, in both models, the A path and indirect effects for loyalty are not significant.

3.5 Moderation analyses

As noted in the main text, Study 2 provides further evidence, both within the baseline conditions and overall, for the moderating effects of Evaluator ideology (whereby strong partisans show relatively more positive evaluations of punishment in general, and less negative evaluations of punishment without looking).

To support this claim, in Table S6, we reproduce main text Table 2, but with data from the baseline conditions of Studies 2a-b. This table reveals significant partisanship moderation in the baseline condition of Study 2a for overall positivity (both for evaluations of punishment, and evaluations of punishment without vs. with looking) and sharing (for evaluations of punishment,

but not evaluations of punishment without vs. with looking). In the baseline condition of Study 2b, however, we do not find significant moderation.

	**Study 2a (Democrats)**<br>n **= 302**	
	**Evaluations of punishers vs. non-punishers**<br>(Positive coefficients reflect preferences for *punishment*)	**Evaluations of punishment without vs. with looking**<br>(Positive coefficeints reflect preferences for punishment *without* looking)
**Positivity**	Weak Partisans: $b = 22.55$ [16.72, 28.38], $t = 7.67$, $p < .001$<br>Strong Partisans: $b = 35.41$ [30.45, 40.36], $t = 14.10$, $p < .001$<br>Interaction: $b = 12.86$ [5.26, 20.46], $t = 3.33$, $p = .001$	Weak Partisans: $b = -15.66$ [-19.63, -11.69], $t = -7.82$, $p < .001$<br>Strong Partisans: $b = -7.34$ [-10.99, -3.68], $t = -3.96$, $p < .001$<br>Interaction: $b = 8.32$ [2.96, 13.68], $t = 3.06$, $p = .002$
**Sharing**	Weak Partisans: $b = 14.59$ [9.63, 19.56], $t = 5.82$, $p < .001$<br>Strong Partisans: $b = 21.36$ [17.49, 25.23], $t = 10.88$, $p < .001$<br>Interaction: $b = 6.77$ [0.51, 13.02], $t = 2.13$, $p = .034$	Weak Partisans: $b = -5.32$ [-8.91, -1.72], $t = -2.93$, $p = .004$<br>Strong Partisans: $b = -2.41$ [-5.53, 0.71], $t = -1.52$, $p = .129$<br>Interaction: $b = 2.91$ [-1.82, 7.63], $t = 1.21$, $p = .227$
	**Study 2b (Democrats)**<br>n **= 292**	
	**Evaluations of punishers vs. non-punishers**<br>(Positive coefficients reflect preferences for *punishment*)	**Evaluations of punishment without vs. with looking**<br>(Positive coefficeints reflect preferences for punishment *without* looking)
**Positivity**	Weak Partisans: $b = 21.90$ [17.30, 26.51], $t = 9.39$, $p < .001$<br>Strong Partisans: $b = 24.86$ [19.18, 30.55], $t = 8.64$, $p < .001$<br>Interaction: $b = 2.96$ [-4.32, 10.24], $t = 0.80$, $p = .424$	Weak Partisans: $b = -15.47$ [-19.37, -11.58], $t = -7.85$, $p < .001$<br>Strong Partisans: $b = -10.89$ [-15.48, -6.30], $t = -4.69$, $p < .001$<br>Interaction: $b = 4.58$ [-1.41, 10.57], $t = 1.51$, $p = .133$
**Sharing**	Weak Partisans: $b = 12.63$ [8.79, 16.47], $t = 6.50$, $p < .001$<br>Strong Partisans: $b = 14.86$ [10.17, 19.55], $t = 6.26$, $p < .001$<br>Interaction: $b = 2.23$ [-3.80, 8.25], $t = 0.73$, $p = .468$	Weak Partisans: $b = -8.22$ [-11.24, -5.20], $t = -5.38$, $p < .001$<br>Strong Partisans: $b = -9.07$ [-13.01, -5.14], $t = -4.56$, $p < .001$<br>Interaction: $b = -0.85$ [-5.78, 4.09], $t = -0.34$, $p = .735$

Table S6. The moderating role of ideology in Studies 2a-b. We reproduce Table 2 from the main text, but with Studies 2a-b.

Then, in Figures S3a-b, we reproduce Figure 2 from the main text, but separately show results among strong vs. weak partisans—both for positivity (Figure S3a) and sharing (Figure S3b). These figures provide insight into moderation patterns across conditions. For both DVs, across conditions, we see that strong partisans generally show more positive evaluations of punishment in general, and less negative evaluations of punishment without looking. Notably, however, across all conditions, even strong partisans consistently react more negatively to punishers who decline vs. choose to look, on both DVs.

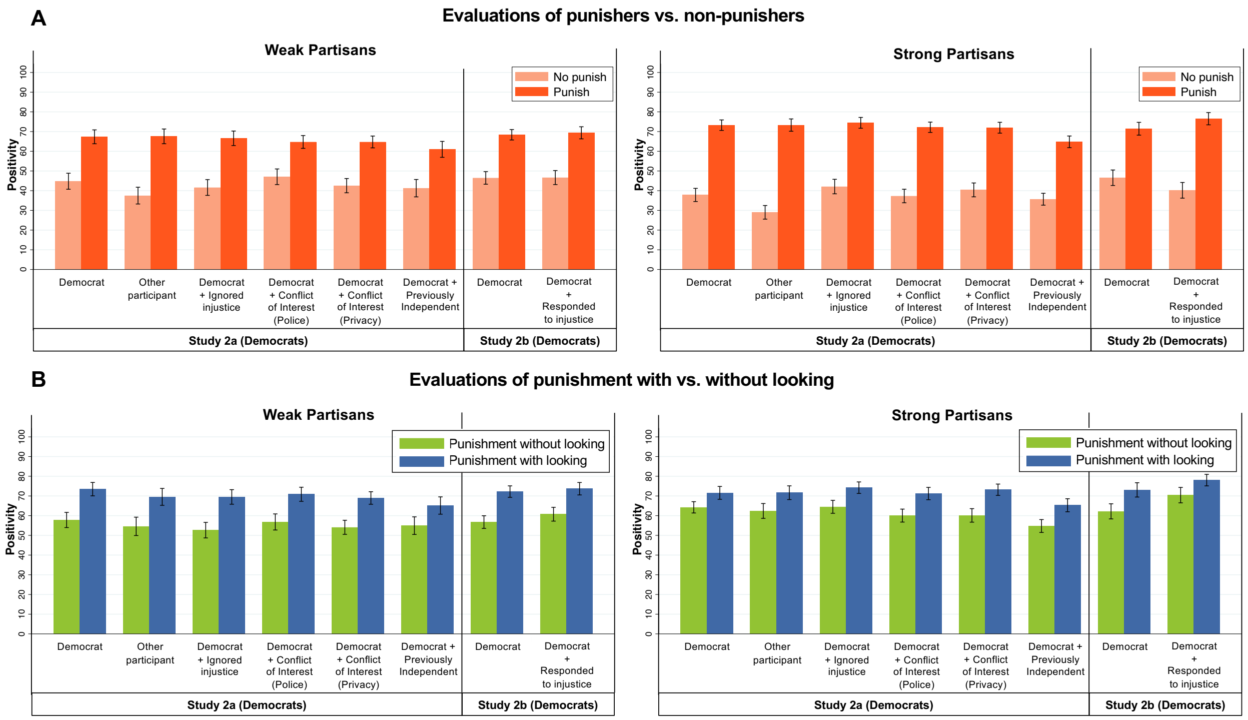

Figure S3a. The moderating role of ideology on positivity ratings in Studies 2a-b. We reproduce Figure 2 from the main text, but separately show results among strong vs. weak partisans.

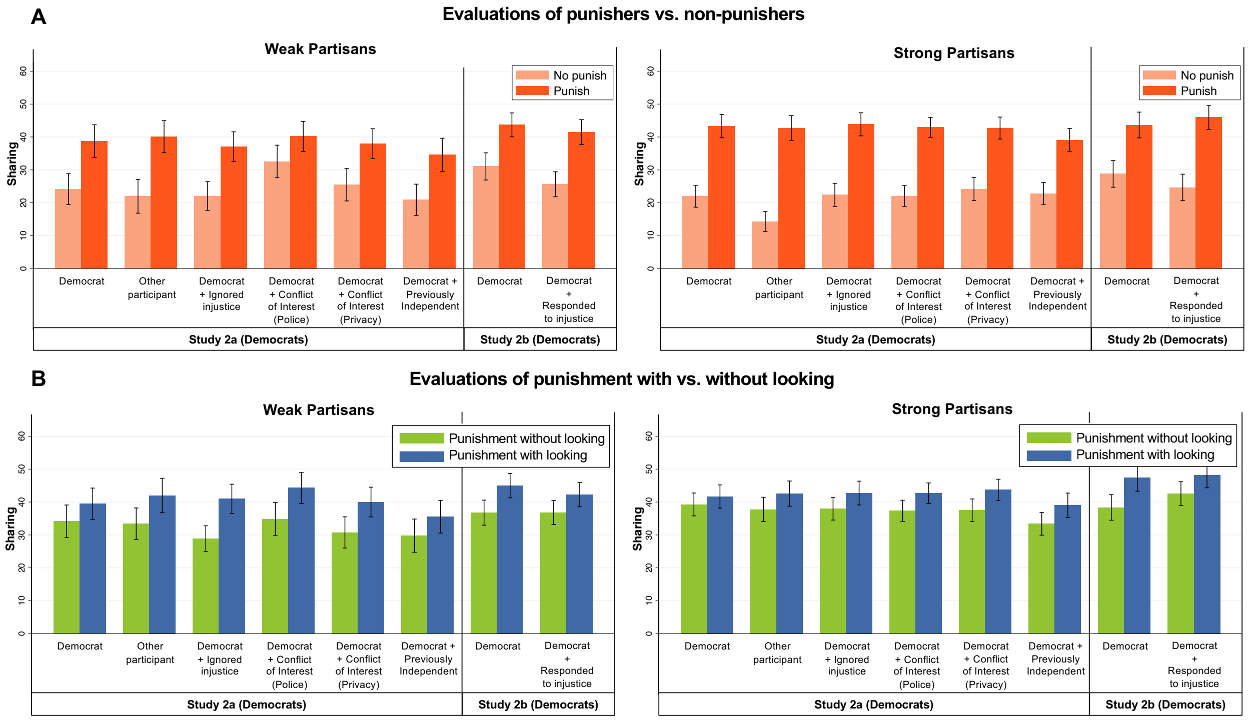

Figure S3b. The moderating role of ideology on sharing in Studies 2a-b. We reproduce Figure 2 from the main text, but separately show results among strong vs. weak partisans, and use our sharing DV.

4. Supplemental Analyses of Studies 3-4

4.1 Analyses of Study 4a by batch, and corrections for "peeking"

As described in the main text, for Study 4a, we pre-registered and recruited an initial target n = 1200 subjects. Upon reaching this initial target, all of our key results were significant. However, while finishing data collection for Study 4b (which recruited Republicans, who are under-represented on MTurk), we decided to direct Democrats to Study 4a, increasing its sample size. After making this decision, we registered an amendment to our Study 4a pre-registration, increasing our target to n = 2000 subjects. In the main text, we report analyses of Study 4a that combine data from both batches of data collection. However, in Table S7, we report our Study 4a results within each individual batch (and also re-report the overall results from both batches combined, to facilitate comparison).

We also pre-registered a plan to correct, in our combined analyses of both batches, for the fact that we "peeked" at first batch of data before deciding to collect a second batch (using the approach of Sagarin, Ambler, & Lee, 2014, Perspectives on Psychological Science). In particular, we planned, for each key result that is significant in our final sample, to report whether the result continues to be significant when accounting for peeking by computing an adjusted alpha threshold that allows us to maintain an actual type-I error rate of .05.

More precisely, instead of computing a single adjusted alpha threshold, we planned to report an adjusted alpha range. This reflects that the required alpha threshold depends on the maximum p-value observed in the initial batch of data collection for which we would have collected more data rather than declaring the initial results non-significant; this could range from a "best-case scenario" of the p-value observed after the initial data collection to a "worst-case scenario" of 1.

Thus, for each result in Table S7, we report a best- and worst-case scenario for the adjusted alpha threshold. Importantly, for each of the three results that are significant in the overall sample, we find that the p-values are smaller than even the worst-case scenario adjusted alpha threshold. Thus, we find that all significant Study 4a results remain significant, even after accounting for peeking.

	**Effect of making punishment observable on…**		**Effect of making looking observable on…**	
	Punishment overall	Punishment without looking	Looking overall	Punishment without looking
Batch 1 (n = 1222)	b = .09 [.03, .15], t = 3.03, p = .003, n = 815	b = .05 [.002, .09], t = 2.05, p = .040, n = 815	b = .12 [.06, .19], t = 3.56, p < .001, n = 797	b = -.03 [-.07, .02], t = -1.06, p = .288, n = 797
Batch 2 (n = 752)	b = .16 [.09, .24], t = 4.18, p < .001, n = 504	b = .07 [.01, .13], t = 2.45, p = .015, n = 504	b = -.01 [-.09, .08], t = -.11, p = .911, n = 487	b = -.03 [-.09, .03], t = -.93, p = .353, n = 487
Overall (n = 1974)	b = .12 [.07, .16], t = 4.98, p < .001, n = 1319	b = .06 [.02, .09], t = 3.13, p = .002, n = 1319	b = .08 [.02, .13], t = 2.72, p = .007, n = 1284	b = -.03 [-.07, .01], t = -1.41, p = .158, n = 1284
Adjusted Alpha Threshold: Best case	0.049998			0.032899
Adjusted Alpha Threshold: Worst case	0.031013		0.031099	

Table S7. Analyses of Study 4a by batch, and adjusted Alpha Thresholds that account for "peeking" at data between batches.

4.2 Analyses of how making punishment observable influences looking

In our main text analyses of Studies 3-4, when considering the effects of making punishment observable (i.e., when comparing our "Nothing Observable" and "Punishment Observable" conditions), we focus on rates of punishment overall, and punishment without looking, as dependent variables. However, our design also allows us to ask how making punishment observable influences overall rates of *looking*. We pre-registered analyses of this

question, but do not report them in the main text because they are less relevant to our key theoretical questions. Here, we report these analyses.

In Study 3, among Democrats, rates of looking were similar in Punishment Observable (35%) and Nothing Observable (34%), $b = .01$ [-.04, .06], $t = .40$, $p = .686$, $n = 1222$. Republicans were also similarly likely to look in Punishment Observable (24%) and Nothing Observable (28%), $b = -.04$ [-.09, .01], $t = -1.45$, $p = .148$, $n = 1214$.

In Study 4, among Democrats, rates of looking were significantly higher in Punishment Observable (41%) than in Nothing Observable (34%), $b = .07$ [.02, .12], $t = 2.73$, $p = .006$, $n = 1319$. In contrast, Republicans were similarly likely to look in Punishment Observable (15%) and Nothing Observable (18%), $b = -.03$ [-.08, .02], $t = -1.08$, $p = .279$, $n = 763$.

Thus, we mostly find that making punishment observable has no reliable effect on rates of looking. However, in one case (Democrats in Study 4), making punishment observable increased looking.

4.3 Analyses of how making looking observable influences punishment

In our main text analyses of Studies 3-4, when considering the effects of making looking observable (i.e., when comparing our "Punishment Observable" and "Both Observable" conditions), we focus on rates of looking overall, and punishment without looking, as dependent measures. However, our design also allows us to ask how making looking observable influences overall rates of *punishment*. We likewise pre-registered analyses of this question, but do not report them in the main text because they are less relevant to our key theoretical questions. Here, we report these analyses.

In Study 3, among Democrats, rates of punishment were similar in Both Observable (24%) and Punishment Observable (23%), $b = .01$ [-.04, .05], $t = .27$, $p = .786$, $n = 1206$. Republicans were also similarly likely to punish in Both Observable (28%) and Punishment Observable (27%), $b = .02$ [-.03, .07], $t = .72$, $p = .474$, $n = 1197$.

In Study 4, among Democrats, rates of punishment were similar in Both Observable (28%) and Punishment Observable (30%), $b = -.02$ [-.07, .03], $t = -.77$, $p = .439$, $n = 1284$. Republicans were also similarly likely to punish in Both Observable (25%) and Punishment Observable (25%), $b = -.003$ [-.06, .06], $t = -.10$, $p = .921$, $n = 779$.

Thus, making looking observable did not influence rates of punishment in our studies.

4.4 Analyses of perfect comprehenders

As reported in the main text, our main text analyses include all subjects, regardless of performance on comprehension questions. However, per our pre-registration, below we report secondary analyses that restrict to subjects who correctly answered our comprehension questions on their first try (and produce very similar results).

We note that in Studies 3-4, subjects answered two sets of comprehension questions. The first three questions tested comprehension of the Dictator Game structure; for these questions, the correct responses were the same across observability conditions. The second three questions tested comprehension of the extent to which subjects' punishment and looking decisions were observable to the Evaluator; for these questions, the correct responses differed across observability conditions.

Importantly, in the below analyses, rather than restricting to subjects who answered *all* questions correctly on their first try, we merely restrict to subjects who correctly answered the Dictator Game questions on the first try. This decision reflects that in both Study 3 and Study 4,

initial performance on each of the three observability questions differed significantly across observability conditions; thus, restricting to subjects who correctly answered all observability questions on their first try would undermine random assignment and thus causal inference.

In particular, in Study 3, a series of chi squared tests reveals significant condition effects on binary variables indicating whether the first (Democrats: $X^2 = 30.37$, $p < .001$; Republicans: $X^2 = 34.10$, $p < .001$), second (Democrats: $X^2 = 174.58$, $p < .001$; Republicans: $X^2 = 178.69$, $p < .001$), and third (Democrats: $X^2 = 6.81$, $p = .033$; Republicans: $X^2 = 13.68$, $p = .001$) observability questions were initially answered correctly. Similarly, in Study 4, condition significantly predicted initial performance on the first (Democrats: $X^2 = 92.94$, $p < .001$; Republicans: $X^2 = 38.76$, $p < .001$), second (Democrats: $X^2 = 204.96$, $p < .001$; Republicans: $X^2 = 146.42$, $p < .001$), and third (Democrats: $X^2 = 13.54$, $p = .001$; Republicans: $X^2 = 6.83$, $p = .033$) observability questions.

4.4.1 Effects of making punishment observable

First, we report effects of making *punishment* observable (i.e., comparisons between our Nothing Observable and Punishment Observable conditions) among perfect comprehenders.

In Study 3, among Democrats, overall rates of punishment were significantly higher in Punishment Observable (24%) than Nothing Observable (18%), $b = .07$ [.02, .12], $t = 2.83$, $p = .005$, $n = 1080$. However, rates of punishment without looking did not significantly differ across the Punishment Observable (13%) and Nothing Observable (10%) conditions, $b = .03$ [-.01, .07], $t = 1.53$, $p = .127$, $n = 1080$. Among Republicans, overall rates of punishment were marginally higher in Punishment Observable (28%) than Nothing Observable (22%), $b = .05$ [-.001, .11], $t = 1.91$, $p = .056$, $n = 1022$. Furthermore, Republicans were significantly more likely to punish without looking in Punishment Observable (20%) than in Nothing Observable (14%), $b = .06$ [.02, .11], $t = 2.65$, $p = .008$, $n = 1022$.

In Study 4, among Democrats, overall rates of punishment were significantly higher in Punishment Observable (31%) than Nothing Observable (18%), $b = .13$ [.08, .18], $t = 5.22$, $p < .001$, $n = 1172$. Moreover, we find significantly higher rates of punishment without looking in Punishment Observable (16%) than Nothing Observable (9%), $b = .07$ [.03, .11], $t = 3.50$, $p < .001$, $n = 1172$. Similarly, Republicans were more likely to punish in Punishment Observable (26%) than Nothing Observable (16%), $b = .10$ [.03, .16], $t = 3.02$, $p = .003$, $n = 659$. And they were also more likely to punish without looking in Punishment Observable (20%) than in Nothing Observable (13%), $b = .08$ [.02, .13], $t = 2.59$, $p = .010$, $n = 659$.

4.4.2 Effects of making looking observable

Next, report effects of making *looking* observable (i.e., comparisons between our Punishment Observable and Both Observable conditions) among perfect comprehenders.

In Study 3, among Democrats, overall rates of looking were significantly higher in Both Observable (52%) than Punishment Observable (36%), $b = .16$ [.10, .22], $t = 5.41$, $p < .001$, $n = 1056$. Furthermore, rates of punishment without looking were significantly lower in Both Observable (8%) than Punishment Observable (13%), $b = -.05$ [-.09, -.01], $t = -2.64$, $p = .009$, $n = 1056$. Among Republicans, we likewise observed higher rates of looking in Both Observable (39%) than Punishment Observable (24%), $b = .15$ [.09, .21], $t = 5.13$, $p < .001$, $n = 986$. However, we observed no significant difference between rates of punishment without looking in Both Observable (16%) and Punishment Observable (20%), $b = -.04$ [-.09, .01], $t = -1.57$, $p = .116$, $n = 986$.

In Study 4, among Democrats, overall rates of looking were significantly higher in Both Observable (50%) than in Punishment Observable (42%), $b = .07$ [.02, .13], $t = 2.52$, $p = .012$, $n = 1137$. However, we found no significant difference between rates of punishment without looking in Both Observable (13%) versus Punishment Observable (16%), $b = -.03$ [-.07, .01], $t = -1.47$, $p = .142$, $n = 1137$. Among Republicans, we likewise saw higher rates of looking in Both Observable (30%) than Punishment Observable (16%), $b = .14$ [.08, .20], $t = 4.36$, $p < .001$, $n = 676$. However, we found no significant difference in rates of punishment without looking between Both Observable (20%) and Punishment Observable (20%), $b = -.001$ [-.06, .06], $t = -.03$, $p = .972$, $n = 676$.

4.5 Analyses of alternative specifications of looking

As described in the main text, per our pre-registration, our primary analyses define "looking" in Studies 3-4 as clicking at least one link to an opposing perspective article. However, for Study 4 (but not Study 3), we also preregistered secondary analyses that define looking as either (i) time spent on the "looking" screen (in which subjects were presented with links to opposing perspective articles, and invited to search the Internet for other opposing perspectives) or (ii) the continuous number of opposing perspective article links that subjects clicked. We report these alternative analyses (which produce very similar results) for both Studies 3-4, below.

4.5.1 Looking time (natural-log transformed seconds)

We begin by defining looking in terms of time spent on the "looking" screen. Per our pre-registration, we specifically consider the number of natural-log transformed seconds spent on this screen.

First, we use this new definition of looking to analyze overall rates of looking. In particular, we report the effect of making looking observable on overall rates of looking. In Study 3, among Democrats, we find that subjects spent more time looking in Both Observable ($M = 3.66$) than Punishment Observable ($M = 3.16$), $b = .50$ [.35, .65], $t = 6.39$, $p < .001$. Similarly, Republicans subjects spent more time looking in Both Observable ($M = 3.36$) than Punishment Observable ($M = 3.03$), $b = .34$ [.20, .47], $t = 4.83$, $p < .001$. In Study 4, among Democrats, subjects spent more time looking in Both Observable ($M = 3.41$) than Punishment Observable ($M = 3.20$), $b = .21$ [.06, .36], $t = 2.72$, $p = .007$. Similarly, Republicans subjects spent more time looking in Both Observable ($M = 3.11$) than Punishment Observable ($M = 2.71$), $b = .39$ [.24, .54], $t = 5.02$, $p < .001$.

Next, we use our new definition of looking to redefine punishment without looking as punishing after looking for a below-median amount of time. In doing so, we compute the median (and thus define subjects as being above vs. below the median) separately for Democrats and Republicans. Then, we report the effects of making (i) punishment observable and (ii) looking observable, on punishment without looking.

In Study 3, among Democrats, subjects punished without looking at comparable rates in Punishment Observable (.10) and Nothing Observable (.10), $b = .002$ [-.03, .04], $t = .12$, $p = .907$, and comparable rates in Both Observable (.08) and Punishment Observable (.10), $b = -.02$ [-.06, .01], $t = -1.42$, $p = .157$. Among Republicans, subjects punished without looking at higher rates in Punishment Observable (.14) than Nothing Observable (.10), $b = .05$ [.01, .08], $t = 2.56$, $p = .011$, and lower rates in Both Observable (.10) than Punishment Observable (.14), $b = -.04$ [-.08, -.01], $t = -2.28$, $p = .023$.

In Study 4, among Democrats, subjects punished without looking at higher rates in Punishment Observable (.14) than Nothing Observable (.08), $b = .06$ [.02, .09], $t = 3.26$, $p = .001$, and comparable rates in Both Observable (.11) and Punishment Observable (.14), $b = -.03$ [-.06, .01], $t = -1.37$, $p = .170$. Similarly, Republican subjects punished without looking at higher rates in Punishment Observable (.13) than Nothing Observable (.06), $b = .07$ [.03, .11], $t = 3.38$, $p = .001$, and comparable rates in Both Observable (.12) and Punishment Observable (.13), $b = -.01$ [-.06, .04], $t = -.45$, $p = .651$.

4.5.2 Number of articles

Next, we define looking as the continuous number of opposing perspective articles that subjects clicked the link for. When defining looking this way, in Study 3, we find that Democrats read a larger number of articles in Both Observable ($M = .67$) than Punishment Observable ($M = .44$), $b = .24$ [.16, .31], $t = 5.96$, $p < .001$. Similarly, Republicans read a larger number of articles in Both Observable ($M = .61$) than Punishment Observable ($M = .38$), $b = .23$ [.15, .32], $t = 5.23$, $p < .001$. In Study 4, among Democrats, subjects read a larger number of articles in Both Observable ($M = .67$) than Punishment Observable ($M = .54$), $b = .13$ [.05, .21], $t = 3.12$, $p = .002$. Similarly, Republicans subjects read a larger number of articles in Both Observable ($M = .44$) than Punishment Observable ($M = .20$), $b = .24$ [.15, .33], $t = 5.12$, $p < .001$.

We do not use this definition of looking to redefine punishment without looking. This reflects that, in all studies, less than half of subjects read even one article; thus, if we were to mirror our above approach and redefine punishment without looking as punishing after looking at a below-median number of articles, we would wind up with the same definition of punishment without looking that we already used in our primary analyses.

5. Discussion of preregistration deviations

All studies were pre-registered (Study 1a: https://aspredicted.org/blind.php?x=RB5_VT1; Study 1b: https://aspredicted.org/522_B9Z; Study 2a: https://aspredicted.org/43C_4DD; Study 2b: https://aspredicted.org/1B8_B91; Study 3a: https://aspredicted.org/blind.php?x=WM4_C5K; Study 3b: https://aspredicted.org/XX6_24X; Study 4a initial pre-registration: https://aspredicted.org/2HX_QQJ; Study 4a pre-registration amendment: https://aspredicted.org/JLJ_QPY; Study 4b: https://aspredicted.org/XRC_RY3). As described in the main text, our analyses closely to our preregistered analysis plans with some minor deviations. Here, we describe these deviations.

5.1 Studies 1-2

We begin by describing deviations in our analyses of our Evaluator studies (i.e., Studies 1-2.) We did not deviate from our pre-registrations for Studies 2a-b; thus, this section outlines deviations in our analyses of Studies 1a-b.

First, our Study 1a-b preregistrations describe our between-subject analyses (of evaluations of punishers who did vs. did not look) as primary analyses. However, for reasons of brevity and because these analyses produce results that are similar to our within-subject analyses but afford less statistical power, we report them only in the SI and not in the main text.

Second, our preregistrations describe our analyses of money sent in the Dictator Game as primary. In contrast, analyses of all other dependent variables (i.e., ratings of overall positivity, fairness, competence, and loyalty) are described in our pre-registrations as secondary analyses. Yet our main text does not preferentially focus on money sent. Instead, we report analyses of

money sent and *also* ratings of overall positivity, fairness, competence, and loyalty, and specifically plot positivity ratings in main text Figure 1.

To explain this decision, we begin by noting that we saw money sent in the Dictator Game and ratings of overall positivity and as "global" evaluation variables (i.e., as variables that reflected Evaluators' overall impressions of Actors). In contrast, we saw ratings of fairness, competence, and loyalty as more specific evaluation variables. Originally, we planned to privilege money sent as our "primary" global evaluation variable. However, upon seeing our data, we noticed that many Evaluators chose either to share none or to exactly half of their endowment, and almost no Evaluators shared more than half of their endowment. This distributional feature likely reflects that money shared in the Dictator Game tapped both (i) the extent to which the Evaluator had a positive impression of the Actor *and* (ii) the extent to which the Evaluator was selfish vs. generous, or committed to avoiding inequality, etc. For example, Evaluators who were motivated to maximize their own payoffs may have never shared any money with any Actors, regardless of how positively they regarded them. And Evaluators who were committed to fairness may have always shared half of their money with all Actors, regardless of how positively they regarded them.

In contrast, ratings of overall positivity were more continuously distributed, perhaps reflecting that this global evaluation variable was a "purer" reflection of positive regard for the Actor. Thus, we ultimately felt that overall positivity was the more informative global evaluation variable. Moreover, we also ultimately felt that analyses of perceived fairness, competence, and loyalty provided critical insight into *why* Evaluators formed the global evaluations of Actors that they did. For these reasons, we report analyses of all DVs in our main text, and plot overall positivity in Figure 1 (while reproducing this figure with sharing in SI Figure S1). Furthermore, in Studies 2a-b, we pre-registered positivity ratings our as our primary DV.

Third, our pre-registrations describe our analyses of Evaluations of punishers who do vs. do not look as primary, but our analyses of Evaluations of punishers vs. non-punishers (in the absence of looking information) as secondary. However, our main text prominently features both sets of analyses. This decision reflects that, while our primary theoretical focus *was* on evaluations of punishers who did vs. did not look, we felt that understanding whether punishment was evaluated positively *in general* was critical for interpreting Evaluators' preferences for punishment with vs. without looking.

Fourth, we report mediation analyses that were not pre-registered. While exploratory, we felt that these analyses were informative (and pre-registered them in Studies 2a-b).

5.3 Studies 3-4

Next, we describe deviations in our analyses of our Actor studies (i.e., Studies 3-4). First, our Study 3-4 preregistrations describe as primary two analyses that we ultimately report only in the SI (and not in our main text): (i) analyses of the effect of making punishment observable on looking, and (ii) analyses of the effect of making looking observable on punishment. As noted in the main text, we ultimately saw these analyses as less central to our theoretical questions; we therefore felt that they would distract from the flow of the main text and obfuscate the relationship between our analyses and the hypotheses we sought to test.

Second, for Study 4 but not Study 3, we preregistered a plan to report secondary analyses that used alternative definitions of looking. However, for completeness, in this SI we report these secondary analyses for both Study 4 *and* Study 3.

6. Appendix: Experimental stimuli

Here, we describe and illustrate with screenshots our experimental stimuli for all studies. Additionally, on OSF (https://osf.io/3es2k/?view_only=e272c31aa7304eca841f084d582187fb), we provide .qsf and PDF export files of the Qualtrics surveys used for each study, as well as PDFs containing the full texts of all opposing perspective articles (that subjects in Studies 3-4 viewed if they clicked the relevant links).

6.1 Studies 1a-b

After reporting their Prolific IDs and providing informed consent, subjects began by reporting their age, gender, and political party affiliation, and answering two attention check questions:

Age:

Which gender do you identify with more closely?

- Male
- Female
- Non-binary / other identity

Do you think of yourself as more of a Democrat or a Republican?

If you instead consider yourself an Independent, please indicate whether, as of today, you *lean* more Democratic or Republican.

Democrat	Republican
○	○

Thank you. Please **carefully** read the following story about a woman named Sarah.

Sarah works at a local grocery store. At the store, Sarah's job is to serve as the cashier. Normally, Sarah works Monday-Friday but does not work weekends. However, last week Sarah's coworker Ben asked her to cover his Saturday shift. So this Saturday, Sarah has to work a 7-hour shift.

[Page break]

What is Sarah's job at the grocery store?

- ◯ Manager
- ◯ Cashier
- ◯ Stocker
- ◯ Customer service
- ◯ It was not specified in the story

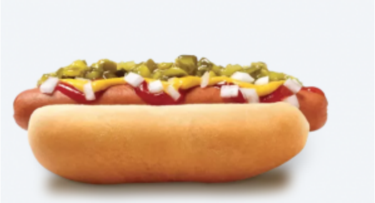

The meal pictured above consists of multiple food items. From the options below, please select the food that is probably not one of these food items.

- ◯ ketchup
- ◯ onion
- ◯ mustard
- ◯ bread
- ◯ olives
- ◯ relish
- ◯ hotdog

Next, we introduced the Dictator Game (described as the "Sharing Game"), informed subjects that they would be interacting with another participant (described as "Player 2") who shares their political party, and presented a set of comprehension questions about the game:

In this study, you will also participate in an interactive game with another participant.

The game is called the Sharing Game, and it has two players: Player 1 and Player 2. **You will be Player 1.**

Another participant will be Player 2. Like you, this participant ALSO identifies as a **Democrat**. And, just like we just told you that Player 2 is a Democrat, we will also tell Player 2 that you are a Democrat.

In the Sharing Game, you (Player 1) start with 50 cents. You then choose how many cents, if any, to share with Player 2.

Please answer the following questions, to make sure you understand the Sharing Game.

Imagine that you are deciding how much to share with Player 2.

Which decision will result in **you** earning the most money?

- ○ You deciding to share 0 cents
- ○ You deciding to share 25 cents
- ○ You deciding to share 50 cents

Imagine that you are deciding how much to share with Player 2.

Which decision will result in **Player 2** earning the most money?

- ○ You deciding to share 0 cents
- ○ You deciding to share 25 cents
- ○ You deciding to share 50 cents

What political affiliation is Player 2?

- ○ Player 2 is a Republican
- ○ Player 2 is a Democrat

Note: the above screenshot shows the screen for Democrats in Study 1a. For Republicans in Study 1b, references to Democrats were replaced with references to Republicans.

Next, we explained that Player 2 signed a petition, and described the petition. Below we show how this looked for each of the three petitions. Recall that in Study 1a, Democrats were randomly assigned to either the Moore or Negy petition; in Study 1b, Republicans were always assigned to the Amazon petition.

Democrats, Moore Petition:

On this page, we would like you to tell you a bit more about Player 2.

We already recruited Player 2 to complete a study, which had an additional component.

In addition to participating in the Sharing Game, Player 2 also had the opportunity to sign a petition, hosted on Change.org.

The petition calls for the LAPD to fire Chief Michael Moore. It argues that Chief Moore should be fired following comments he made that blamed protestors for George Floyd's death.

On the next page, we will provide you with the full text of the petition and ask you to read it.

We also showed this full text to Player 2, who then decided whether or not to help get Chief Moore fired by signing the petition.

Democrats, Negy Petition:

On this page, we would like you to tell you a bit more about Player 2.

We already recruited Player 2 to complete a study, which had an additional component.

In addition to participating in the Sharing Game, Player 2 also had the opportunity to sign a petition, hosted on Change.org.

The petition calls for the University of Central Florida to fire professor Charles Negy. The petition alleges that professor Negy should be fired on the basis of abhorrent racist comments he has made on his personal Twitter account.

On the next page, we will provide you with the full text of the petition and ask you to read it.

We also showed this full text to Player 2, who then decided whether or not to help get professor Negy fired by signing the petition.

Republicans, Amazon Petition:

On this page, we would like you to tell you a bit more about Player 2.

We already recruited Player 2 to complete a study, which had an additional component.

In addition to participating in the Sharing Game, Player 2 also had the opportunity to sign a petition, hosted on Change.org.

The petition calls for the remove of “Blue Lives Murder” merchandise from Amazon. It alleges that the merchandise is hateful and must be immediately removed.

On the next page, we will provide you with the full text of the petition and ask you to read it.

We also showed this full text to Player 2, who then decided whether or not to help remove “Blue Lives Murder” merchandise from Amazon by signing the petition.

Next, we presented a screenshot of the petition.

Democrats, Moore Petition:

Below is a screenshot of the petition that we showed to Player 2. Over 300,000 people have already signed the petition, as highlighted in the screenshot below.

Again, the petition calls for the LAPD to fire Chief Michael Moore. It argues that Chief Moore should be fired following comments he made that blamed protestors for George Floyd's death.

On this screen, please read through the petition.

Fire LAPD Chief Michael Moore

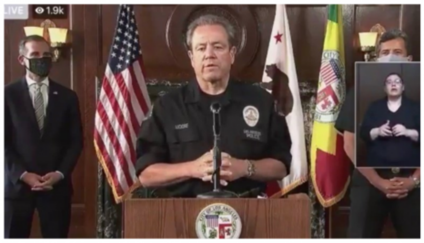

sarah martin started this petition to LA County

LAPD is a corrupt department who claims the **peaceful** protests all around Los Angeles are civil unrests and criminal acts. This man should be fired.

On June 1st , Moore stated that George Floyd's death is on protestors and looters hands. He is belittling the struggles and ways of the Black Lives Matter movement. This is only the shadow of an underlying racist and discriminating department.

Let LAPD Police Chief Michael Moore know how you feel about his blaming protestors for George Floyd's death:

301,339 have signed. Let's get to 500,000!

Ana Júlia Maciel signed 26 minutes ago

Grace Boyle signed 42 minutes ago

First name

Last name

Email

Country

City

Display my name and comment on this petition

I'm not a robot

reCAPTCHA
Privacy - Terms

Sign this petition

By signing, you accept Change.org's Terms of Service and Privacy Policy, and agree to receive occasional emails about campaigns on Change.org. You can unsubscribe at any time.

Democrats, Negy Petition:

Below is a screenshot of the petition that we showed to Player 2. Over 30,000 people have already signed the petition, as highlighted in the screenshot below.

Again, the petition calls for the University of Central Florida to fire professor Charles Negy. It alleges that professor Negy should be fired on the basis of abhorrent racist comments he has made on his personal Twitter account.

On this screen, please read through the petition.

UCF: Fire Psychology Professor Charles Negy

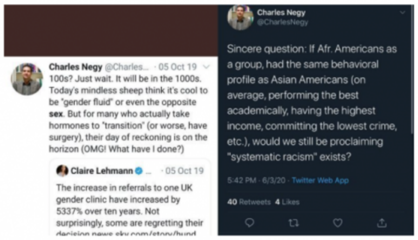

30,698 have signed. Let's get to 35,000!

Gabi Guisbert signed 8 minutes ago

Micah Baxter-Miller signed 9 minutes ago

First name

Last name

Email

Display my name and comment on this petition

Sign this petition

By signing, you accept Change.org's Terms of Service and Privacy Policy, and agree to receive occasional emails about campaigns on Change.org. You can unsubscribe at any time.

Eva Oliveri started this petition to University of Central Florida and 3 others

We are calling on the University of Central Florida to dismiss psychology professor Charles Negy due to abhorrent racist comments he has made and continues to make on his personal Twitter account. In addition to racism, Negy has engaged in perverse transphobia and sexism on his account, which is just as reprehensible. While he has a right to free speech, he does not have a right to dehumanize students of color and other minority groups, which is a regular occurance in his classroom. By allowing him to continue in his position, UCF would simply be empowering another cog in the machine of systemic racism.

UCF is a diverse, welcoming campus of 60,000 students from all across the country and the world. Our diversity is our strength, and we should have faculty who understand and promote that - not the opposite. College is a place to learn and be exposed to new ideas, but for so many Black students, racism is not just an idea, but something they have experienced for their entire life, and the same goes for the LGBT community with trans and homophobia. They deserve better, and so does the entire campus community.

I once again want to reiterate my love for this university. We are a community which always has each other's back. As a student coming from a place of privilege, this is me having the backs of my fellow Knights.

Please dismiss Professor Negy for the good of the entire campus community.

Republicans, Amazon Petition:

Below is a screenshot of the petition that we showed to Player 2. Over 115,000 people have already signed the petition, as highlighted in the screenshot below.

Again, the petition calls for the removal of "Blue Lives Murder" merchandise from Amazon. It argues that the merchandise is hateful and must be immediately removed.

On this screen, please read through the petition.

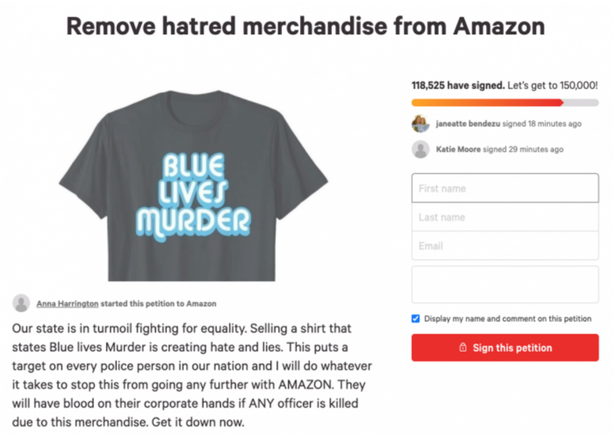

Next, we explained to subjects that Player 2 had the chance to consider opposing perspectives.

Democrats, Moore petition:

After viewing this petition, Player 2 decided whether or not to sign it.

Additionally, we gave Player 2 an opportunity to take some time—if Player 2 wanted it—to consider OPPOSING perspectives before deciding whether to sign. It was completely up to Player 2 whether to use this time, and how.

For Player 2's convenience, we provided links to some specific articles that may provide **opposing** perspectives pertaining to the petition. For example, we provided a link to the below article published by the LA Times describing how elected politicians in Los Angeles, including the mayor, continue to support Police Chief Moore.

Player 2 also had the option of taking time to search the Internet for other opposing perspectives.

And, importantly, Player 2 also had the option NOT to read any opposing perspectives before deciding whether or not to sign the petition.

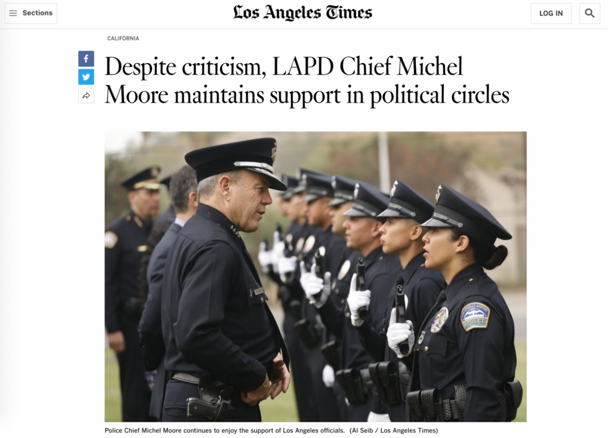
Sections

Los Angeles Times

LOG IN

CALIFORNIA

Despite criticism, LAPD Chief Michel Moore maintains support in political circles

Police Chief Michel Moore continues to enjoy the support of Los Angeles officials. (Al Seib / Los Angeles Times)

Democrats, Negy petition:

After viewing this petition, Player 2 decided whether or not to sign it.

Additionally, we gave Player 2 an opportunity to take some time—if Player 2 wanted it—to consider OPPOSING perspectives before deciding whether to sign. It was completely up to Player 2 whether to use this time, and how.

For Player 2's convenience, we provided links to some specific articles that may provide **opposing** perspectives pertaining to the petition. For example, we provided a link to the below article, published by the Orlando Sentinel, describing professor Negy's claim that he is the subject of a "witch hunt".

Player 2 also had the option of taking time to search the Internet for other opposing perspectives.

And, importantly, Player 2 also had the option NOT to read any opposing perspectives before deciding whether or not to sign the petition.

UCF professor behind tweets deemed racist says he is subject of 'witch hunt'

By Annie Martin Orlando Sentinel (TNS) Jun 18, 2020

Republicans, Amazon Petition:

After viewing this petition, Player 2 decided whether or not to sign it.

Additionally, **we gave Player 2 an opportunity to take some time—if Player 2 wanted it—to consider OPPOSING perspectives before deciding whether to sign.** It was completely up to Player 2 whether to use this time, and how.

For Player 2's convenience, we provided links to some specific articles that may provide **opposing** perspectives pertaining to the petition. For example, we provided a link to the below article, published by a local Houston news outlet, reporting on the perspective of an activist who does not think "Blue Lives Murder" merchandise constitutes hate speech.

Player 2 also had the option of taking time to search the Internet for other opposing perspectives.

And, importantly, Player 2 also had the option NOT to read any opposing perspectives before deciding whether or not to sign the petition.

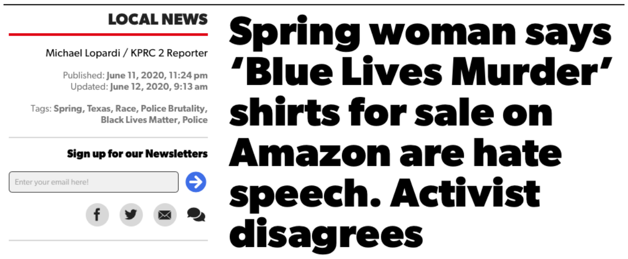
LOCAL NEWS

Michael Lopardi / KPRC 2 Reporter

Published: June 11, 2020, 11:24 pm
Updated: June 12, 2020, 9:13 am

Tags: Spring, Texas, Race, Police Brutality, Black Lives Matter, Police

Sign up for our Newsletters

Enter your email here!

Spring woman says 'Blue Lives Murder' shirts for sale on Amazon are hate speech. Activist disagrees

Next, we explained that Player 2s varied with respect to the amount of time they spent looking:

Importantly, participants assigned to the role of Player 2 varied in the amount of time they spent considering opposing perspectives before deciding whether or not to sign the petition.

Some Player 2s spent **no or very little time** considering opposing perspectives before deciding whether or not to sign the petition.

In contrast, other Player 2s spent **a lot of time** considering opposing perspectives before deciding whether or not to sign the petition.

Next, we collected our dependent variables, for a set of different Player 2s with different behavioral profiles (i.e., a set of Player 2s about whom we provided different information about their punishment and looking behavior).

The first two Player 2s that subjects evaluated were punishers who did vs. did not look. We manipulated, between subjects, the order in which these Player 2s were presented. After the first but before the second was presented, subjects saw a transition screen.

This was the screen subjects used to evaluate a punisher who did *not* look:

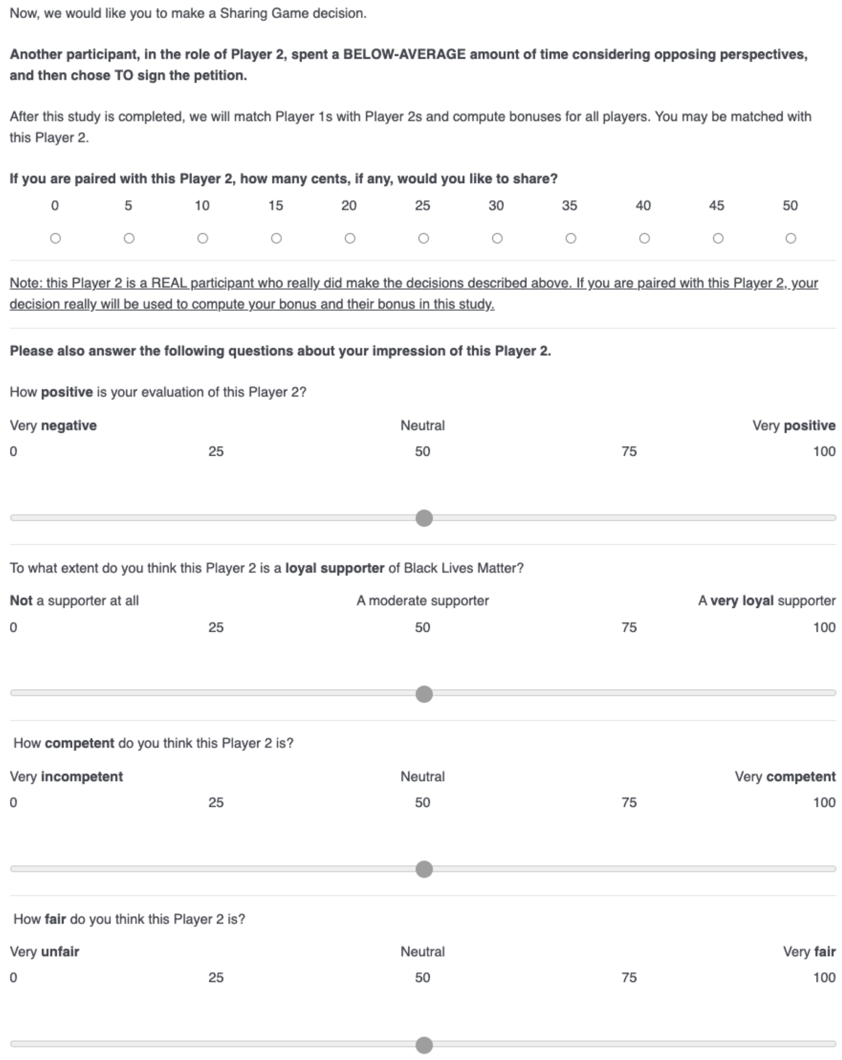

Now, we would like you to make a Sharing Game decision.

Another participant, in the role of Player 2, spent a BELOW-AVERAGE amount of time considering opposing perspectives, and then chose TO sign the petition.

After this study is completed, we will match Player 1s with Player 2s and compute bonuses for all players. You may be matched with this Player 2.

If you are paired with this Player 2, how many cents, if any, would you like to share?

0	5	10	15	20	25	30	35	40	45	50
○	○	○	○	○	○	○	○	○	○	○

Note: this Player 2 is a REAL participant who really did make the decisions described above. If you are paired with this Player 2, your decision really will be used to compute your bonus and their bonus in this study.

Please also answer the following questions about your impression of this Player 2.

How **positive** is your evaluation of this Player 2?

Very **negative** | Neutral | Very **positive**

0 25 50 75 100

To what extent do you think this Player 2 is a **loyal supporter** of Black Lives Matter?

Not a supporter at all | A moderate supporter | A **very loyal** supporter

0 25 50 75 100

How **competent** do you think this Player 2 is?

Very **incompetent** | Neutral | Very **competent**

0 25 50 75 100

How **fair** do you think this Player 2 is?

Very **unfair** | Neutral | Very **fair**

0 25 50 75 100

Note: the above screenshot shows the screen for Democrats in Study 1a, where we measured loyalty towards Black Lives Matter; for Republicans in Study 1b, we instead measured loyalty towards Blue Lives Matter. We also randomized between-subjects the order in which we presented the questions about how competent, fair, and loyal Player 2 was; subjects were randomly assigned to one order and then that order was consistently applied across all Player 2s that they evaluated.

The screen that subjects used to evaluate a punisher who *did* look was identical, except that we replaced “below-average” with “above-average” in the second sentence on the screen.

This was the transition screen that subjects saw (between evaluating a punisher who did vs. did not look):

Thank you for making that decision.

On the subsequent screens, you will make a series of decisions about how much to share with OTHER potential Player 2s that you could be paired with.

These Player 2s are all REAL participants, who, like you, identify as Democrats. They all had the opportunity to sign the petition about Professor Charles Negy. And they all chose how long to spend considering opposing perspectives before deciding whether or not to sign the petition. However, these Player 2s varied in terms of the decisions that they made in the study.

On the subsequent screens, please decide how much you would like to share with each of them, if you are ultimately paired with them.

Note: the above screenshot shows screen for Democrats who were assigned to the Negy petition; the text was adapted to reference the correct party and petition for all subjects.

The next two Player 2s that subjects evaluated were non-punishers who did vs. did not look. Subjects who evaluated a punisher who *did* look before a punisher who did *not* look correspondingly evaluated a non-punisher who *did* look before evaluating a non-punisher who did *not* look, and vice versa. The screens that subjects used to evaluate non-punishers were identical to the screens they used to evaluate punishers, except that we replaced “chose TO sign the petition” with “chose NOT to sign the petition”.

The next two Player 2s that subjects evaluated were punishers and non-punishers, with no information provided about looking. Before presenting these two Player 2 profiles, we showed subjects this transition screen:

Thank you.

On the next screens, you will make two more sharing game decisions about Player 2s that you might be matched with.

For these decisions, we will tell you whether or not Player 2 decided to sign the petition.

However, we will NOT tell you anything about how long Player 2 spent considering opposing perspectives before making this decision.

The screens that subjects used to evaluate Player 2s without looking information were identical to the screens they used to evaluate Player 2s *with* looking information, except that we did not provide information about looking. So, the non-punisher screen began like this:

Now, we would like you to make another Sharing Game decision.

Another participant, in the role of Player 2, chose NOT to sign the petition.

And punisher screen began identically, except that we replaced “chose NOT to” with “chose TO”.

Next, subjects evaluated a Player 2 about whom they had no information:

Thank you for making these decisions.

Finally, how many cents would you like to share with Player 2, if given no information about Player 2's decisions?

0	5	10	15	20	25	30	35	40	45	50
○	○	○	○	○	○	○	○	○	○	○

Note: this decision may really be used to compute your bonus and the bonus of a Player 2 in this study.

Given no information about Player 2's decisions, how **positive** is your impression of Player 2?

Very **negative** — Neutral — Very **positive**

0 25 50 75 100

Given no information about Player 2's decisions, to what extent do you think Player 2 is a **loyal supporter** of Black Lives Matter?

Not a supporter at all — A moderate supporter — A **very loyal** supporter

0 25 50 75 100

Given no information about Player 2's decisions, how **competent** do you think Player 2 is?

Very **incompetent** — Neutral — Very **competent**

0 25 50 75 100

Given no information about Player 2's decisions, how **fair** do you think Player 2 is?

Very **unfair** — Neutral — Very **fair**

0 25 50 75 100

Finally, subjects completed the following post-experimental survey:

Thank you for making those decisions.

Now, we would like you to **provide your own opinion about the petition.** We have included a screenshot of the petition below, for your reference.

Please rate your agreement with the following statement: **"I strongly support the petition and the underlying cause behind it."**

1 - Strongly disagree	2	3 - Somewhat disagree	4	5 - Neither agree nor disagree	6	7 - Somewhat agree	8	9 - Strongly agree
○	○	○	○	○	○	○	○	○

[Below this question, we re-printed the screenshot of the petition]

[Page break]

Please describe how you made your choices in this study.

[Page break]

Is there anything you'd like to share with us about your impression of this study?

[Page break]

To what extent have you previously participated in other studies like to this one?

1 - Nothing like this scenario	2	3 - Something like this scenario	4	5 - Exactly this scenario
○	○	○	○	○

To what extent do you believe that the petition is real?

1 - Very skeptical that it is real	2	3	4	5	6	7 - Very confident that it is real
○	○	○	○	○	○	○

To what extent do you believe that you really will be matched with a real Player 2?

1 - Very skeptical that I will be matched with a real Player 2	2	3	4	5	6	7 - Very confident that I will be matched with a real Player 2
○	○	○	○	○	○	○

[Page break]

Please choose the category that describes the total amount of income you earned in 2020. Consider all forms of income, including salaries, tips, interest and dividend payments, scholarship support, student loans, parental support, social security, alimony, and child support, and others.

- ○ Under $5,000
- ○ $5,000-$10,000
- ○ $10,001-$15,000
- ○ $15,001-$25,000
- ○ $25,001-$35,000
- ○ $35,001-$50,000
- ○ $50,001-$65,000
- ○ $65,001-$80,000
- ○ $80,001-$100,000
- ○ Over $100,000

Please specify your race. (*Choose one or more categories*)

- ☐ White/Caucasian (Anglo/Euro) American
- ☐ Black or African American
- ☐ Asian or Asian American
- ☐ American Indian or Alaska Native
- ☐ Native Hawaiian or other Pacific Islander
- ☐ Hispanic/Latino
- ☐ Multicultural

You indicated that you lean Democrat. Which is more true of you?

- ○ I consider myself a strong Democrat, who strongly supports the party
- ○ I consider myself a weak Democrat, who only leans towards the party

On the below scale, how **strong** of a Democrat do you consider yourself?

1 - Not very strong Democrat	2	3	4 - Moderately strong Democrat	5	6	7 - Very strong Democrat
○	○	○	○	○	○	○

In general, how conservative or liberal do you consider yourself to be?

1 - Very conservative	2	3	4	5 - Neither liberal nor conservative	6	7	8	9 - Very liberal
○	○	○	○	○	○	○	○	○

When it comes to SOCIAL policy, how conservative or liberal do you consider yourself to be?

1 - Very conservative	2	3	4	5 - Neither liberal nor conservative	6	7	8	9 - Very liberal
○	○	○	○	○	○	○	○	○

When it comes to ECONOMIC policy, how conservative or liberal do you consider yourself to be?

1 - Very conservative	2	3	4	5 - Neither liberal nor conservative	6	7	8	9 - Very liberal
○	○	○	○	○	○	○	○	○

To what extent do you support Black Lives Matter?

1 - Strongly oppose	2	3	4	5 - Neither support nor oppose	6	7	8	9 - Strongly support
○	○	○	○	○	○	○	○	○

Note: the above screenshot shows the screen for Democrats in Study 1a, where we measured support for Black Lives Matter; for Republicans in Study 1b, we referenced Republicans (rather than Democrats) and measured support for Blue Lives Matter ("To what extent do you support "Blue Lives Matter" (a countermovement, started in response to Black Lives Matter, advocating that those who are prosecuted and convicted of killing law enforcement officers should be sentenced under hate crime statutes)?").

6.2 Studies 2a-b

Studies 2a-b were extremely similar to Studies 1a-b. Here, we show screenshots that highlight the few differences.

First, the screen introducing the Dictator Game was identical to Studies 1a-b for all Study 2 conditions except the "other participant" treatment from Study 2a. In that treatment, the screen looked different because we did not say that Player 2 was a co-partisan, but rather described Player 2 merely as "another participant" (and asked one fewer comprehension questions):

In this study, you will also participate in an interactive game with another participant.

The game is called the Sharing Game, and it has two players: Player 1 and Player 2. **You will be Player 1.**

Player 2 is another participant.

In the Sharing Game, you (Player 1) start with 50 cents. You then choose how many cents, if any, to share with Player 2.

Please answer the following questions, to make sure you understand the Sharing Game.

Imagine that you are deciding how much to share with Player 2.

Which decision will result in **you** earning the most money?

- You deciding to share 0 cents
- You deciding to share 25 cents
- You deciding to share 50 cents

Imagine that you are deciding how much to share with Player 2.

Which decision will result in **Player 2** earning the most money?

- You deciding to share 0 cents
- You deciding to share 25 cents
- You deciding to share 50 cents

Second, before subjects began evaluating Player 2, we showed a screen that (i) provided subjects in the treatments that "added information" with some additional information about the Player 2 they were paired with, and (ii) asked all subjects to write a paragraph reflecting on their initial impression of Player 2.

This was the relevant screen in the baseline conditions:

On the following screens, you will answer some questions about Player 2. Remember, Player 2 is a REAL other participant, who identifies as a Democrat.

In light of all of the information that you have received about Player 2, what is initial your impression of them? Please write a few sentences in the box below.

This was the screen in the “other participant” treatment from Study 2a:

On the following screens, you will answer some questions about Player 2. Remember, Player 2 is a REAL other participant.

In light of all of the information that you have received about Player 2, what is initial your impression of them? Please write a few sentences in the box below.

This was the screen in the “Democrat + Conflict of Interest (Privacy)” treatment from Study 2a:

On the following screens, you will answer some questions about Player 2. Remember, Player 2 is a REAL other participant.

First, however, we'd like to provide some additional information about the Player 2 you have been paired with. In particular, below we have provided a screenshot of some questions that we asked Player 2, and the answers that Player 2 provided.

On this page, we have printed all instructions for you in GREEN, to help you distinguish the screenshot from the instructions for you.

Please carefully consider the questions we asked Player 2, and the answers they provided, before advancing the screen.

Do you think of yourself as more of a Democrat or a Republican?

If instead you consider yourself an Independent, please indicate whether, as of today, you *lean* more Democratic or Republican.

Democrat	Republican
◉	○

Before you decide whether to sign the petition, we'd like you to reflect on whether there might be any reason that signing the petition would **not be in your personal self-interest**.

In other words, do you have any "conflict of interest" with the petition? **Is there any reason it would be bad for YOU for this petition to receive support?**

As a reminder, the petition calls for the LAPD to fire Chief Michael Moore. It argues that Chief Moore should be fired following comments he made that blamed protestors for George Floyd's death.

In the box below, please describe any reason(s) you can think of that signing the petition would not be in your personal self-interest.

Please explain in at least 1-2 sentences.

Signing the petition might make some of my personal information public. This could lead to me receiving spam. This could become more severe as the petition receives more support and attention.

>>

In light of all of the information that you have received about Player 2, what is initial your impression of them? Please write a few sentences in the box below.

>>

In the other treatments that added information, the screen was identical to the screen shown above, except that we featured a different Player 2 response screenshot.

This was the Player 2 response screenshot in the "Democrat + Ignore injustice" treatment from Study 2a:

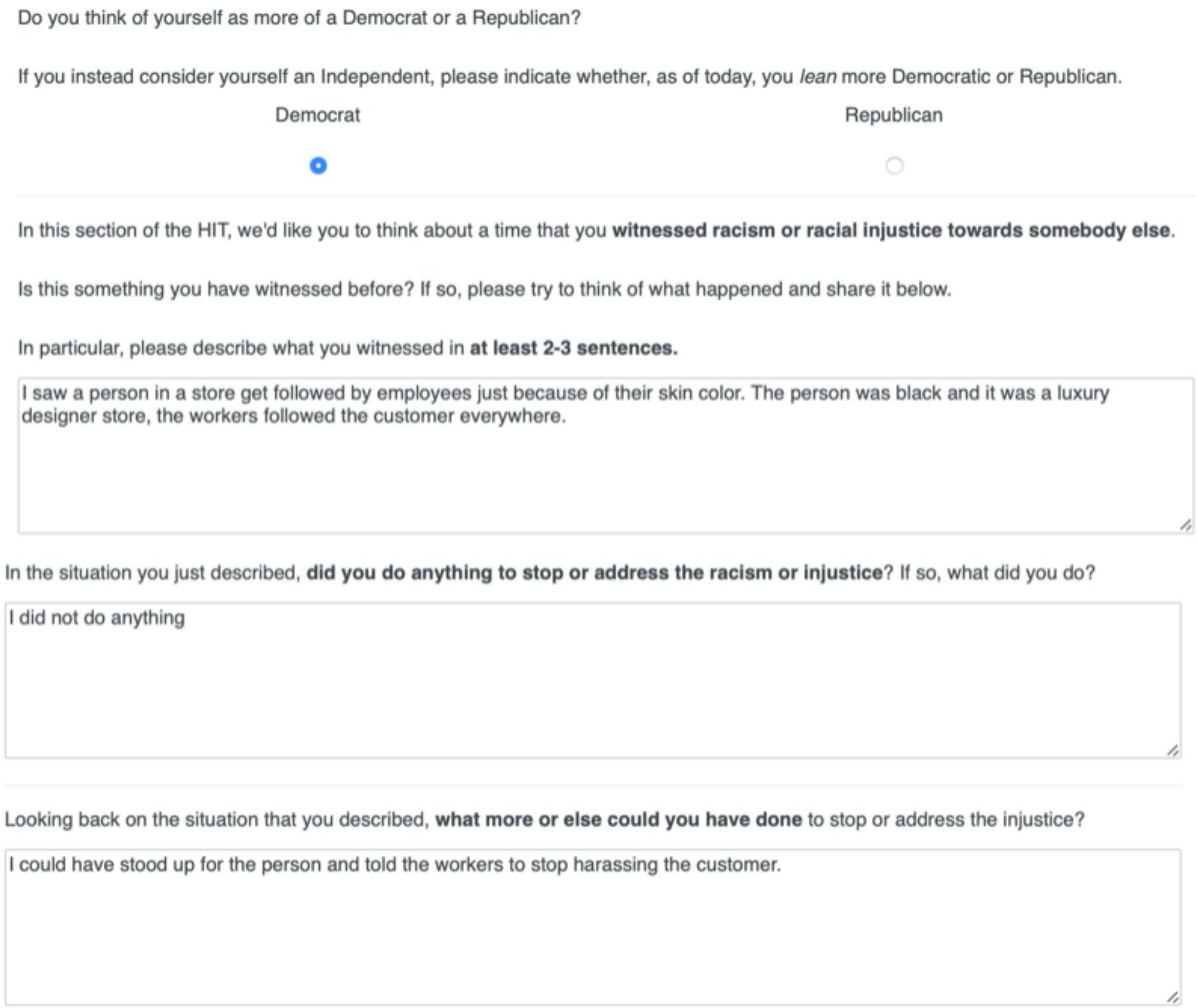
Do you think of yourself as more of a Democrat or a Republican?

If you instead consider yourself an Independent, please indicate whether, as of today, you *lean* more Democratic or Republican.

Democrat (selected) Republican

In this section of the HIT, we'd like you to think about a time that you **witnessed racism or racial injustice towards somebody else**.

Is this something you have witnessed before? If so, please try to think of what happened and share it below.

In particular, please describe what you witnessed in **at least 2-3 sentences.**

I saw a person in a store get followed by employees just because of their skin color. The person was black and it was a luxury designer store, the workers followed the customer everywhere.

In the situation you just described, **did you do anything to stop or address the racism or injustice**? If so, what did you do?

I did not do anything

Looking back on the situation that you described, **what more or else could you have done** to stop or address the injustice?

I could have stood up for the person and told the workers to stop harassing the customer.

This was the screenshot in the "Democrat + Conflict of interest (Police)" treatment from Study 2a:

Do you think of yourself as more of a Democrat or a Republican?

If instead you consider yourself an Independent, please indicate whether, as of today, you *lean* more Democratic or Republican.

Democrat (selected) Republican

Before you decide whether to sign the petition, we'd like you to reflect on whether there might be any reason that signing the petition would **not be in your personal self-interest**.

In other words, do you have any "conflict of interest" with the petition? **Is there any reason it would be bad for YOU for this petition to receive support?**

As a reminder, the petition calls for the LAPD to fire Chief Michael Moore. It argues that Chief Moore should be fired following comments he made that blamed protestors for George Floyd's death.

In the box below, please describe any reason(s) you can think of that signing the petition would not be in your personal self-interest.

Please explain in at least 1-2 sentences.

I have an uncle who was a police officer.

This was the screenshot in the "Democrat + Previously Independent" treatment from Study 2a:

Do you think of yourself as more of a Democrat or a Republican?

If you instead consider yourself an Independent, please indicate whether, as of today, you *lean* more Democratic or Republican.

Democrat (selected) Republican

Prior to the way you currently identify politically, which political party did you consider yourself to be a member of (if applicable)?

Democrat Republican Independent/Other (please specify) (selected)

Please describe, in a few sentences, the history of the way that you have identified politically over time.

If you have not always identified politically the way that you do now, please comment on how you used to identify, and what prompted the change. What lead you to your current political identity?

I used to praise Trump, and then COVID hit, and Trump showed his true colors, and I became a Democrat after that.

How many of the last four presidential or midterm elections (2020, 2018, 2016, 2014) have you cast a vote in?

0 1 (selected) 2 3 4

This was the screenshot in the "Democrat + Responded to injustice" treatment from Study 2b:

Do you think of yourself as more of a Democrat or a Republican?

If you instead consider yourself an Independent, please indicate whether, as of today, you *lean* more Democratic or Republican.

Democrat (selected) | Republican

In this section of the HIT, we'd like you to think about a time that you witnessed racism or racial injustice towards somebody else.

Is this something you have witnessed before? If so, please try to think of what happened and share it below.

In particular, please describe what you witnessed in at least 2-3 sentences.

I have witnessed racism in my former organization where coworkers of color or anyone that wasn't white didn't get the same opportunities white workers got. They would get little to no promotions and less salary wages

In the situation you just described, did you do anything to stop or address the racism or injustice? If so, what did you do?

Yes I stood up against my former employer and encouraged other colleagues to do so as wel

After subjects reflected on their initial impression of Player 2, we also showed one more screen (that was not included in Studies 1a-b) before subjects began evaluating Player 2. In this screen, we explained that subjects would be making multiple decisions about how much to share with Player 2, depending on how Player 2 had previously behaved. This was the relevant screen:

On the subsequent screens, you will make a series of decisions about how much to share with Player 2, given different information about how Player 2 behaved in the previous study.

Importantly, after this study is completed, we will use ONE of your decisions to compute your bonus and pay you. Specifically, we will use the decision you make that corresponds to how Player 2 ACTUALLY behaved in the previous study.

However, because you do not yet know how Player 2 actually behaved, you do not yet know which of your decisions will be used to compute your bonus. **So, all of the decisions you will make are important.**

Then, like in Studies 1a-b, we collected our dependent variables, for the same set of Player 2 behavioral profiles that we featured in Studies 1a-b. However, while Studies 1a-b described each behavioral profile as corresponding to a different Player 2, Studies 2a-b described each behavioral profile as corresponding to a different way that a single Player 2 (i.e., the Player 2 that the subject was matched with) might have behaved. Moreover, we did not show a "transition screen" after the first behavior profile (like we did in Studies 1a-b) because we had already explained upfront that subjects would be making multiple decisions.

Here is an example of a Study 2 behavioral profile screen (in this case, corresponding to Player 2 choosing to punish without looking):

Now, we would like you to make a Sharing Game decision.

We will use the decision you make on this screen if the Player 2 you are paired with...

...spent a BELOW-AVERAGE amount of time considering opposing perspectives, and then chose TO sign the petition.

If Player 2 behaved in this manner, how many cents, if any, would you like to share with them?

0	5	10	15	20	25	30	35	40	45	50
○	○	○	○	○	○	○	○	○	○	○

Note: After this study is completed, we will use ONE of your decisions. Specifically, we will use the decision you make that corresponds to how Player 2 ACTUALLY behaved. Then, we will use this decision to compute bonuses for both players. So, the decision you make on this screen may actually be used.

Please also answer the following questions about your impression of Player 2, if Player 2 spent a BELOW-AVERAGE amount of time considering opposing perspectives, and then chose TO sign the petition.

In this scenario, how **positive** is your evaluation of Player 2?

Very **negative** | Neutral | Very **positive**

0 | 25 | 50 | 75 | 100

In this scenario, how **competent** do you think Player 2 is?

Very **incompetent** | Neutral | Very **competent**

0 | 25 | 50 | 75 | 100

In this scenario, how **fair** do you think Player 2 is?

Very **unfair** | Neutral | Very **fair**

0 | 25 | 50 | 75 | 100

In this scenario, to what extent do you think Player 2 is a **loyal supporter** of Black Lives Matter?

Not a supporter at all | A moderate supporter | A **very loyal** supporter

0 | 25 | 50 | 75 | 100

6.3 Studies 3-4
After reporting their Prolific or MTurk IDs and providing informed consent, subjects began by reporting their age, gender, and political party affiliation, and answering two attention check questions; these measures were all identical to those presented at the beginning of Study 1. So, see Study 1 in this document for screenshots.

Next, we introduced the Dictator Game (described as the "Sharing Game"), informed subjects that they would be interacting with another participant (described as "Player 1") who shares their political party, and presented a set of comprehension questions about the game.

For Democrats in Study 3a, Player 1 was described as relatively less ideological:

In this HIT, you will participate in an interactive game with another MTurk worker.

The game is called the Sharing Game, and it has two players: Player 1 and Player 2. **You will be Player 2.**

Another Mturk worker will be Player 1. Like you, this worker ALSO indicated to us in a survey that they identify as a **Democrat**.

More specifically, Player 1 indicated that they identify as a weak Democrat, who only leans towards the party.

And, just like we just told you that Player 1 is a Democrat, we will also tell Player 1 that you are a Democrat.

In the Sharing Game, Player 1 starts with 50 cents. Player 1 then chooses how many cents, if any, to share with you (Player 2).

Please answer the following questions, to make sure you understand the Sharing Game.

Imagine that Player 1 is deciding how much to share with to you.

Which decision will result in **Player 1** earning the highest payoff?

- Player 1 deciding to share 0 cents
- Player 1 deciding to share 25 cents
- Player 1 deciding to share 50 cents

Imagine that Player 1 is deciding how much to share with to you.

Which decision will result in **you** earning the highest payoff?

- Player 1 deciding to share 0 cents
- Player 1 deciding to share 25 cents
- Player 1 deciding to share 50 cents

What information do you know about Player 1?

- Player 1 identifies as a Republican
- Nothing
- Player 1 identifies as a weak Democrat, who only leans towards the party

For Democrats in Study 4a, Player 1 was described as relatively more ideological. Thus, the screen instead began as follows:

> In this HIT, you will also participate in an interactive game with another MTurk worker.
>
> The game is called the Sharing Game, and it has two players: Player 1 and Player 2. **You will be Player 2.**
>
> Another Mturk worker will be Player 1. Like you, this worker ALSO indicated to us in a survey that they identify as a **Democrat**.
>
> **More specifically, Player 1 indicated that they identify as a strong Democrat who strongly supports Black Lives Matter.**
>
> And, just like we just told you that Player 1 is a Democrat, we will also tell Player 1 that you are a Democrat.
>
> In the Sharing Game, Player 1 starts with 50 cents. Player 1 then chooses how many cents, if any, to share with you (Player 2).

The comprehension questions that followed were identical to those presented in Study 3a, except that the final answer chance for last question instead read “Player 1 identifies as a strong Democrat who strongly supports Black Lives Matter”.

For Republicans in Study 3b, the screen was identical to what we showed in Study 3a, except that we referenced Republicans instead of Democrats.

For Republicans in Study 4b, the screen was identical to what we showed in Study 4a, except that we (i) referenced Republicans instead of Democrats and (ii) the sentence starting with “more specifically” instead read “More specifically, Player 1 indicated that they identify as a strong Republican who strongly supports “Blue Lives Matter" (a countermovement, started in response to Black Lives Matter, advocating that those who are prosecuted and convicted of killing law enforcement officers should be sentenced under hate crime statutes).” Furthermore, the final answer choice for last question read “Player 1 identifies as a strong Republican who strongly supports Blue Lives Matter”.

If subjects answered any comprehension questions incorrectly, they were presented with the screen involving the questions a second time, along with the text: “On the previous page, you answered one or more questions incorrectly. You MUST answer ALL questions correctly to continue on with the study. Please carefully re-read the below instructions and answer the questions again”. Here, they were not allowed to precede to the next screen until they answered all questions correctly.

Next, we told subjects that they had the opportunity to sign a petition. Below we show the text we presented for each of the three petitions. Below this text, we displayed a screenshot of the petition. These screenshots were identical to those presented in Study 1; so, see Study 1 in this document for screenshots.

Democrats, Moore petition (Study 3a):

This HIT will also have another component.

In addition to participating in the Sharing Game, **you will also have the opportunity to sign a petition**, hosted on Change.org. Below, we have provided a screenshot of the petition. Later in this HIT, we will provide a link to the petition. Over 300,000 people have already signed the petition, as highlighted in the screenshot below.

The petition calls for the LAPD to fire Chief Michael Moore. It argues that Chief Moore should be fired following comments he made that blamed protestors for George Floyd's death.

On this screen, please read through the petition. Then, later in this HIT, you will have the chance to decide whether you'd like to help get Chief Moore fired by signing the petition.

You can choose to sign or not to sign. **One thing you should keep in mind is that we will tell Player 1 whether or not you choose to sign.**

Democrats, Negy petition (Study 4a):

This HIT will also have another component.

In addition to participating in the Sharing Game, **you will also have the opportunity to sign a petition**, hosted on Change.org. Below, we have provided a screenshot of the petition. Later in this HIT, we will provide a link to the petition. Over 30,000 people have already signed the petition, as highlighted in the screenshot below.

The petition calls for the University of Central Florida to fire professor Charles Negy. It argues that professor Negy should be fired on the basis of abhorrent racist comments he has made on his personal Twitter account.

On this screen, please read through the petition. Then, later in this HIT, you will have the chance to decide whether you'd like to help get professor Negy fired by signing the petition.

You can choose to sign or not to sign. **One thing you should keep in mind is that we will tell Player 1 whether or not you choose to sign.**

Note: the above two screenshots show the screens from the "Punishment Observable" and "Both Observable" conditions, where punishment was observable.

Republicans, Amazon petition (Studies 3b and 4b):

This HIT will also have another component.

In addition to participating in the Sharing Game, **you will also have the opportunity to sign a petition**, hosted on Change.org. Below, we have provided a screenshot of the petition. Later in this HIT, we will provide a link to the petition. Over 115,000 people have already signed the petition, as highlighted in the screenshot below.

The petition calls for the removal of "Blue Lives Murder" merchandise from Amazon. It argues that the merchandise is hateful and must be immediately removed.

On this screen, please read through the petition. Then, later in this HIT, you will have the chance to decide whether you'd like to help remove "Blue Lives Murder" merchandise from Amazon by signing the petition.

You can choose to sign or not to sign. **One thing you should keep in mind is that your decision will be completely private: we will NOT tell Player 1 whether or not you choose to sign.**

Note: the above screenshot shows the screen from the "Nothing Observable" condition, where punishment was not observable.

Next, we told subjects that they would have a chance to consider opposing perspective articles.

Democrats, Negy Petition (Study 4a):

Additionally, **we will give you an opportunity to take some time—if you would like—to consider OPPOSING perspectives before deciding whether to sign.** It is completely up to you whether to use this time, and how.

For your convenience, we will provide you with links to some specific articles that may provide **opposing** perspectives pertaining to the petition. For example, we will provide a link to the below article, published by the Orlando Sentinel, describing professor Negy's claim that he is the subject of a "witch hunt". You can also choose to use the time to search the Internet for other opposing perspectives.

Importantly, you can also choose NOT to read any opposing perspectives and instead move forward with the survey.

You can take as much or as little time as you would like to consider opposing perspectives. **One thing you should keep in mind is that we will tell Player 1 how long you spend considering opposing perspectives before deciding whether to sign.**

UCF professor behind tweets deemed racist says he is subject of 'witch hunt'

By Annie Martin Orlando Sentinel (TNS) Jun 18, 2020

Note: the above screenshot shows the screen from the "Both Observable" condition, where looking was observable.

Democrats, Moore petition (Study 3a):

Additionally, **we will give you an opportunity to take some time—if you would like—to consider OPPOSING perspectives before deciding whether to sign.** It is completely up to you whether to use this time, and how.

For your convenience, we will provide you with links to some specific articles that may provide **opposing** perspectives pertaining to the petition. For example, we will provide a link to the below article published by the LA Times describing how elected politicians in Los Angeles, including the mayor, continue to support Police Chief Moore. You can also choose to use the time to search the Internet for other opposing perspectives.

Importantly, you can also choose NOT to read any opposing perspectives and instead move forward with the survey.

You can take as much or as little time as you would like to consider opposing perspectives. **One thing you should keep in mind is that your decision will be completely private: we will NOT tell Player 1 how long you spend considering opposing perspectives before deciding whether to sign.**

Sections Los Angeles Times LOG IN

CALIFORNIA

Despite criticism, LAPD Chief Michel Moore maintains support in political circles

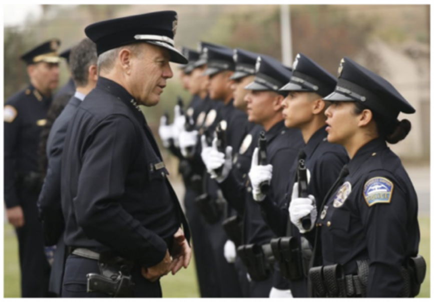

Police Chief Michel Moore continues to enjoy the support of Los Angeles officials. (Al Seib / Los Angeles Times)

Republicans, Amazon Petition (Studies 3b and 4b):

Additionally, **we will give you an opportunity to take some time—if you would like—to consider OPPOSING perspectives before deciding whether to sign.** It is completely up to you whether to use this time, and how.

For your convenience, we will provide you with links to some specific articles that may provide **opposing** perspectives pertaining to the petition. For example, we will provide a link to the below article, published by a local Houston news outlet, reporting on the perspective of an activist who does not think “Blue Lives Murder” merchandise constitutes hate speech. You can also choose to use the time to search the Internet for other opposing perspectives.

Importantly, you can also choose NOT to read any opposing perspectives and instead move forward with the survey.

You can take as much or as little time as you would like to consider opposing perspectives. **One thing you should keep in mind is that your decision will be completely private: we will NOT tell Player 1 how long you spend considering opposing perspectives before deciding whether to sign.**

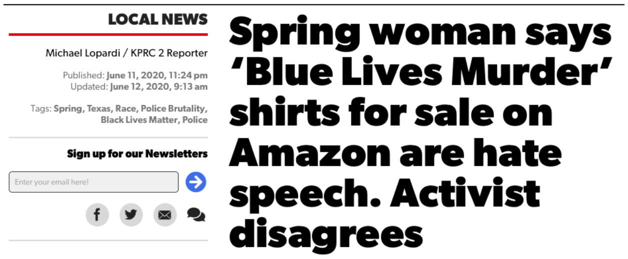
LOCAL NEWS

Michael Lopardi / KPRC 2 Reporter

Published: June 11, 2020, 11:24 pm
Updated: June 12, 2020, 9:13 am

Tags: Spring, Texas, Race, Police Brutality, Black Lives Matter, Police

Sign up for our Newsletters

Enter your email here!

Spring woman says ‘Blue Lives Murder’ shirts for sale on Amazon are hate speech. Activist disagrees

Note: the above two screenshots show the screens from the “Nothing Observable” and “Punishment Observable” conditions, where looking was not observable.

Next, we summarized for subjects the relevant information about their observability condition, and presented a second set of comprehension questions about what was observable to Player 1. This screen looked different across conditions.

Nothing Observable:

Thank you. On this page, we'd like to review how the petition and Sharing Game components of this HIT relate to each other.

In the Sharing Game, before deciding how much to share with you, Player 1 will see the full text of the petition and learn that you had the opportunity to sign it. Furthermore, Player 1 will learn that you were given time to consider **opposing** perspectives before deciding whether to sign the petition.

Importantly, however, we will NOT tell Player 1 how long you spend considering opposing perspectives, OR whether or not you ultimately decide to sign the petition.

In other words, your signing decision--and the time you spend considering opposing perspectives--will both remain completely private.

Please answer the following questions, to make sure you understand.

Before Player 1 decides how much money to share with you…

Will Player 1 learn that you have the opportunity to sign the petition?

- ◯ No
- ◯ Yes, but Player 1 will not get to read the full text of the petition
- ◯ Yes, and Player 1 will also get to read the full text of the petition

Will Player 1 learn that you were given time to consider **opposing** perspectives before committing to signing?

- ◯ No
- ◯ Yes

What will Player 1 learn about your behavior?

- ◯ Nothing. Player 1 will not find out how long I spend considering opposing perspectives, or whether I ultimately sign the petition
- ◯ Player 1 will only find out whether I ultimately sign the petition (but not how long I spend considering opposing perspectives)
- ◯ Player 1 will both find out how long I spend considering opposing perspectives, and whether I ultimately sign the petition

In Punishment Observable, we presented the same questions, but introduced them as follows:

Thank you. On this page, we'd like to review how the petition and Sharing Game components of this HIT relate to each other.

In the Sharing Game, before deciding how much to share with you, Player 1 will see the full text of the petition and learn that you had the opportunity to sign it. Furthermore, Player 1 will learn that you were given time to consider **opposing** perspectives before deciding whether to sign the petition.

Importantly, we will NOT tell Player 1 how long you spend considering opposing perspectives. We WILL, however, tell Player 1 whether or not you ultimately decide to sign the petition.

In other words, while the time you spend considering opposing perspectives will remain completely private, your signing decision will be shared with Player 1.

In Both Observable, we likewise presented the same questions, but introduced them as follows:

Thank you. On this page, we'd like to review how the petition and Sharing Game components of this HIT relate to each other.

In the Sharing Game, before deciding how much to share with you, Player 1 will see the full text of the petition and learn that you had the opportunity to sign it. Furthermore, Player 1 will learn that you were given time to consider **opposing** perspectives before deciding whether to sign the petition.

And, importantly, we will tell Player 1 how long you spend considering opposing perspectives, AND whether or not you ultimately decide to sign the petition.

In other words, your signing decision--and the time you spend considering opposing perspectives--will both be shared with Player 1.

Like with the first set of comprehension questions, if subjects answered any questions on this screen incorrectly, they were presented with the screen a second time, along with the text: "On the previous page, you answered one or more questions incorrectly. You MUST answer ALL questions correctly to continue on with the study. Please carefully re-read the below instructions and answer the questions again". Here, they were not allowed to precede to the next screen until they answered all questions correctly.

Next, we informed subjects that they were about to precede to the page where they could look at opposing perspectives:

Thank you.

On the next screen, we will give you the opportunity to take some time—if you would like—to consider OPPOSING perspectives before deciding whether to sign the petition.

As a reminder, it is completely up to you whether to use this time, and how. We will provide you with links to some specific articles that may provide **opposing** perspectives pertaining to the petition. You can also choose to use the time to search the Internet for other opposing perspectives.

Importantly, you can also choose NOT to read any opposing perspectives and instead move forward with the survey.

Remember, we will tell Player 1 how long you choose to spend considering opposing perspectives before deciding whether to sign the petition. So Player 1 will learn whether you make a quick decision about whether to sign the petition, or choose to extensively consider opposing perspectives first.

Note: the above screenshot shows the screen from "Both Observable". In the other two conditions, the last paragraph instead read: "Remember, we will NOT tell Player 1 how long you choose to spend considering opposing perspectives before deciding whether to sign the petition. So Player 1 will NOT learn whether you make a quick decision about whether to sign the petition, or choose to extensively consider opposing perspectives first".

Next, we measured looking by presenting the following screens, and measuring which, if any, links subjects clicked on. Subjects were not informed that their link-clicking would be tracked.

Democrats, Negy petition (Study 4a):

Please take as much or as little time as you would like to consider opposing perspectives.

Remember, we will tell Player 1 how long you spend on this page considering opposing perspectives.

When you have decided whether to sign the petition, please advance the screen.

Previously, we showed you the headline of the below article, published by the Orlando Sentinel, describing professor Negy's claim that he is the subject of a "witch hunt". The link to view this article is here.

UCF professor behind tweets deemed racist says he is subject of 'witch hunt'

By Annie Martin Orlando Sentinel (TNS) Jun 18, 2020

Below is the headline of another article you may consider, published by the National Association of Scholars, arguing that firing professor Negy would be a violation of his rights. The link to view this article is here.

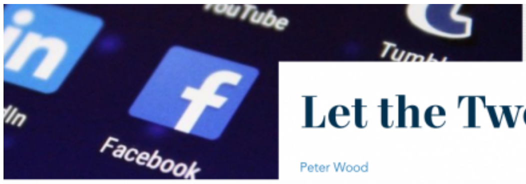

Let the Tweeter Beware

Peter Wood

Article
June 08, 2020
Academic Freedom College Presidents

The New York Times reports that the University of Central Florida is investigating tenured psychology professor Charles Negy in response to

Note: the above screenshot shows the screen from the "Both Observable" condition, where looking was observable.

Democrats, Moore petition (Study 3a):

Please take as much or as little time as you would like to consider opposing perspectives.

Remember, we will NOT tell Player 1 how long you spend on this page considering opposing perspectives.

When you have decided whether to sign the petition, please advance the screen.

Previously, we showed you the headline of the below article, published by the LA Times, describing the support Chief Moore has maintained in Los Angeles political ciricles since the controversey erupted. The link to view this article is here.

Sections Los Angeles Times LOG IN

CALIFORNIA

Despite criticism, LAPD Chief Michel Moore maintains support in political circles

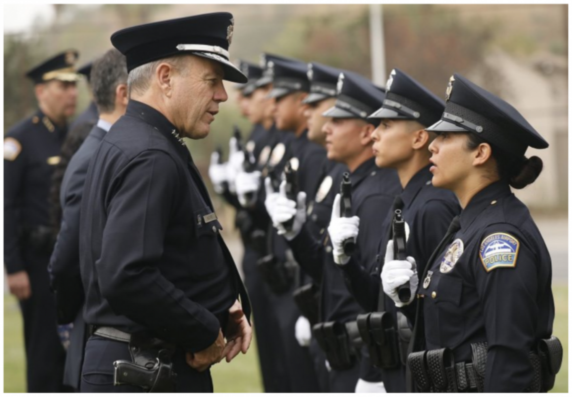

Police Chief Michel Moore continues to enjoy the support of Los Angeles officials. (Al Seib / Los Angeles Times)

Below is the headline of another article you may consider, published by FOX 11 Los Angeles, reporting on Los Angeles Mayor Eric Garcetti's statement of support for Chief Moore. The link to view this article is here.

Garcetti says he has confidence in LAPD Chief Moore after he 'misspoke'

Published June 3 | Death of George Floyd | City News Service

Republicans, Amazon Petition (Studies 3b and 4b):

Please take as much or as little time as you would like to consider opposing perspectives.

Remember, we will NOT tell Player 1 how long you spend on this page considering opposing perspectives.

When you have decided whether to sign the petition, please advance the screen.

Previously, we showed you the headline of the below article, published by Cick2Houston, reporting on the perspective of an activist who does not think “Blue Lives Murder” merchandise constitutes hate speech. The link to view this article is here.

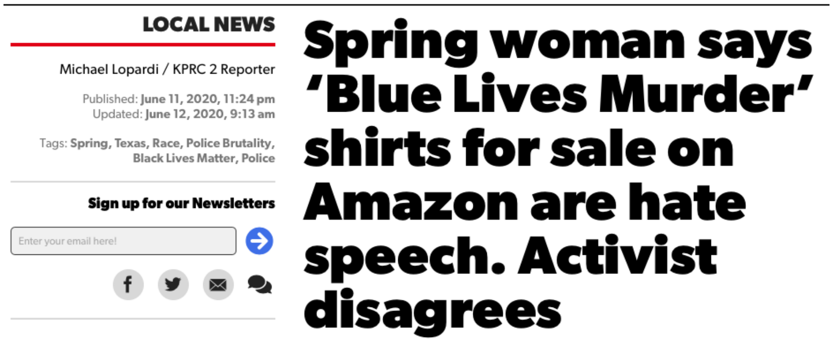

LOCAL NEWS

Michael Lopardi / KPRC 2 Reporter

Published: June 11, 2020, 11:24 pm
Updated: June 12, 2020, 9:13 am

Tags: Spring, Texas, Race, Police Brutality, Black Lives Matter, Police

Sign up for our Newsletters

Enter your email here!

Spring woman says ‘Blue Lives Murder’ shirts for sale on Amazon are hate speech. Activist disagrees

Below is the headline of another article you may consider, published by FIRE, about what constitutes hate speech and the ways that it is protected by the first amendment. The link to view this article is here.

Note: the above two screenshots show the screens from the “Nothing Observable” and “Punishment Observable” conditions, where looking was not observable.

Next, we measured punishment by presenting the following screens, and measuring whether subjects clicked the link to the petition. Subjects were not informed that their link-clicking would be tracked.

On the next page, you will decide whether or not to sign the petition.

If you choose to sign, we will NOT collect your identifying information. Instead, we will ask you to show us that you really did sign another way.

Specifically, immediately after you sign, you will be redirected to a new screen. At the top of this new screen, you will see the 1-2-3 image below. <u>However, instead of seeing the black box, you will see some text.</u> Specifically, you will see a simple phrase, written at the top of the page in relatively small font.

When you see the 1-2-3 image, you will be DONE signing the petition. You do NOT need to take any further action. **Instead, please STAY ON THIS PAGE and pay attention to the phrase that is written above the 1-2-3 image (in place of the black box).**

If you sign the petition, **we will ask you to report that phrase back to us,** to show that you really did sign.

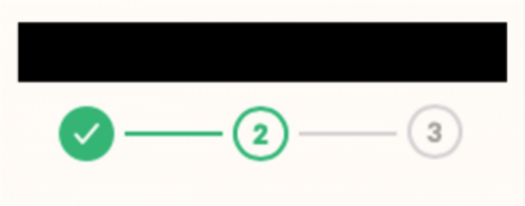

[Page break]

Thank you. Now, you will decide whether or not to sign the petition.

Remember, Player 1 will learn about the petition (and will get to read the full text of the petition). **And we will tell Player 1 whether or not you ultimately chose to sign the petition.**

<u>If you would like to sign, please do so now by clicking here.</u>

Remember, if you sign, **please stay on the subsequent screen and pay attention to the phrase written above the 1-2-3 image!**

Did you choose to sign the petition?

○ Yes

○ No

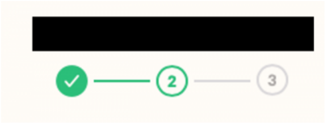

If you DID sign the petition, you should be able to see the 1-2-3 image, pictured above, on the next screen. To show us that you signed, please type the phrase written above the 1-2-3 image (instead of the black box).

If you did NOT sign the petition, please instead just type "no".

Note: if you DID sign the petition, but are confused by the above instructions, please just type any phrase that you saw after signing the petition.

Please also rate your agreement with the following statement:

"I am strongly committed to supporting the petition and the underlying cause behind it."

1 - Strongly disagree	2	3 - Somewhat disagree	4	5 - Neither agree nor disagree	6	7 - Somewhat agree	8	9 - Strongly agree
○	○	○	○	○	○	○	○	○

Note: the above screenshot shows the screens from the “Punishment Observable” and “Both Observable” conditions, where punishment was observable. In “Nothing Observable”, we replaced the sentence that read “And we will tell Player 1 whether or not you ultimate choose to sign the petition” with “But we will NOT tell Player 1 whether or not you sign the petition”.

Finally, subjects completed the following post-experimental survey:

Thank you for your decision. Now, we would like you to answer a few questions about the petition. (Unlike your signing decision, your responses to these questions will NOT be shown to Player 1).

	Not at all 1	2	3	4	I have reservations 5	6	7	8	Very much 9
How moral do you think the petition is?	○	○	○	○	○	○	○	○	○
To what extent do you agree with the petition?	○	○	○	○	○	○	○	○	○
How comfortable are you with the petition's approach?	○	○	○	○	○	○	○	○	○
To what extent do you think the petition's approach is proportionate and appropriate?	○	○	○	○	○	○	○	○	○

[Note: the above screenshot shows the screens from the “Punishment Observable” and “Both Observable” conditions, where punishment was observable. In “Nothing Observable”, the parenthetical instead read: (As was the case for your signing decision, your responses to these questions will NOT be shown to Player 1).]

[Page break]

Thank you. For our records, on the screen where you had the chance to consider opposing perspectives, did you do any of the things listed below?

If so, please check all that apply. If not, that's totally fine; please leave this quesiton blank and advance to the next page.

Note: your response to this question will NOT be shown to Player 1. It is only for our records.

- ☐ I read the Herald Mail Media article (headline: UCF professor behind tweets deemed racist says he is subject of 'witch hunt')
- ☐ I read the National Association of Scholars article (headline: Let the tweeter beware)
- ☐ I searched the Internet for other perspectives that would **oppose** the petition
- ☐ I searched the Internet for other perspectives that would **support** the petition

[Note: the above screenshot shows the screen from Study 4a, where Democrats saw articles about the Negy petition. The text was adapted to reference the correct party and articles for all subjects.]

[Page break]

Thank you.

Before completing this HIT, how much did you know about the events described in the petition?

1 - I had never heard of them	2	3 - I had heard of them, but knew only some details	4	5 - I had been closely following the situation and know many details
○	○	○	○	○

[Page break]

Please describe how you made your choices in this HIT.

[Page break]

Now, we would like to ask you a few questions regarding **the amount of time you decided to spend considering opposing perspectives.**

To what extent did you make your decision (regarding how long to spend considering opposing perspectives) because...

	1 - Not at all	2	3	4	5	6	7 - Entirely
...you personally felt that it was truly the right decision?	○	○	○	○	○	○	○
...you wanted to see yourself as a good person?	○	○	○	○	○	○	○
...you wanted others to see you as a good person?	○	○	○	○	○	○	○
...you wanted Player 1 to see you as a good person?	○	○	○	○	○	○	○

Now, we would like to ask you a few questions about your decision regarding **whether to sign the petition**.

To what extent did you make your decision (regarding whether to sign the petition) because...

	1 - Not at all	2	3	4	5	6	7 - Entirely
...you personally felt that it was truly the right decision?	○	○	○	○	○	○	○
...you wanted to see yourself as a good person?	○	○	○	○	○	○	○
...you wanted others to see you as a good person?	○	○	○	○	○	○	○
...you wanted Player 1 to see you as a good person?	○	○	○	○	○	○	○

[Page break]

To what extent do you believe that the petition and articles providing opposing perspectives are real?

1 - Very skeptical that they are real	2	3	4	5	6	7 - Very confident that they are real
○	○	○	○	○	○	○

To what extent do you believe that Player 1 and the sharing game are real?

1 - Very skeptical that they are real	2	3	4	5	6	7 - Very confident that they are real
○	○	○	○	○	○	○

[Page break]

Dog is to puppy as cat is to ______

In general, how conservative or liberal do you consider yourself to be?

1 - Very liberal	2	3	4	5 - Neither liberal nor conservative	6	7	8	9 - Very conservative
○	○	○	○	○	○	○	○	○

How much loyalty do you feel towards the Democrat party?

1 - Very little	2	3	4	5 - A moderate amount	6	7	8	9 - A large amount
○	○	○	○	○	○	○	○	○

To what extent do you support Black Lives Matter?

1 - Strongly oppose	2	3	4	5 - Neither support nor oppose	6	7	8	9 - Strongly support
○	○	○	○	○	○	○	○	○

Highest level of education completed:

- ○ Less than a high school degree
- ○ High School Diploma
- ○ Vocational Training
- ○ Attended College
- ○ Bachelor’s Degree
- ○ Graduate Degree
- ○ Unknown

Please choose the category that describes the total amount of income you earned in 2019. Consider all forms of income, including salaries, tips, interest and dividend payments, scholarship support, student loans, parental support, social security, alimony, and child support, and others.

- ○ Under $5,000
- ○ $5,000-$10,000
- ○ $10,001-$15,000
- ○ $15,001-$25,000
- ○ $25,001-$35,000
- ○ $35,001-$50,000
- ○ $50,001-$65,000
- ○ $65,001-$80,000
- ○ $80,001-$100,000
- ○ Over $100,000

Please specify your race. (*Choose one or more categories)*

- ☐ White/Caucasian (Anglo/Euro) American
- ☐ Black or African American
- ☐ Asian or Asian American
- ☐ American Indian or Alaska Native
- ☐ Native Hawaiian or other Pacific Islander
- ☐ Hispanic/Latino
- ☐ Multicultural

Note: the above screenshot shows the screen for Democrats in Studies 3a and 4a, where we measured support for Black Lives Matter; for Republicans in Studies 3b and 4b, we referenced the Republican party (rather than the Democrat party) and measured support for Blue Lives Matter (“To what extent do you support "Blue Lives Matter" (a countermovement, started in response to Black Lives Matter, advocating that those who are prosecuted and convicted of killing law enforcement officers should be sentenced under hate crime statutes)?”).
